# Bacterial targeting of the neutrophil inhibitory receptor LILRB3 to evade antibody immunity

**DOI:** 10.1038/s41467-026-74098-6

**Published:** 2026-06-11

**Authors:** Matevž Rumpret, Alexander L. Lewis Marffy, Ying Zhang, Esther van Woudenbergh, Sjors P. A. van der Lans, Xin Xu, Zuyi Fu, Yuxi Zhao, Erin A. Catton, Guillaume Paré, Felix McGregor, Owen B. Spiller, Maria J. Fernandes, Carla J. C. de Haas, Jos A. G. van Strijp, Nina M. van Sorge, Brian V. Geisbrecht, Alex J. McCarthy

**Affiliations:** 1 Centre for Bacterial Resistance Biology (CBRB), Department of Infectious Disease, Imperial College London, London, UK; 2 Department of Biochemistry and Molecular Biophysics, Kansas State University, Manhattan, KS USA; 3 Department of Medical Microbiology, University Medical Center Utrecht, Utrecht University, Utrecht, the Netherlands; 4 Department of Microbiology and Immunology, Faculty of Medicine, Université Laval, Québec City, QC Canada; 5 Division of Rheumatology, Department of Medicine, CHU de Québec-Université Laval Research Center, Québec City, QC Canada; 6 Department of Medical Microbiology, Division of Infection and Immunity, Cardiff University, 6th Floor University Hospital of Wales, Cardiff, UK; 7 Bacterial Reference Department, UK Health Security Agency, London, UK; 8 Department of Medical Microbiology and Infection Prevention, Amsterdam Institute for Immunology and Infectious Diseases, Amsterdam UMC, University of Amsterdam, Amsterdam, The Netherlands; 9 Netherlands Reference Laboratory for Bacterial Meningitis, Amsterdam UMC, Amsterdam, The Netherlands

**Keywords:** Pathogens, Infection, Immune evasion

## Abstract

Antibody-mediated responses are critical for antibacterial immunity, driving Fc receptor-mediated phagocytosis, respiratory burst, and bacterial killing. Inhibitory immune receptors regulate cellular activation and immune homoeostasis, but whether they are targeted to evade antibody-mediated responses is unclear. Here, we show that the highly expressed inhibitory LILRB3 receptor on neutrophils is targeted by *Streptococcus agalactiae* to suppress antibody-driven defence. We show that the surface-localised β protein of *S. agalactiae* bound to LILRB3 and induced its cross-linking. β and LILRB3 interactions suppressed Fc receptor-mediated antibacterial responses such as respiratory burst and bacterial killing, inhibiting antibody-dependent bacterial killing by neutrophils. Moreover, β expression and LILRB3 targeting are associated with *S. agalactiae* lineages that cause invasive diseases in older adults, indicating that targeting inhibitory receptors most likely provides an immune evasion mechanism when antibody responses are waning, such as in immunocompromised or elderly individuals. Altogether, these results highlight a simple and important mechanism that bacterial pathogens utilise to overcome effective antibody-driven phagocyte responses.

## Abstract (web summary)

Antibody-mediated immunity is essential to protect hosts from bacterial pathogens. Here, the authors show that *Streptococcus agalactiae* targets inhibitory LILRB3 receptor on neutrophils to supress antibody-driven defence.

## Introduction

Antibody-mediated immunity from natural exposure or vaccination is important to protect hosts from bacterial pathogens. Opsonic IgG antibodies elicit protection through two key mechanisms: they trigger the classical complement pathway to deposit C3b on bacterial surfaces to facilitate complement receptor-mediated bacterial uptake and killing, and they activate Fc-gamma receptors (FcγR) on neutrophils and other phagocytes to induce bacterial uptake and killing^1^. However, newborns lack fully matured antibodies, and the elderly and immunocompromised individuals experience a decline in the function of immune cells generating and responding to antibodies^2,3^. As a result, these populations, and those with primary antibody deficiencies, are more susceptible to bacterial infections^4–9^. As a countermeasure to antibody-mediated responses, bacterial pathogens have often evolved mechanisms to avoid detection, uptake and killing by phagocytic immune cells^10^. Currently, described mechanisms of bacterial antibody evasion include hiding antigenic structures, modifying antibodies through proteases or enzymes, expressing molecules that bind Fc regions of antibodies to neutralise their functions and producing molecules to block FcγR^11–14^. These strategies promote the progression of disease in experimental infection models and likely further compromise the already diminished antibody-mediated responses in vulnerable human populations, contributing to bacterial pathogenesis. However, antibody evasion mechanisms are not understood for all human bacterial pathogens. This is essential for the design of vaccines and antibody-based therapies that are maximally effective, especially in vulnerable hosts.

**Fig. 1:**
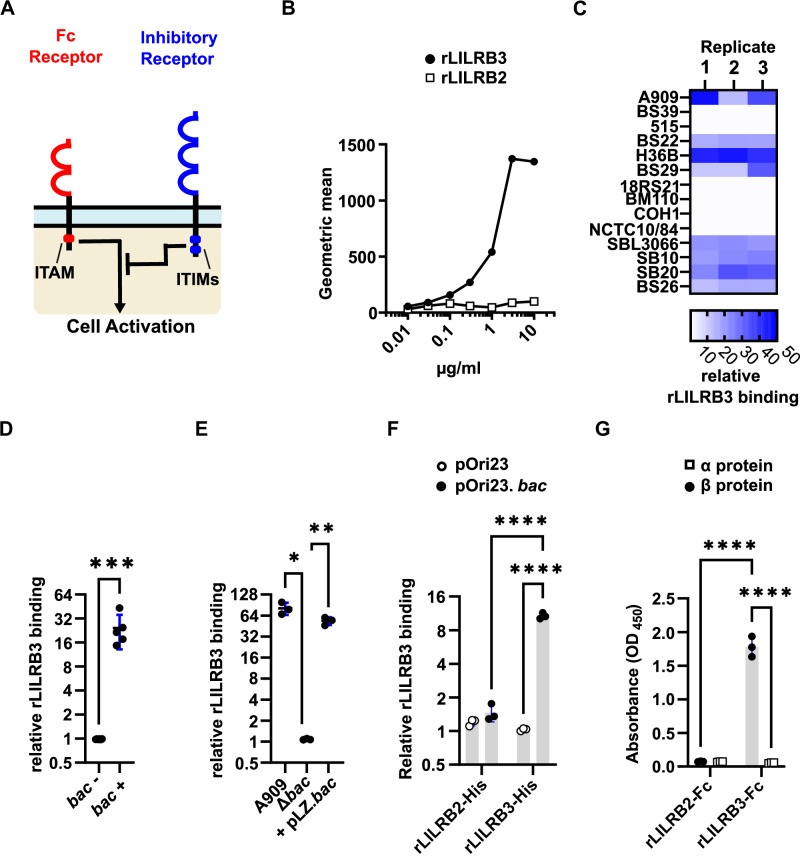
*S. agalactiae* binds human LILRB3 via β protein. **A** Schematic of inhibitory receptor regulation of Fc receptor (FcR)-mediated cell activation. Inhibitory receptors contain the extracellular region (composed of Ig-like domains), a transmembrane region and a cytoplasmic tail containing immunoreceptor tyrosine-based inhibitory motifs (ITIMs) for signalling. FcRs contain an extracellular region (composed of Ig-like domains), a transmembrane region and a cytoplasmic tail containing immunoreceptor tyrosine-based activation motifs (ITAM), or associate with ITAM-bearing receptors, for signalling. **B** Concentration-dependent binding of rLILRB3-His or rLILRB2-His to *S. agalactiae* strain A909, quantified using flow cytometry (*n* = 2 independent experiments). **C** Binding of rLILRB3-His (10 μg/ml) to *S. agalactiae* strains, quantified using flow cytometry. Each heat map displays the relative rLILRB3-binding compared to the detection antibody (since these bind differently to strains) per strain for *n* = 3 independent experiments. **D** Binding of rLILRB3-His (10 μg/ml) to *bac*− and *bac* + *S. agalactiae* strains, quantified using flow cytometry. Each data point represents one strain. Data is displayed as relative rLILRB3-binding compared to secondary antibody control stain, and each value represents the mean of *n* = 3 independent experiments. Mean ± s.d across strains is shown, with an unpaired two-sided Student’s *t* test (***p = 0.0006). **E** Binding of rLILRB3-His (10 μg/ml) to isogenic *S. agalactiae* A909 strains, quantified using flow cytometry. Data is displayed as relative rLILRB3-binding compared to secondary antibody control stain. Mean ± s.d. of *n* = 3 independent experiments, with one-way ANOVA (A909 vs. Δ*bac* *p = 0.023, Δ*bac* vs. Δ*bac* + pLZ. bac p** = 0.01). **F** Binding of rLILRB3-His (10 μg/ml) to isogenic *L. lactis* strains, quantified using flow cytometry. Data is displayed as relative rLILRB3-binding compared to control. Mean ± s.d. of *n* = 4 independent experiments, with two-way ANOVA where pOri23.*bac*/LILRB2 vs pOri23.*bac*/LILRB3 ****p < 0.0001. and pOri23/LILRB3 vs pOri23.*bac*/LILRB3 ****p< 0.0001. **G** rLILRB3-Fc binding to purified coated-β protein, quantified using ELISA using HRP-conjugated anti-human-IgG. Mean ± s.d. of *n* = 3 indepe*n*dent experiments, with one-way ANOVA where β/LILRB2 vs. β/LILRB3 ****p < 0.0001 and α/LILRB3 vs. β/LILRB3 ****p < 0.0001.

Inhibitory receptors negatively regulate the activation and functions of immune cells to support immune homoeostasis^15,16^. These cell surface receptors contain immunoreceptor tyrosine-based inhibitory motifs (ITIMs) in their cytoplasmic tails that, upon phosphorylation, recruit tyrosine phosphatase molecules (such as SHP-1, SHP-2 and SHIP). The recruitment of these tyrosine phosphatase molecules to inhibitory receptors interferes with the activating signals transduced by immunoreceptor tyrosine-based activation motifs (ITAMs) in FcγR and Fc-alpha receptor (FcαR) [Fig. 1A]^17^. Several inhibitory receptors, specifically CD300A, LILRB2, and LILRB3, have been shown to negatively regulate FcγR- and/or FcαR-induced activation and functions of neutrophils and other phagocytic immune cells^16,18–24^. Moreover, human inhibitory receptors are also engaged by several bacterial pathogens^25,26^. Despite this, the critical question of whether pathogenic bacteria target human inhibitory receptors to suppress antibody- and Fc receptor (FcR)-mediated antibacterial responses of phagocytes and thereby promote their own survival remains unanswered.

Highly expressed inhibitory receptors often induce stronger suppressive signals^27^. We therefore screened human bacterial pathogens for their binding to these inhibitory receptors and found that *Streptococcus agalactiae* (also known as Group B *Streptococcus*) interacted with the human inhibitory LILRB3 receptor through a surface protein called β protein. *S. agalactiae* is a major cause of invasive infections in neonates, immunocompromised adults and the elderly^28,29^. Often, these invasive infections occur in individuals with low immunoglobulin titres against *S. agalactiae*^30,31^. Using the β-LILRB3 interaction as a tool to delineate whether bacterial targeting of human inhibitory receptors suppresses antibacterial immune responses, we demonstrate that β protein targets LILRB3 to suppress FcγR- and FcαR-mediated activation of neutrophils, which in turn suppresses killing of *S. agalactiae*. Population genetic analysis of *S. agalactiae* revealed that the β protein-encoding gene called *bac* is carried in two lineages that predominantly cause invasive disease in non-pregnant adult populations, indicating that the targeting of inhibitory receptors to suppress FcγR-mediated responses favours the pathogen when antibody titres are lower or have reduced functionality. To our knowledge, these data reveal that targeting of human inhibitory receptors by bacterial pathogens represents a mechanism to subvert antibody-dependent phagocyte responses, illustrating a potentially widespread mechanism by which bacterial pathogens neutralise antibody-dependent immunity.

## Results

### LILRB3 binds to *S. agalactiae* expressing β protein

Previous analysis has identified that several inhibitory receptors are highly expressed on the surface of circulating neutrophils, including CD200R, LILRB3, Siglec-5, Siglec-9, and SIRL-1,^18,32–35^, making these inhibitory receptors prime candidates for exploitation by bacterial pathogens and for our investigation. This contrasts with other inhibitory receptors that are stored in granules, such as LILRB2, CD300a and CEACAM1, and upregulated to the surface upon cellular activation^20,22,36^. Of the inhibitory receptors expressed on circulating neutrophils, we focused our investigation on LILRB3 because human bacterial pathogens interact with this inhibitory receptor through undefined mechanisms^18,37^, and because accumulating evidence indicates that LILRB3 plays a key role in the negative regulation of myeloid cell activation^18,38^, rendering it a potential target for pathogen hijacking. Human LILRB3 is composed of an extracellular region containing four Ig-like domains (called D1, D2, D3 and D4), a transmembrane region and a cytoplasmic region containing four ITIMs. LILRB3 is reported to recognise the endogenous ligands Galectin-4, Galectin-7, Apolipoprotein E3 and Apolipoprotein E4^39,40^. We first clarified that LILRB3 is co-expressed with Fc receptors on the surface of circulating neutrophils [Supplementary Fig. 1A]. Our screening for binding of recombinant (r)LILRB3 to a number of invasive bacterial pathogens found it bound in a concentration-dependent manner to a *S. agalactiae* strain called A909 [Fig. 1B]. In contrast, the closely related inhibitory LILRB2 receptor did not bind to this *S. agalactiae* strain. Further analysis revealed that rLILRB3 bound to a broader repertoire of *S. agalactiae* strains [Fig. 1C], indicating that a subset of the *S. agalactiae* population has evolved a specific mechanism to interact with LILRB3.

We next aimed to identify the surface molecule of *S. agalactiae* that interacts with LILRB3. Importantly, we noticed the subset of *S. agalactiae* strains that interacted with rLILRB3 were the same strains that interacted with human CEACAM receptors through the expression of a surface-localised protein called β protein, encoded by the *bac* gene^41^. Notably, *bac*-positive *S. agalactiae* strains displayed a significantly increased level of rLILRB3 binding compared to *bac*-negative strains [Fig. 1D]. The binding of rLILRB3 to *S. agalactiae* depended on β protein expression, as the interaction with A909 was significantly reduced in a Δ*bac* strain, but recapitulated in a complemented Δ*bac* strain [Fig. 1E]. We utilised non-pathogenic *Lactococcus lactis* as a heterologous expression system to assess the properties of β protein to ensure that our analysis was not confounded by other *S. agalactiae* factors. Notably, rLILRB3 bound to the surface of a *L. lactis* strain engineered to express the *S. agalactiae* β protein at the bacterial cell surface, but not to a control *L. lactis* strain [Fig. 1F]. Together, these data indicate that β protein is necessary and sufficient for interaction with human LILRB3.

We hypothesised that β protein directly interacted with LILRB3. In line, rLILRB3 bound to microtitre plate wells coated with purified β protein in ELISA assays but not wells coated with a control *S. agalactiae* surface protein called α protein [Fig. 1G]. Additionally, in an assay testing the interaction in the reverse orientation, β protein bound to dynabeads (DB) coated with rLILRB3-His, but not to DB coated with rLILRB2-His [Supplementary Fig. 1B]. In conclusion, *S. agalactiae* interacts directly with the inhibitory receptor LILRB3 through expression of β protein at its surface.

### β protein directly cross-links LILRB3 and inhibits FcR-mediated neutrophil activation

**Fig. 2:**
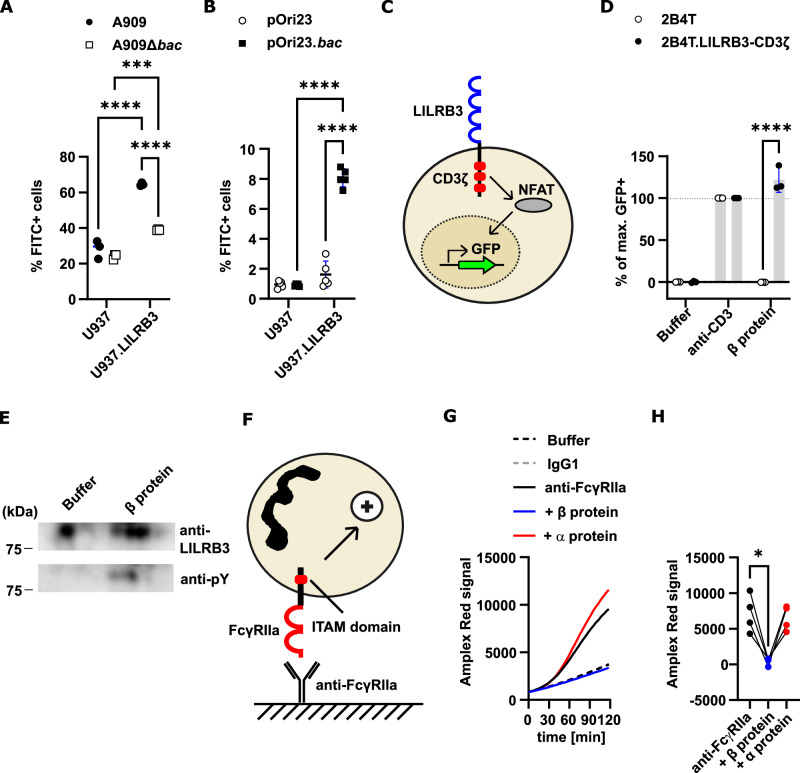
β protein directly cross-links LILRB3 and inhibits FcR-mediated activation of human neutrophils. **A** Adhesion of FITC-labelled *S. agalactiae* A909 strains to U937 cell lines, quantified using flow cytometry. Mean ± s.d. of *n* = 3 independent experiments, with two-way ANOVA including Šídáks multiple comparison test, where A909/U937 vs A909/U937.LILRB3 ****p < 0.0001, A909/U937.LILRB3 vs A909Δ*bac*/U937.LILRB3 ****p< 0.0001 and A909Δ*bac*/U937 vs A909Δ*bac*/U937.LILRB3 ***p = 0.001. **B** Binding of FITC-labelled *L. lactis* strains (carrying pOri23 or pOri23.*bac* encoding wildtype β) to U937 cell lines, quantified using flow cytometry. Mean ± s.d. of *n* = 3 independent experiments, with two-way ANOVA where U937/pOri23.*bac* vs U937.LILRB3/pOri23.*bac* ****p < 0.0001 and U937.LILRB3/pOri23 vs U937.LILRB3/pOri23.*bac* ****p < 0.0001. **C** Schematic of the LILRB3-CD3ζ reporter 2B4T cell line. Cross-linking of a fusion receptor formed of the extracellular and transmembrane regions of LILRB3 and the cytoplasmic tail of CD3ζ induces phosphorylation of immunoreceptor tyrosine-based activation motifs (ITAMs; shown in red), inducing the transcription factor nuclear factor of activated T-cells (NFAT) to express green fluorescent protein (GFP). **D** Stimulation of 2B4T reporter cells after incubation with rβ protein, quantified using flow cytometry. The percentage of GFP-positive cells was calculated and normalised against cells stimulated with anti-CD3. Mean ± s.d. of *n* = 3 independent experiments, with two-way ANOVA with Šídáks multiple comparison test, where 2B4T/β protein vs. 2B4T.LILRB3/β protein ****p < 0.0001. **E** Immunoblot analyses showing increased tyrosine phosphorylation of LILRB3 after THP-1 cells were treated with β protein. LILRB3 was detected using anti-LILRB3. Tyrosine phosphorylated proteins were detected with anti-phosphotyrosine (anti-pY). Representative image of *n* = 2 independent replicates. **F** Neutrophil FcR-dependent respiratory burst assay schematic. Cross-linking of FcR, such as FcγRIIa, induces phosphorylation of ITAMs (shown in red) and signal transduction that activates reactive oxygen species (ROS) production, quantified by fluorescence using AmplexRed. **G**, **H** Modulation of FcγRIIa-mediated respiratory burst in human neutrophils by purified β protein, quantified using Amplex Red. In **G**, a representative Amplex Red Signal plot is shown. In **H**, the consolidated data for *n* = 4 independent experiments are shown, where the Amplex Red signal at 120 min is shown after subtraction of the signal from IgG1-stimulation. Mean ± s.d. is shown, with a two-sided *t*-test where anti-FcγRIIa vs. anti-FcγRIIa/β *p = 0.0229.

We next investigated the functional consequences of the β-LILRB3 interaction. First, we tested whether β-LILRB3 interactions enhanced binding of bacteria to cells. The wild-type *S. agalactiae* A909 strain, but not the A909Δ*bac* strain, displayed enhanced adhesion to LILRB3-expressing U937 cells compared to control U937 cells [Fig. 2A], indicating that β protein promotes LILRB3-dependent cellular adhesion. Similarly, *L. lactis* expressing β protein displayed significantly enhanced binding to U937 cells over-expressing LILRB3 compared to control U937 cells or wild-type *L. lactis* [Fig. 2B]. Thus, bacteria can enhance their adhesion to immune cells by targeting LILRB3. This could provide opportunities to cross-link this inhibitory receptor to modulate cell signalling, activation and function.

To investigate whether β protein induced LILRB3 cross-linking, we used our established LILRB3-CD3ζ reporter 2B4T murine cells^18^. In this system, ligand binding and subsequent cross-linking of LILRB3 trigger signalling through the CD3ζ component, resulting in GFP expression as a readout for inhibitory receptor activation [Fig. 2C]. Plate-bound purified β protein elicited GFP expression in the LILRB3-CD3ζ chimaera, but not the parental cells [Fig. 2D]. Furthermore, β-expressing *S. agalactiae*, but not the isogenic Δ*bac* strain, induced GFP expression in the LILRB3-CD3ζ reporter cells, but not the control cells [Supplementary Fig. 1C]. Since phosphorylation of ITIMs is crucial for LILRB3 and other inhibitory receptors to transduce inhibitory signals^42^, we tested whether β protein induced tyrosine phosphorylation of LILRB3 in THP-1 cells. The β protein induced LILRB3 tyrosine phosphorylation [Fig. 2E]. These experiments confirm that *S. agalactiae* crosslinks and phosphorylates LILRB3 through β protein.

We hypothesised that the interaction of β protein with LILRB3 on neutrophils would suppress FcγR-induced neutrophil activation through recruitment of tyrosine phosphatases to the phosphorylated ITIMs of LILRB3, thereby suppressing activating signals emanating from ITAMs of FcγR or associated FcγR chains^15,16,42^. To test this hypothesis and assess the impact of the newly discovered interaction on neutrophil function, we stimulated FcγRIIa (CD32a) on neutrophils with a monoclonal antibody and measured their ROS production [Fig. 2F]. Co-stimulation of neutrophils with anti-FcγRIIa and β protein suppressed ROS production compared to anti-FcγRIIa alone [Fig. 2G, H]. Similar inhibitory effects were identified when we stimulated FcαRI (CD89) on neutrophils [Supplementary Fig. 1D, E]. These results indicate that a bacterial protein capable of cross-linking LILRB3 inhibits FcR-mediated neutrophil activation.

### Two domains in β protein interact with LILRB3

**Fig. 3:**
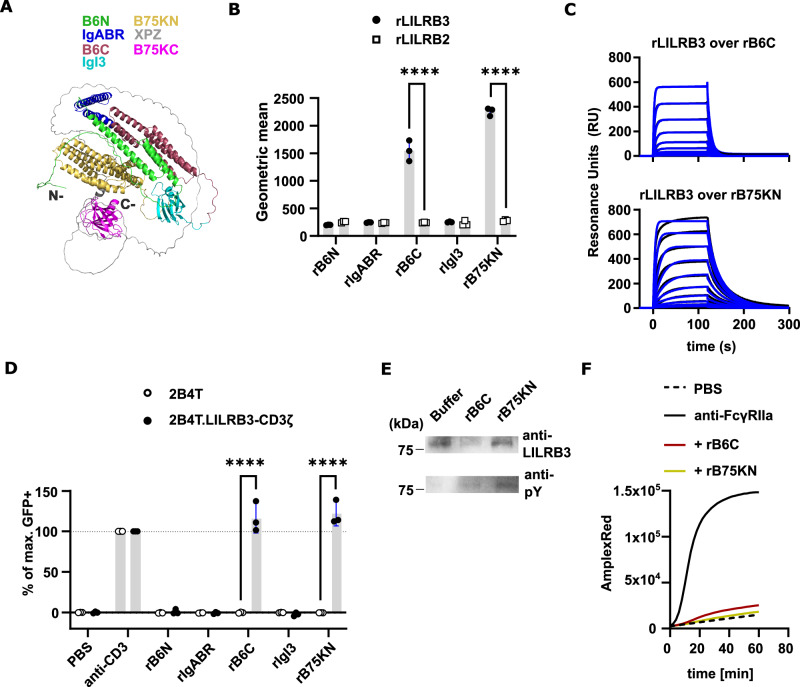
Two domains of the β protein bind LILRB3 to suppress FcR-mediated ROS production by neutrophils. **A** AlphaFold prediction for β protein (Accession: AF-P27951-F1), with individual domains coloured as indicated. The N-terminus and C-terminus of the protein structure is denoted by ‘N-’ and ‘C-’. **B** Binding of rLILRB3-Fc and rLILRB2-Fc to rB6N-, rIgABR-, rB6C-, rIgI3- and rB75KN-coated Dynabeads, quantified using flow cytometry and using PE-conjugated anti-human-IgG. The mean ± s.d. of *n* = 3 independent experiments is shown, with two-way ANOVA with Šídáks multiple comparison test, where rLILRB2/B6C vs. rLILRB3/B6C p**** < 0.0001 and rLILRB2/B75KN vs. rLILRB3/B75KN p****< 0.0001. **C** Reference-corrected surface plasmon resonance (SPR) series for binding of rLILRB3 to immobilsed rB6C and rB75KN, showing both the experimental data (black traces) and the fit to a one-to-one kinetic model (blue traces). **D** Stimulation of GFP production in 2B4T reporter cells by rB6N, rIgABR, rB6C, rIgI3 and rB75KN, quantified using flow cytometry. The percentage of GFP-positive cells was calculated and normalised against cells stimulated with anti-CD3. The mean ± s.d. of *n* = 3 independent experiments with two-way ANOVA with Šídáks multiple comparison test, where B6C/2B4T vs. B6C/2B4T.LILRB3CD3ζ ****p < 0.0001 and where B75KN/2B4T vs B75KN/2B4T.LILRB3CD3ζ ****p < 0.0001. **E** Immunoblot analyses showing increased tyrosine phosphorylation of LILRB3 after THP-1 cells were treated with rB6C or rB75KN. Tyrosine phosphorylated proteins were detected with anti-phosphotyrosine (4G10) antibody. Representative image of *n* = 2 independent replicates. **F** Modulatio*n* of FcγRIIa-mediated respiratory burst in human neutrophils by rB6C and rB75KN, quantified using Amplex Red. A representative Amplex Red Signal plot for *n* = 3 independent experiments is shown.

The predicted β protein structure contains several structurally distinct domains [Fig. 3A and Supplementary Fig. 2A–C]. Some of these domains are known to engage other components of the human immune system, including complement factor H, IgA and the immune receptors Siglec-5, Siglec-7, Siglec-14, CEACAM1 and CEACAM5^41,43–46^ [Supplementary Fig. 2A]. Considering that such interactions might contribute to any observed immunosuppressive effect of β protein on FcγRIIa-mediated or FcαRI-mediated activation of neutrophils, we aimed to identify the domain(s) of β protein that interacted with LILRB3. Therefore, we mapped the binding of rLILRB3-Fc to recombinant β protein domains (B6N, IgABR, B6C, IgI3 and B75KN) that we previously purified from *E. coli*^41^. rLILRB3-Fc bound to dynabeads (DB) coated with rB6C or rB75KN, but not to DB coated with any other β protein domains [Fig. 3B and Supplementary Fig. 2D]. In agreement, size exclusion chromatography (SEC) revealed that rLILRB3 formed complexes with rB6C and rB75KN [Supplementary Fig. 2E, F], indicating that relatively high-affinity binding occurs in solution. To gain a more quantitative understanding of the LILRB3/B6C and LILRB3/B75KN interactions, we measured their binding affinity by surface plasmon resonance (SPR) [Fig. 3C and Supplementary Table 1]. These assays revealed that rB6C (*K*_D_ = 400.0 ± 1.5 nM) and rB75KN (*K*_D_ = 131.0 ± 0.8 nM) bound to rLILRB3 with similar dissociation constants in the low to mid 10^−7^ M range.

The two different binding sites of β protein for LILRB3 raised the question of whether both domains can transduce inhibitory signals. To test this, we compared the capacity of rB6N, rIgABR, rB6C, rIgI3 and rB75KN to induce GFP expression in LILRB3-CD3ζ reporter cells. Both rB6C and rB75KN, but not the other domains, induced significant GFP expression in the LILRB3-CD3ζ reporter cells compared to the parental cells [Fig. 3D]. Notably, both rB6C and rB75KN were sufficient to induce LILRB3 cross-linking and tyrosine phosphorylation [Fig. 3E]. To directly assess the functional impact of B6C and B75KN on neutrophil activation, we investigated their effect on FcR-mediated respiratory burst. We found that neutrophils stimulated with anti-FcγRIIa and rB6C or anti-FcγRIIa and rB75KN displayed significantly reduced ROS production compared to neutrophils stimulated with FcγRIIa alone [Fig. 3F]. Assays performed with anti-FcαRI stimulation showed a similar effect [Supplementary Fig. 2G]. Collectively, this indicates that the pathogen-derived β protein targets LILRB3 by two different domains (B6C and B75KN) and induces LILRB3 phosphorylation. This biochemical signalling translates into a functional phenotype as IgG/FcγR-dependent and IgA/FcαRI-dependent cellular immune responses are suppressed.

### D3 to D4 on LILRB3 is targeted by both β protein domains

**Fig. 4:**
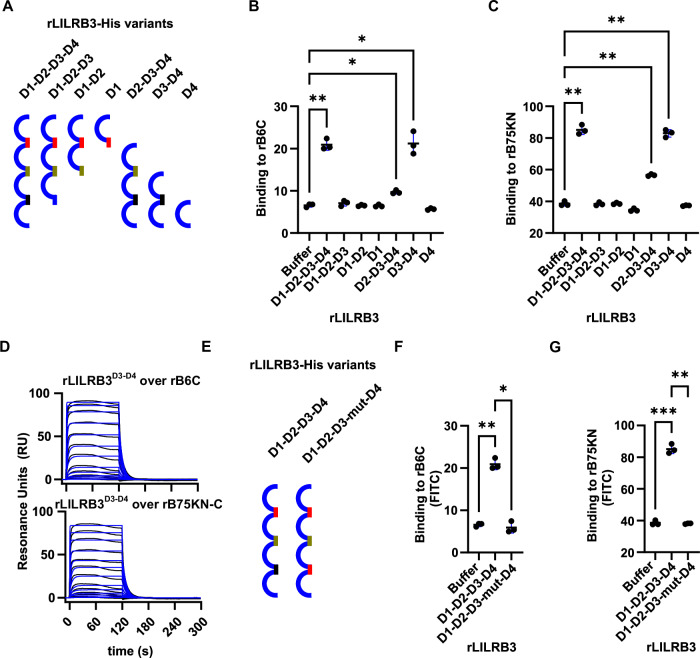
β protein domains B6C and B75KN target D3 to D4 of LILRB3. **A** Schematic of rLILRB3-His variants with Ig domain deletions expressed and purified. **B** Binding of rLILRB3-His variants to rB6C-coated Dynabeads, quantified using flow cytometry. Each rLILRB3-His construct has a varying number of Ig domains (D) as annotated. The mean ± s.d. of *n* = 3 independent experiments, with one-way ANOVA using Tukey’s multiple comparisons, where buffer vs. rLILRB3 **p = 0.0067, buffer vs. rLILRB3^D2-D3-D4^ *p = 0.0206, and buffer vs. rLILRB3^D3-D4^ *p = 0.0239. **C** Binding of rLILRB3-His variants to rB75KN-coated Dynabeads, quantified using flow cytometry and FITC-conjugated anti-6xHis. Each rLILRB3-His construct has varying numbers of Ig domains (D) as annotated. Mean ± s.d. of *n* = 3 independent experiments, with one-way ANOVA using Tukey’s multiple comparisons, where buffer vs. rLILRB3 **p = 0.0011, buffer vs. rLILRB3^D2-D3-D4^ **p = 0.0075, and buffer vs rLILRB3^D3-D4^ **p = 0.0011. **D** Reference-corrected surface plasmon resonance (SPR) series for binding of D3-D4 of LILRB3 to immobilised rB6C and rB75KN-C, showing both the experimental data (black traces) and the fit to a one-to-one kinetic model (blue traces). **E** Schematic of rLILRB3-His variants (wild-type = D1-D2-D3-D4; mutated for replacement of L3 with L1 = D1-D2-D3-mut-D4). **F** Binding of rLILRB3-His variants to rB6C-coated Dynabeads, quantified using flow cytometry. Mean ± s.d. of *n* = 3 independent experiments, with one-way ANOVA with Tukey’s multiple comparisons, where buffer vs. rLILRB3^D1-D2-D3-D4^ **p = 0.0037 and rLILRB3^D1-D2-D3-D4^ vs. rLILRB3^D1-D2-D3-mut-D4^ *p = 0.0134. **G** Binding of rLILRB3-His variants to rB75KN-coated Dynabeads, quantified using flow cytometry. Mean ± s.d. of *n* = 3 independent experiments, with one-way ANOVA with Tukey’s multiple comparisons, where buffer vs. rLILRB3^D1-D2-D3-D4^ ***p = 0.0006 and rLILRB3^D1-D2-D3-D4^ vs. rLILRB3^D1-D2-D3-mut-D4^ **p = 0.0018.

We anticipated that deleting either or both B6C and B75KN from β protein could significantly disrupt the structure of β protein and potentially abrogate other immune interactions. Therefore, we aimed to pinpoint the specific regions that B6C and B75KN bind to LILRB3. To define the LILRB3 region targeted by B6C and B75KN, we generated a series of truncated LILRB3 constructs that each lacked one or more Ig-like domains [Fig. 4A and Supplementary Fig. 3A] and tested their ability to bind rB6C and rB75KN. This revealed that rLILRB3^D3-D4^ (Ig domains 3 and 4 only) bound to DB coated with rB6C [Fig. 4B and Supplementary Fig. 3B] and B75KN [Fig. 4C and Supplementary Fig. 3B]. This interaction was further assessed using SPR, which confirmed that rLILRB3^D3-D4^ bound to rB6C and rB75KN-C (an active fragment of rB75KN described further in the section below), with affinities in the mid-10^−7^ M range (*K*_D_ = 500.2 ± 109.7 nM and *K*_D_ = 307.4 ± 64.3 nM, respectively) [Fig. 4D and Supplementary Table 2]. However, rLILRB3^D3^ or rLILRB3^D4^ alone were unable to interact with either rB6C or rB75KN [Supplementary Fig. 3C, D]. This suggested that both D3 and D4 are contact sites and/or that the loop region between D3 and D4 contributes to the B6C and/or B75KN interactions. To investigate the involvement of the loop region between D3 and D4 in the interaction, hereafter termed L3, we generated a chimeric rLILRB3 variant where L3 between D3 and D4 was replaced with L1 that normally appears between D1 and D2 [Fig. 4E]. This variant did not interact with rB6C [Fig. 4F] or rB75KN [Fig. 4G]. Collectively, these data indicate that both B6C and B75KN domains of β protein interact with D3-D4 of LILRB3 in a manner dependent on the presence of L3.

### Structural basis of β protein and LILRB3 interfaces

To enable the design of targeted point mutations in β protein for disrupting the LILRB3 interaction, we aimed to further define the structural basis of the interactions between the B6C and B75KN domains and the D3-D4 domains of LILRB3. Whilst we previously reported high-resolution structural information on a C-terminal fragment of B75KN that we term here B75KN-C^46^, there was no experimental structural information available for the B6C domain. Therefore, we crystallised, solved, and refined the structure of the B6C domain to 2.05 Å limiting resolution [Supplementary Fig. 4 and Table 1]. Although B6C is composed almost entirely of α-helices like B75KN-C, its overall structure is far more extended than originally suggested by AlphaFold prediction, spanning over 110 Å in its greatest dimension. By contrast, B75KN-C is far more compact at roughly 50 Å when only its α-helical bundle is considered. These distinct α-helical structures of the B6C and B75KN-C domains likely dictate unique interactions with LILRB3 within the context of full-length β-protein and its subsequent ability to trigger inhibitory signalling in neutrophils.

**Table 1:** X-ray diffraction data, structure solution, and refinement statistics for B6C^a^

PDB accession code	9MN2
Data collection	
Beamline	ALS-5.0.1
Space group	*P*2_1_2_1_2
Wavelength (Å)	0.9774
Cell dimensions	
a, b, c (Å)	61.73, 79.03, 81.19
Resolution (Å)	50.00-2.05 (2.12-2.05)
Wilson B-factor (Å^2^)	42.4
Completeness (%)	99.4 (95.1)
I / σI	23.5 (1.3)
R_pim_	0.027 (0.494)
CC1/2	0.998 (0.561)
Redundancy	12.5 (10.5)
Structure solution	
Figure of merit	0.26
Heavy atom sites	2
Refinement	
Resolution (Å)	49.14-2.05 (2.08-2.05)
Number of reflections^b^	47,037
*R*_work_/*R*_free_ (%)	24.3/27.4
Total atoms modelled	2558
Ordered solvent	70
Ramachandran plot	
Favoured/allowed (%)	100.0/0
Average B-factors (Å^2^)	60.0
R.M.S. deviations	
Bond lengths (Å)	0.002
Bond angles (°)	0.47

^a^Values in parentheses are for the highest-resolution shell.

^b^Test set consisted of 3715 (7.9%) reflections.

As multiple attempts at crystallising the LILRB3/B6C and LILRB3/B75KN-C complexes were unsuccessful, we used small-angle X-ray scattering (SAXS) as an alternative approach to provide structural insights into these interactions. Both samples behaved as single, symmetrical peaks in SEC, indicating that they lacked aggregation. After routine data analysis procedures [Supplementary Fig. 5A–F] and calculation of the pair-distribution function (P(r)) for each complex [Supplementary Fig. 5C, D], we obtained maximal dimensions (Dmax) of 135 Å and 141 Å for rB6C/rLILRB3 and rB75KN-C/rLILRB3, respectively. We then generated ab initio density envelopes, which were consistent with the overall shapes of the computational models obtained for each complex [Supplementary Fig. 5G, H]. The theoretical SAXS profiles calculated from the rB6C/rLILRB3 and rB75KN-C/rLILRB3 models were in excellent agreement with the experimental SAXS data, as judged by the *χ*^2^ values of 0.92 and 1.02, respectively. Additional parameters from analysis and modelling of the SAXS data can be found in Supplementary Data 1.

**Fig. 5:**
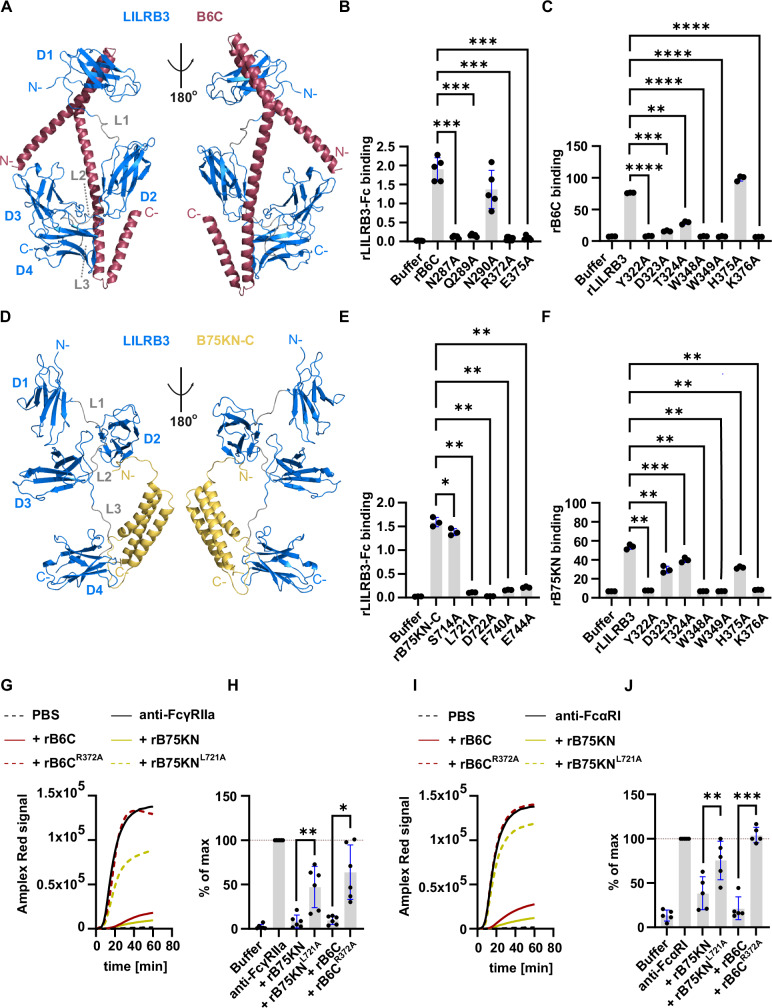
Structural and functional basis of β protein and LILRB3 interaction. **A** SAXS-derived structural model of the B6C (red) & LILRB3 (blue) complex. **B** Binding of rLILRB3-Fc to coated-rB6C wild-type and variants, quantified by ELISA. Mean ± s.d. of *n* = 5 independent experiments, with significance tested between rB6C wild-type and variants by one-way ANOVA using Dunnett’s post-hoc test, where B6C^N287A^ ***p = 0.0008, B6C^Q289A^ ***p = 0.0006, B6C^R372A^ ***p = 0.0006, B6C^E375A^ ***p = 0.0004. **C** Binding of rLILRB3-His wild-type and variants to coated-rB6C, quantified by flow cytometry. Mean ± s.d. of *n* = 3 independent experiments, with significance tested between rLILRB3-His wild-type and variants by one-way ANOVA using Dunnett’s post-hoc test, LILRB3^Y322A^ ****p < 0.0001, LILRB3^D323A^ ***p = 0.0001, LILRB3^T324A^ **p = 0.0027, LILRB3^W348A^ ****p < 0.0001, LILRB3^W349A^ ****p < 0.0001, LILRB3^K376A^ ****p < 0.0001. **D** SAXS-derived structural model of the B75KN-C (yellow) & LILRB3 (blue) complex. **E** Binding of rLILRB3-Fc to coated-rB75KN wild-type and variants, quantified by ELISA. Mean ± s.d. of *n* = 3 independent experiments, with significance tested between rB75KN wild-type and variants by one-way ANOVA using Dunnett’s post-hoc test, where B75KN^S714A^ *p = 0.0127, B75KN^L721A^ **p = 0.0041, B75KN^D722A^ **p = 0.0036, B75KN^F740A^ **p = 0.0050, B75KN^E744A^ **p = 0.0050. **F** Binding of rLILRB3-His wild-type and variants to coated-rB75KN, quantified by flow cytometry. Mean ± s.d. of *n* = 3 independent experiments, with significance tested between rLILRB3-His wild-type and variants by one-way ANOVA using Dunnett’s post-hoc test, where LILRB3^Y322A^ **p = 0.0031, LILRB3^D323A^ **p = 0.0028, LILRB3^T324A^ ***p = 0.0007, LILRB3^W348A^ **p = 0.0028, LILRB3^W349A^ **p = 0.0028, LILRB3^H375A^ **p< 0.0041, LILRB3^K376A^ **p = 0.0027. **G**–**J** FcR-mediated respiratory burst in human neutrophils, quantified using Amplex Red. In **G**, **I**, a representative Amplex Red plot is shown for FcγRIIa- and FcαRI-mediated respiratory burst. In **H**, **J**, % of the maximum Amplex Red signal at 60 min was calculated by normalising signals to anti-FcγRIIa or anti-FcαRI stimulated neutrophils. In **H**, mean ± s.d. from *n* = *6* independent experiments, with a comparison made between rB6C vs. rB6C^R372A^ (*p = 0.0125) and rB75KN vs. rB75KN^L721A^ (**p = 0.0059) by two-sided *t*-tests. In **J**, mean ± s.d. from *n* = 5 inde*p*endent experiments, wi*t*h a comparison made between rB6C vs. rB6C^R372A^ (***p = 0.0005) and rB75KN vs. rB75KN^L721A^ (**p = 0.0088) by two-sided *t*-tests.

In the B6C/LILRB3 complex model, the interface was formed primarily by the C-terminal region of the second α-helix of B6C and L3 and D4 of LILRB3 [Fig. 5A], consistent with our earlier biochemical analysis [Fig. 4B, F]. Upon inspection of the model, we identified residues K368, R372, E375 and K382 of B6C as potentially important sites for interaction with L3-D4 in LILRB3 [Supplementary Fig. 6A]. We expressed and purified mutant rB6C molecules with alanine substitutions at these and other residues [Supplementary Fig. 6B], which bound equally well to 96-well ELISA microtitre plates [Supplementary Fig. 6C]. The ELISA assays revealed that mutation at residues R372 or E375 impaired the binding of rLILRB3, whilst mutation at residue K382 also reduced rLILRB3 binding [Fig. 5B and Supplementary Fig. 6D]. The involvement of these residues at the interface was further confirmed by SPR analysis [Supplementary Fig. 6E, F and Supplementary Table 3], with mutated rB6C molecules with reduced rLILRB3 interaction displaying similar folding structure to wildtype rB6C in circular dichroism analysis [Supplementary Fig. 6G], indicating that loss of interaction was not due to misfolding of alanine-mutated proteins. We identified residues Y322, D323, T324, W348, W349, H375 and K376 in LILRB3 L3-D4 as potentially important sites for interaction with B6C. To assess their contribution, we expressed and purified mutant rLILRB3 molecules with alanine substitutions at these residues [Supplementary Fig. 7A]. The mutation of single residues in the loop regions of the LILRB3 Ig-domains [Supplementary Fig. 7B] to alanine did not lead to global unfolding of the LILRB3 protein as assessed using circular dichroism [Supplementary Fig. 7C]. However, the spectra of mutant LILRB3 had a more negative signal observed at ~215–218 nm, suggesting there could be an increase in β-strand content in the mutants. Mutation of LILRB3 at residues Y322, W348, W349 or K376 significantly reduced the interaction with B6C [Fig. 5C], whilst mutation of residues D323 or T324 reduced binding to rB6C. Of note, the mutation at the residue H375 in LILRB3, which had variation in the circular dichroism spectra compared to LILRB3, did not have any impact on interaction with B6C [Fig. 5C]. Though our previous results indicated that B6C does not bind to LILRB3 containing D1 but lacking D4 [Fig. 4B], the B6C/LILRB3 complex model suggested that residues N287, Q289, N290 and K294 of B6C may also be in close proximity to D1 of LILRB3 [Supplementary Fig. 6A]. Consistent with this, mutation of N287 or Q289 significantly reduced interaction with rLILRB3 [Fig. 5B, Supplementary Fig. 6D]. Overall, these observations show that the B6C domain targets residues in L3 (Y322) and D4 (W348, W349 and K376) of LILRB3 and that additional residues in D1 likely stabilise the interaction.

In the B75KN-C/LILRB3 complex model, the interface was formed between the first and second α-helices of B75KN-C and L3-D4 of LILRB3 [Fig. 5D]. This was also consistent with our earlier biochemical analysis that this region of LILRB3 was required for interaction with B75KN-C [Fig. 4C, G]. Following inspection of this model, we identified and mutated residues in B75KN-C as potential contact sites for L3 and/or D4 in LILRB3 [Supplementary Fig. 8A]. We expressed and purified mutant rB75KN molecules with alanine substitutions at these and other residues [Supplementary Fig. 8B], which bound equally well to 96-well ELISA microtitre plates [Supplementary Fig. 8C]. The ELISA assays revealed that mutation at residues L721, D722, F740 or E744 significantly reduced the binding of rLILRB3-Fc to coated rB75KN [Fig. 5E & Supplementary Fig. 8D]. The involvement of L721, D722, or F740 was confirmed by SPR analysis [Supplementary Fig. 8E, F and Supplementary Table 3]. The mutated rB75KN-C molecules with reduced LILRB3 interaction displayed a similar folding structure to wild-type rB75KN-C using circular dichroism [Supplementary Fig. 8G], confirming that loss of interaction was not due to misfolding upon alanine mutation. Notably, the same residues of L3 and D4 in LILRB3 that were identified as interaction sites for B6C were also identified as potential contact sites for B75KN-C. Mutation of rLILRB3 at residues Y322, W348, W349 or K376 significantly reduced the interaction with rB75KN-C [Fig. 5F], whilst mutation of residues D323, T324 and H375 reduced interaction affinity for rB75KN-C. Overall, these observations show that the B75KN-C domain targets residues in L3 (Y322) and D4 (W348, W349 and K376) of LILRB3. A map of amino acids in LILRB3 that are predicted to be contacted by B6C and B75KN-C based on structural models is shown in Supplementary Fig. 9.

To directly link these structural and biochemical findings to the observed functional effects of β protein on LILRB3 signalling, we assessed the ability of mutated B6C and B75KN variants to induce LILRB3 cross-linking. We found that single residue mutations in rB6C (R372A) and rB75KN (L721A) that disrupted LILRB3 binding also diminished cross-linking of LILRB3-CD3ζ reporter cells [Supplementary Fig. 10A, B]. Correspondingly, we found that mutation of rB6C at R372 and rB75KN at L721 reduced inhibition of FcγRIIa-dependent [Fig. 5G, H] and FcαRI-dependent [Fig. 5I, J] respiratory burst by neutrophils. Overall, these results confirmed that the interaction interfaces we characterised through solution structural methods are functionally relevant to the observed immunomodulatory effects of β protein.

### A single β protein molecule can dimerise LILRB3 for cross-linking

**Fig. 6:**
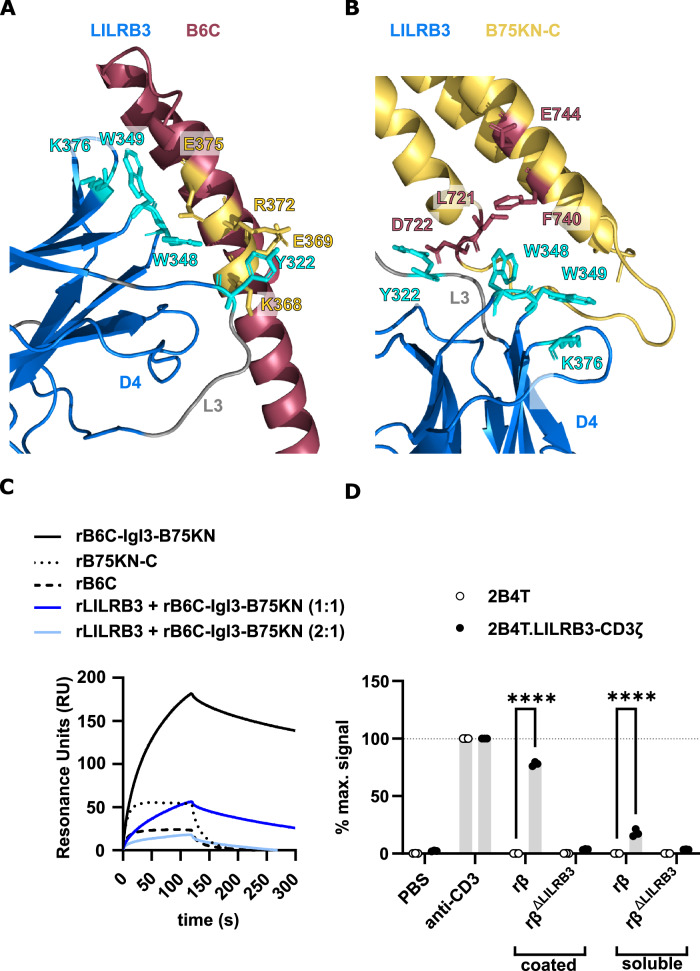
β protein dimerises LILRB3 for cross-linking. **A** A close-up view of the B6C & LILRB3 interface, with critical B6C (raspberry) residues highlighted in yellow and critical LILRB3 (blue, grey) residues highlighted in cyan. **B** A close-up view of the B75KN-C & LILRB3 interface, with critical B75KN-C (yellow) residues highlighted in raspberry and critical LILRB3 (blue, grey) residues highlighted in cyan. **C** Binding of rB6C-IgI3-B75KN to immobilised rLILRB3, quantified by SPR. rB6C-IgI3-B75KN was mixed with rLILRB3 at the indicated stoichiometric ratios. rB6C and rB75KN-C were included as controls. **D** Stimulation of GFP production in 2B4T reporter cells by rβ or rβ^ΔLILRB3^, quantified using flow cytometry. The percentage of GFP-positive cells was calculated and normalised against cells stimulated with anti-CD3. Mean ± s.d. of *n* = 3 independent experiments is shown, with two-way ANOVA with Šídáks multiple comparison test, where coated β protein 2B4T vs. 2B4T.LILRB3-CD3ζ ****p < 0.0001 and soluble β protein 2B4T vs. 2B4T.LILRB3-CD3ζ ****p < 0.0001.

Since our biochemical and structural analyses indicated that both the B6C and B75KN domains target the same residues in LILRB3 [Fig. 6A, B], we considered that a single β protein molecule could dimerise and cross-link two separate LILRB3 receptors. Such cross-linking could significantly amplify the inhibitory signal delivered to neutrophils. To investigate this possibility, we performed a competition assay designed to test the ability of rLILRB3-His to inhibit binding of rB6C-IgI3-B75KN-His (a protein containing the B6C domain and B75KN domain and the IgI3 domain sandwiched between as in the native protein, see Supplementary Fig. 2A) to captured rLILRB3-biotin. As expected, preincubation with rLILRB3-His inhibited binding of rB6C-IgI3-B75KN-His to the captured rLILRB3-biotin at a stoichiometric ratio of 1:1 [Fig. 6C], indicative of competitive inhibition. Notably, rB6C-IgI3-B75KN-His displayed further reduced binding to captured LILRB3 following preincubation at a stoichiometric ratio of 2:1. This strongly suggested that a second rLILRB3-His molecule interacted with the second binding site on rB6C-IgI3-B75KN-His, leading to the diminished binding of immobilised LILRB3. Molecular weight estimates obtained from SAXS analysis were also in agreement with this interpretation. Whereas B6C & LILRB3 (87 kDa) and B75KN-C & LILRB3 (78 kDa) formed complexes consistent with 1:1 interactions, a sample of rB6C-IgI3-B75KN-His with two equivalents of rLILRB3-His formed a complex consistent with a 1:2 interaction (177 kDa), as judged by the volume of correlation statistic [Supplementary Data 1].

To test LILRB3 cross-linking by a single β protein molecule in a physiological context, we used our LILRB3-CD3ζ reporter cells. We reasoned that soluble rβ, if capable of cross-linking, should stimulate GFP expression in these cells. Therefore, we purified rβ and rβ^ΔLILRB3^ (rβ with mutations B6C^K368A^, B6C^R372A^, B75KN^L721A^, B75KN^D722A^ and B75KN^F740A^ to abolish LILRB3 interaction) from *E. coli* [Supplementary Fig. 11A]. The wildtype and mutated proteins had almost superimposable circular dichroism spectra, indicating rβ^ΔLILRB3^ folded into a native-like rβ conformation [Supplementary Fig. 11B]. Both surface-coated (on the microtitre well) and soluble rβ, but not surface-coated (on the microtitre well) and soluble rβ^ΔLILRB3^, stimulated GFP expression in LILRB3-CD3ζ reporter cells but not control cells [Fig. 6D]. It is notable that GFP expression was lower when LILRB3-CD3ζ cells were stimulated with soluble compared to coated rβ, likely indicating that β is more efficient at cross-linking LILRB3 when it is anchored to the bacterial surface. Collectively, these findings reveal a mechanism of LILRB3 cross-linking, whereby a single bacterial protein molecule acts as a dimerising scaffold to bring two inhibitory receptor molecules into close proximity, potentially amplifying inhibitory signalling and driving immunosuppressive phenotypes.

### LILRB3 targeting plays an important role in protecting *S. agalactiae* from antibody- and FcR-dependent killing by neutrophils

**Fig. 7:**
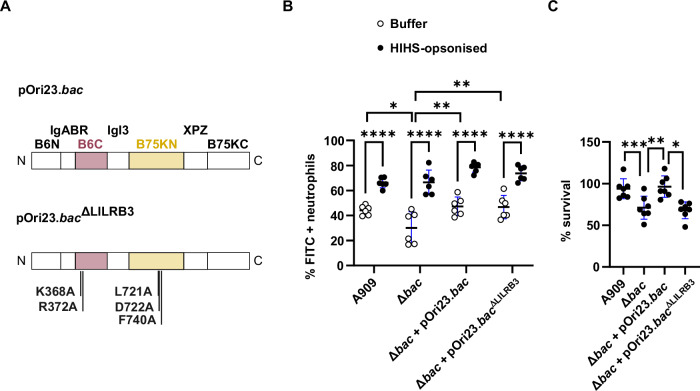
β protein targeting of LILRB3 impairs killing of *S. agalactiae* by neutrophils. **A** Schematic of pOri23 vectors, showing β protein domains (B6N, IgABR, B6C, IgI3, B75KN, XPZ and B75KC) shown. pOri23.*bac* encoding wildtype β protein or pOri23.*bac*^ΔLILRB3^ encoding β protein with mutations in B6C (K368A and R372A) and B75KN (L721A, D722A, F740A). **B** Adhesion of FITC-labelled *S. agalactiae* strains to neutrophils, quantified using flow cytometry. FITC-labelled strains were incubated in buffer or 10% heat-inactivated human serum (HIHS) and mixed with human neutrophils at an MOI of 10. Data reported as % of FITC+ neutrophils. Mean ± s.d. from *n* = 3 independent experiments is shown, with one-way ANOVA, where comparison of buffer vs. HIHS for each strain ****p < 0.0001, A909/buffer vs. A909Δ*bac*/buffer *p = 0.0194, A909Δ*bac*/buffer vs. A909Δ*bac+*pOri23*.bac*/buffer **p = 0.0027, A909Δ*bac*/buffer vs. A909Δ*bac+*pOri23*.bac*Δ^LILRB3^/buffer **p = 0.0038. **C** Survival of *S. agalactiae* strains after co-incubation with human neutrophils. Strains were opsonised with 10% HIHS and mixed with human neutrophils at an MOI of 10. The percentage of *S. agalactiae* surviving from the inoculum was determined. Mean ± s.d. from *n* = 7 independent experiments is shown, with one-way ANOVA, where A909 vs. A909Δ*bac* ***p = 0.0009, A909Δ*bac* vs. A909Δ*bac +* pOri23.*bac* **p = 0.0033 and A909Δ*bac +* pOri23.*bac* vs. A909Δ*bac +* pOri23.*bac*Δ^LILRB3^ *p = 0.0125.

Defining the β-LILRB3 binding sites allowed for functional assessment of this interaction on cellular behaviours and antibacterial immune responses. This is important because β protein also binds Siglec-5 and CEACAM1 expressed on neutrophils^41,44^. To isolate the contribution of the LILRB3 interaction in cellular adhesion, we generated two expression vectors that encoded a β protein that would be anchored onto the bacterial cell wall [Fig. 7A]. Whilst the first vector encoded wild-type β protein, the second vector encoded a β protein variant with mutations as described for rβ^ΔLILRB3^. Importantly, the total and surface β protein expression was comparable between both β-expressing *L. lactis* strains [Supplementary Fig. 12A, B]. Further, rCEACAM1 and rSiglec-5 still bound to *L. lactis* expressing β^ΔLILRB3^ at a level comparable to β-expressing *L. lactis* [Supplementary Fig. 12C]. In contrast, rLILRB3 bound to *L. lactis* expressing wild-type β protein but not *L. lactis* expressing β^ΔLILRB3^ [Supplementary Fig. 12C]. Furthermore, *L. lactis* expressing β, but not *L. lactis* expressing β^ΔLILRB3^, displayed enhanced adhesion to LILRB3-expressing U937 cells relative to U937 cells [Supplementary Fig. 12D]. Given this, we complemented the *S. agalactiae* Δ*bac* strain with pOri23.*bac* or pOri23.*bac*^ΔLILRB3^ vectors. The Δ*bac* strain carrying pOri23.*bac*^ΔLILRB3^ had a higher expression level of surface β protein [Supplementary Fig. 12E]. However, there was no significant difference in their interaction with CEACAM1 or Siglec-5 [Supplementary Fig. 12F]. In contrast, they differed in LILRB3 interaction. Collectively, this confirmed that the β^ΔLILRB3^ variant retained its structural integrity and the ability to interact with other human receptors but did not interact with LILRB3.

Having delineated the β-LILRB3 interaction from the other known interactions of β protein with human neutrophil receptors, we next investigated how this specific interaction impacted the interaction of *S. agalactiae* with human neutrophils and antibody- and FcγR-dependent killing. Firstly, to ascertain how the β-LILRB3 interaction impacted bacteria-to-cell interactions, we incubated neutrophils at 4 °C (to prevent phagocytosis) with FITC-labelled *S. agalactiae* and measured the % of fluorescent neutrophils. *S. agalactiae* binding to neutrophils depended on β protein expression, as the interaction with A909 was significantly diminished in the Δ*bac* strain [Fig. 7B]. Notably, complementation of the Δ*bac* strain with the β-expressing vector or β^ΔLILRB3^-expressing vector restored neutrophil adhesion [Fig. 7B], indicating that β-LILRB3 interaction alone did not alter *S. agalactia*e adhesion to human neutrophils. This is likely attributable to the interaction of β^ΔLILRB3^ with factor H, Siglec-5, Siglec-14 and/or CEACAM1 on the neutrophil surface^41,44^. Opsonisation of the FITC-labelled *S. agalactiae* strains with 10% heat-inactivated human sera, as a source of antibodies but not complement, enhanced adhesion of all strains to human neutrophils [Fig. 7B]. Importantly, the strains did not display a significant difference in adhesion to human neutrophils when opsonised with human antibodies.

Lastly, the relative contributions of targeting LILRB3 in evasion of antibody- and FcγR-dependent killing by neutrophils were examined. To do this, we compared the survival of *S. agalactiae* strains that were opsonised with 10% heat-inactivated human serum. The deletion of *bac* in the *S. agalactia*e A909 strain significantly reduced survival in the neutrophil killing assays, confirming previous findings^44^, whilst complementation with the pOri23 vector carrying the *bac* gene re-established bacterial survival [Fig. 7C]. However, complementation with the pOri23 vector carrying the *bac*^ΔLILRB3^ gene did not restore *S. agalactiae* survival [Fig. 7C]. These results demonstrate that the β-LILRB3 interaction is a key mechanism for *S. agalactiae* to evade antibody-dependent killing by neutrophils.

### Human LILRB3 is targeted by β protein-expressing *S. agalactiae* lineages associated with adult invasive disease

**Fig. 8:**
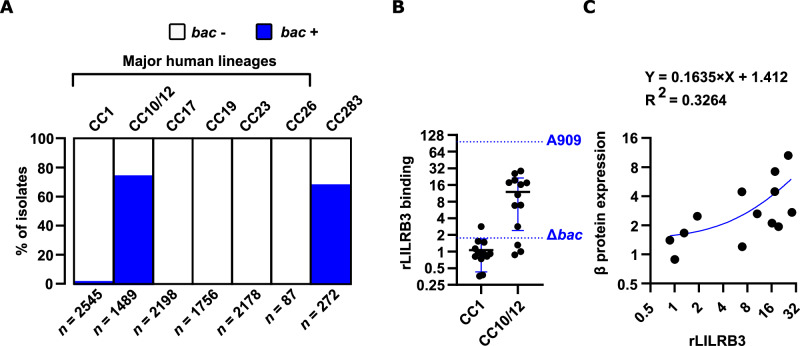
β protein + *S. agalactiae* target human LILRB3 and are associated with clonal complex lineages causing disease in non-pregnant adults. **A** *bac* gene presence in *S. agalactiae* isolates from the human clonal complex (CC) lineages, where CC1, CC10/12, CC17, CC19, CC23 and CC26 are major human lineages. The number of isolates assessed in each CC is shown. **B** Binding of rLILRB3 to *S. agalactiae* strains, quantified using ELISA. Each data point represents one strain (*n* = 11 CC1 strains, *n* = 14 CC10/12 strains), calculated from the mean of *n* = 2 independent experiments. Dashed lines indicate signal from a β-positive (A909) and -negative (A909Δ*bac*) control strain. Data are displayed as relative rLILRB3-binding compared to control. Mean ± s.d. is shown. **C** Correlation between β protein expression and rLILRB3-binding capacity of clinical *S. agalactiae* isolates from lineage CC10/12, using values in Fig. 8B. Each data point represents one strain.

An emergent feature of *S. agalactiae* pathogenicity is that certain CC lineages have a greater potential to cause disease in certain demographics^47^, illustrated by the common isolation of CC17 in neonates and CC1 in non-pregnant adults with co-morbidities^47–51^. Based on the demonstrated role that LILRB3-targeting promotes evasion of antibody-dependent killing, we hypothesised that the *bac* gene would be prevalent in *S. agalactiae* lineages that cause disease in individuals with antibody responses that are waning. In these hosts, antibody titres and responses may fall within a critical window, where they can be overcome by *S. agalactiae* triggering LILRB3-mediated inhibition; conversely, robust antibody titres and responses in immunocompetent individuals may simply be too efficient for LILRB3-targeting to overcome IgG- and Fc-receptor-dependent responses of neutrophils. Therefore, we anticipated that *bac* would be present in lineages associated with invasive disease in adults, but absent in lineages associated with neonatal disease. Given that *S. agalactiae* lineages or clones carrying the *bac* gene have remained undefined, we analysed a PubMLST database of 11,744 *S. agalactiae* genomes for carriage of *bac* across different clonal complex (CC) lineages. Of the 6 major human CC lineages, a high proportion of isolates from lineages CC10/12 (75.3% *bac* positive), and a small proportion of isolates from CC1 (2.8% *bac* positive), carried *bac* [Fig. 8A]. In contrast, no isolates from the other major human lineages of CC17, CC19, CC23 or CC26 carried the *bac* gene^52^. In addition, a high proportion of isolates from the minor human lineage CC283 (68.4% *bac* positive) carried *bac*. Other minor (CC327, CC452 and CC459) human lineages were *bac*-negative. Therefore, *bac* emerged in at least 3 independent *S. agalactiae* lineages (CC1, CC10/12 and CC283), all of which are increasingly associated with invasive infections in non-pregnant adults^51,53–57^. In contrast, the CC17 and CC19 lineages displaying hypervirulence in neonates were *bac*-negative. This distribution establishes an epidemiological link between the *bac* gene and *S. agalactiae* lineages that cause invasive disease in the adult population.

As it was important to test the relevance of LILRB3 targeting by *S. agalactiae* from these specific lineages, a panel of clinical isolates from CC10/12 that were previously genome sequenced as *bac*+ were screened for rLILRB3 binding and for surface β protein expression^58^. The analysis of rLILRB3 interaction revealed that 11/14 (79%) isolates from CC10/12, but none of the CC1 isolates, interacted with rLILRB3 [Fig. 8B]. Using a monoclonal antibody raised against the IgI3 domain of β protein, we revealed a correlation between surface β protein expression and rLILRB3 binding [Fig. 8C]. These data indicate that a major *S. agalactiae* lineage causing human invasive diseases, CC10/12, has acquired the capacity to express β protein and target LILRB3. We confirmed that β protein expression in clinical isolates is a functional ligand of LILRB3 using the LILRB3-CD3ζreporter cells [Supplementary Fig. 13].

Since *S. agalactiae* interacted with LILRB3 to inhibit FcγR-dependent neutrophil responses, we further investigated the species-specificity of host-pathogen interactions. As murine models are routinely used to study *S. agalactiae* infection and dissect complex functional interactions, we assessed the capacity of β protein-expressing *S. agalactiae* to interact with the murine orthologue of LILRB3, known as PIR-B. Of note, human LILRB3 and murine PIR-B do not share a high degree of structural conservation [Supplementary Fig. 14A], represented by the fact that the ectodomain of murine PIR-B possesses 6 Ig-like domains compared to 4 Ig-like domains of LILRB3. We found that rPIR-B did not bind to *S. agalactiae* A909 in flow cytometry analysis [Supplementary Fig. 14B]. Consistent with this, rLILRB3, but not rPIR-B, bound to immobilised β protein in ELISAs [Supplementary Fig. 14C]. These results demonstrate that *S. agalactiae* does not interact with murine PIR-B, highlighting that the interaction with human LILRB3 may be a species-specific immune evasion mechanism.

## Discussion

Bacterial pathogens have evolved mechanisms for evasion of antibody-dependent neutrophil responses^59,60^. Although human bacterial pathogens have been identified to interact with human inhibitory receptors, it has remained unclear whether these interactions allow pathogenic bacteria to overcome antibody- and FcR-driven immune responses. We now provide experimental evidence that *S. agalactiae* targets the inhibitory LILRB3 receptor to suppress antibody and FcR-dependent responses of primary human neutrophils. By presenting experimental evidence that *S. agalactiae* targets the inhibitory LILRB3 receptor to suppress antibody- and FcR-dependent responses of human neutrophils, we highlight that bacterial pathogens target inhibitory receptors to evade phagocyte-mediated adaptive antibacterial immunity.

**Fig. 9:**
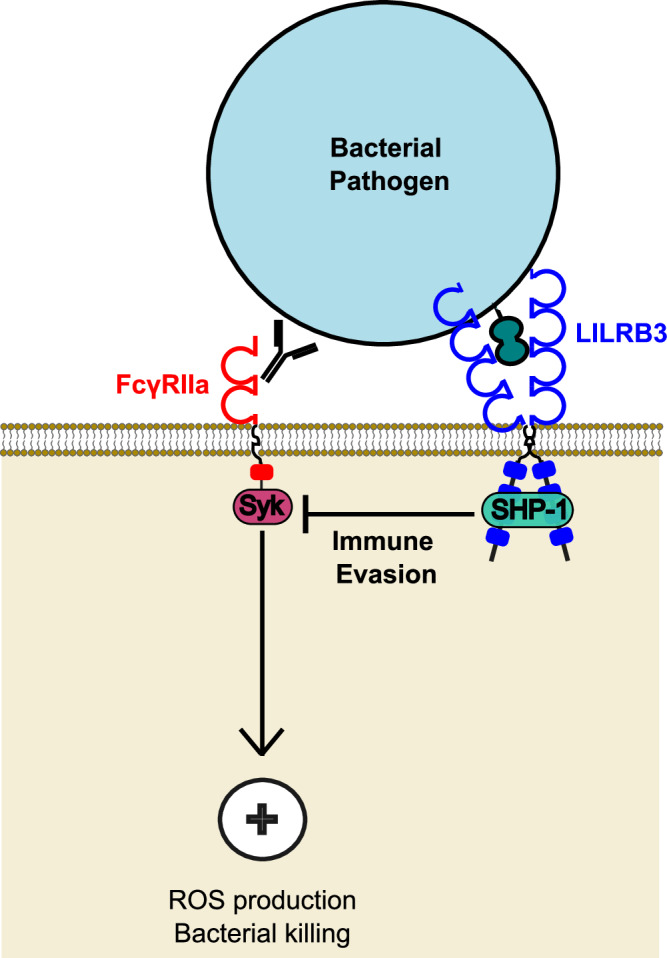
Proposed model of bacterial evasion of antibody-mediated responses by targeting inhibitory receptors. Invasion of hosts with acquired immunity can result in the opsonisation of the bacterial pathogen surface with antibodies. These antibody-opsonised bacteria can be recognised by Fc receptors (FcR, such as FcγRI, FcγRIIa, FcγRIIIb and FcαRI) on neutrophils. This recognition induces phosphorylation of immunoreceptor tyrosine-based activation motifs (ITAMs) of FcR and activation of the protein tyrosine kinase Syk that signals for the antibacterial immune response. This is typically composed of the production of reactive oxygen species (ROS), phagocytic uptake and the inflammatory response for immune cell recruitment. The binding of bacterial pathogens to inhibitory receptors (such as LILRB3) can induce cross-linking and phosphorylation of immunoreceptor tyrosine-based inhibition motifs (ITIMs) in the cytoplasmic tail. This likely leads to, though requires experimental validation, the recruitment of protein tyrosine-phosphatases (such as SHP-1, SHP-2 and/or SHIP) that can counteract phosphorylation of Syk and downstream signalling molecules. The targeting of an inhibitory receptor by an antibody-opsonised bacterial pathogen provides an immune evasion mechanism to dampen FcR-dependent antibacterial immune responses.

Neutrophils, the most abundant immune cell in the body, can destroy extracellular pathogenic bacteria. This frequently operates through an antibody-dependent mechanism, in which FcγRIIa (CD32A) or FcγRI (CD64) recognise the Fc portion of IgG antibodies bound to a pathogen^1^. Our data provide evidence that *S. agalactiae* targets the human inhibitory LILRB3 receptor to evade antibody- and FcR-driven neutrophil responses [Fig. 9]. First, the β protein of *S. agalactiae* binds to LILRB3 through two independent domains called B6C and B75KN. Second, these domains inhibit FcγRIIa- and FcαRI-mediated respiratory burst in neutrophils, and these phenotypes were reversed for variants mutated to ablate the LILRB3 interaction. Third, antibody-opsonised *S. agalactiae* that express wild-type β protein display enhanced survival during incubation with human neutrophils compared to antibody-opsonised *S. agalactiae* that express a form of β protein that cannot interact with LILRB3, suggesting that β protein can directly inhibit FcγR-driven bacterial killing by targeting LILRB3. These results suggest that β protein likely clusters LILRB3 on the neutrophil surface and consequently triggers the phosphorylation of the ITIMs in the cytoplasmic tail and recruitment of tyrosine phosphatases such as SHP-1 and SHP-2^42,61,62^. This would raise the threshold required for FcγRIIa or FcRγ associated with FcγRI^27^ to induce phagocytosis, ROS production, and bacterial killing [Fig. 9]. The phosphorylation of ITIMs and the recruitment of SHP-1 or SHP-2 to ITIMs in inhibitory receptors are often rapid and transient in nature. Whilst our current study is consistent with β protein inducing LILRB3 ITIM phosphorylation and subsequent signalling events to inhibit the FcγR-dependent activation and effector responses of neutrophils, future studies utilising a temporal analysis of LILRB3 phosphorylation and signalling events in primary neutrophils will be valuable to fully elucidate the kinetic window of inhibition induced by bacterial pathogens.

β protein promotes *S. agalactiae* virulence^45,63^ and is now known to specifically target 4 human inhibitory receptors: LILRB3, CEACAM1, Siglec-5 and Siglec-7^41,44,64^. Their targeting through distinct mechanisms emphasises the likely importance of inhibitory receptor targeting in bacterial immune evasion. The first identified inhibitory receptor targeted by the β protein, via the B6N domain, was Siglec-5^44^. Whilst the Siglec-5 interaction was reported to promote *S. agalactiae* survival from neutrophil-mediated killing^44^, interpretations of these data are not straightforward since it is unclear whether the anti-Siglec-5 mAb blocks Siglec-5/β protein interactions and/or influences β protein interactions with other inhibitory receptors. Additionally, the paired activating receptor Siglec-14 can also bind the B6N domain to overcome Siglec-5-induced responses^65^. The functional consequences of β protein interactions with CEACAM1 (via the IgI3 domain) and Siglec-7 (via the B6N domain) on neutrophil responses have not yet been examined. Our analyses reveal that the B6C and B75KN domains of the β protein bind LILRB3 by targeting the D3-L3-D4 region of the receptor. While our structural analysis and targeted mutagenesis identified specific amino acids critical for this interaction, we acknowledge that circular dichroism analysis of our LILRB3 mutants revealed potential alterations in secondary structure, potentially indicating an increase in β-strand content. Consequently, we cannot definitively rule out that the loss of interaction is strictly due to the removal of direct contact residues, as alternatively, there could be an indirect effect of local structural shifts that alter interface conformations. Future high-resolution structural studies will be highly valuable to definitively map the interfaces of these complexes.

Despite targeting multiple inhibitory receptors, our results here show that specifically abolishing the interaction of β protein with LILRB3 through targeted mutagenesis suppressed FcγR-mediated activation and IgG-mediated bacterial killing. The mutated versions of β, unable to interact with LILRB3, showed diminished but not entirely eliminated immune suppressive effects on neutrophil responses. This could be explained by the targeting of alternative inhibitory receptors (such as CEACAM1, Siglec-5 or Siglec-7) on neutrophils. These findings highlight the importance of further investigating the individual and synergistic contributions of inhibitory receptor interactions in subverting antibody- and FcγR-dependent responses and in shaping the outcome of infection. As the expression of inhibitory receptors by immune cells is regulated at both cellular and spatiotemporal levels^27^, it is likely that β protein targets multiple inhibitory receptors to ensure efficient suppression of the multiple cell types that it may encounter during infection. For example, LILRB3 is a suitable target on circulating neutrophils as it is highly expressed and co-expressed with FcγR. In contrast, CEACAM1 and LILRB2 would be suitable targets on activated neutrophils, since these inhibitory receptors have low surface expression levels on neutrophils until they become activated and exocytose granules that deliver these inhibitory receptors to the cell surface^20,66^. Likewise, Siglec-7 might be an excellent target on NK cells due to its high expression. Thus, bacterial pathogens may selectively engage specific inhibitory receptors to overcome FcR responses at different stages of infection, reflecting the host cell populations and activation profiles that drive immunity during the dynamic infection process. In this regard, β protein provides an effective multilayered system for understanding how targeting of distinct inhibitory receptors shapes immune evasion by bacterial pathogens.

Based on these findings, we propose that this mechanism of evasion may apply to other inhibitory receptors and bacterial pathogens. The expression of various inhibitory receptors on phagocyte surfaces, with LILRB3 being an example on circulating neutrophils^18^, likely creates a powerful evolutionary pressure for bacteria to develop immune evasion mechanisms. By binding to these inhibitory receptors, bacterial pathogens can effectively raise activation thresholds for FcR-mediated cellular activation, phagocytosis and killing. We note that a previous study identified that *S. aureus* and *E. coli* displayed enhanced adhesion to cells overexpressing LILRB3^37^, though the bacterial ligands of LILRB3 and the functional consequences of these interactions remain to be defined. Nonetheless, our definition of a bacterial ligand of LILRB3 with functional studies, combined with reports of other bacteria-LILRB3 interactions, suggests that multiple bacterial species have evolved distinct ligands to target LILRB3 on neutrophils. The mechanism of immune evasion is likely not restricted to a single inhibitory receptor, as other inhibitory receptors expressed by neutrophils are targeted by bacterial pathogens. For example, CEACAM1, CLEC12A, Siglec-5 and SIRL-1 are targets of bacterial factors^26,41,44,67–71^. Bacterial pathogens also likely use this strategy across different phagocytic immune cells, such as macrophages, in which the highly expressed inhibitory receptors could be targeted. Thus, we propose that multiple bacterial pathogens have evolved mechanisms to target inhibitory receptors to overcome the induction of antibody- and FcR-dependent phagocyte responses.

Uniquely, the β protein has evolved to possess a scaffold with two LILRB3-binding domains, enabling it to dimerise two LILRB3 molecules. Thus, *S. agalactiae* has evolved a dedicated and highly efficient mechanism to activate LILRB3, thereby maximising the suppression of host cell activity. This mechanism sets a precedent that pathogen-derived molecules can possess dimeric or multimeric scaffolds to ensure maximal activation of host inhibitory receptors and to maximise immune evasion.

Understanding how host-pathogen interactions contribute to bacterial disease requires investigation in complex in vivo systems. The capacity of β-LILRB3 interactions to promote *S. agalactiae* virulence would be best tested in mice that have acquired anti-*S. agalactiae* immunity following sub-lethal exposure. However, neither *S. agalactiae* nor the isolated β protein interacted with the PIR-B receptor, the murine orthologue to human LILRB3. This is not surprising given the differences in expression profiles, structures and ligands of PIR-B and LILRB3^72,73^. This also highlights that transgenic human *LILRB3* mice are required to investigate the functions of targeting inhibitory receptors in *S. agalactiae* invasive disease, and that additional studies are required to fully define the host-specificity of β-LILRB3 interactions.

Molecular epidemiological analysis of invasive *S. agalactiae* isolates has revealed that the *bac* gene encoding β protein emerged in the CC10/12 and CC283, and to a lesser extent, CC1, lineages. Whilst lineages CC17 and CC19 have a greater potential to cause disease in neonates and infants^47^, which is associated with expression of the HvgA adhesin^74^, CC10/12 and CC283 have become increasingly associated with invasive diseases in non-pregnant adults^51,53–57^. The association of *bac* to lineages that have a greater potential to cause disease in non-pregnant adults, but not neonates, is intriguing and suggests that *bac* may provide an advantage to the bacterium in cases where antibody immunity in the host is present. Ageing is known to influence lymphocyte development and neutrophil function^75,76^. Indeed, neutrophils from the elderly have a modified capacity for chemotaxis^77,78^, phagocytosis^79,80^, respiratory burst^77^ and microbial killing^4,80^. This suggests that targeting an inhibitory receptor may play a critical role in the pathogenesis of diseases where natural immune functionality is waning. We therefore postulate a model where there is a ‘window of opportunity’ for *S. agalactiae* to overcome the antibody response. In healthy adults, the antibody response will be too efficient to be overcome by LILRB3-mediated evasion, whereas in neonates that lack an antibody response, this immune evasion mechanism is redundant. However, in hosts with low antibody titres or waning antibody responses, LILRB3-targeting is efficient at overcoming the antibody response and promoting *S. agalactiae* survival. As such, the dampening of antibody- and FcγR-dependent neutrophil activation by targeting an inhibitory receptor may allow for a bacterial pathogen to survive longer in the host and to cause more severe infections. It is noteworthy that *S. agalactiae*-specific antibodies are detected in healthy elderly individuals but often at low titre^81^, and that the antibodies in serum of elderly individuals are not effective at promoting neutrophils to phagocytose and kill *S. agalactiae*^4^. Indeed, it could also be anticipated that other *S. agalactiae* lineages, which are *bac*-negative yet have a greater potential to cause disease in non-pregnant adults, such as CC1 or CC23, may have alternative strategies to target inhibitory receptors expressed on phagocytes. It is also possible that lineages, such as CC17 and CC19, with a heightened propensity to cause disease in neonatal environments, target inhibitory receptors that are relevant to the developing immune system.

In summary, our study has identified that *S. agalactiae*, via the surface-expressed β protein, targets the inhibitory LILRB3 receptor to suppress antibody- and FcR-mediated activation of neutrophils. This interaction reduces antibody-dependent activation and impairs bacterial killing. These findings expand our knowledge of the functions of inhibitory receptor targeting in bacterial immune evasion. Inhibitory receptor targeting by bacteria has been shown to dampen innate PRR-dependent immune responses^82,83^, whilst here we show these interactions can dampen adaptive antibody-dependent immune responses. As a growing list of human inhibitory receptors beyond LILRB3 are known to be targeted by bacterial pathogens^26,41,44,67^, manipulating inhibitory receptors to disrupt antibody- and FcR-dependent neutrophil responses likely represents a common strategy of bacterial immune evasion.

## Methods

### Ethics

The collection of human blood from healthy donors was approved by the Regional Ethics Committee and Imperial College Healthcare NHS Trust Tissue Bank (Regional Ethics Committee approval no. 17/WA/0161, Imperial College Healthcare Tissue Bank Human Tissue Authority license no. 12275, and Imperial College Research Ethics Committee no. 19IC5166). Signed informed consent from all participants was received prior to the collection of samples.

### Human blood and serum

Human neutrophils were isolated by Ficoll/Histopaque centrifugation from human blood samples, as previously described^18^, and resuspended in RPMI 1640 supplemented with 2% fetal calf serum (FCS). Pooled human serum (from a minimum of 4 donors) was prepared from blood collected from healthy human donors using BD Vacutainer Tube Sst Advance Gold Blood Collection Tubes, following the manufacturer's protocols. Data reported relates to both sexes and all genders. Sex and gender were not considered in the study design as the primary objective was to characterise the fundamental molecular mechanisms of immune cell activation and bactericidal activity against *S. agalactiae*. The study was not statistically powered to detect differences between sex or gender groups.

### Measurement of surface expression of Fc-receptors and LILRB3 on human neutrophils

Human neutrophils were isolated from peripheral blood following a standard Ficoll/Histopaque density gradient centrifugation protocol, as described and resuspended in PBS with 0.5% BSA. Fifty thousand cells per condition were placed on ice and stained in a three-step stain, starting with mouse anti-human LILRB3 (R&D #222821) or isotype control IgG2a (Millipore #PP102) at 10 μg/ml, followed by anti-mouse-IgG-AF647 (Jackson ImmunoResearch #115-606-146) at 1:500, and finally with anti-human FcγRIIIb/CD16-PE (BD Biosciences #555407), anti-human FcγRIIa/CD32-PE (ABD serotec #AT10-PE), anti-human FcγRI/CD64-PE (Fitzgerald, clone 10.1), or anti-human FcαRI/CD89-PE (BioRad #MCA1824PE) all at 1:200. Cells were analysed using BD FACS Calibur.

### Bacterial strains

Bacterial strains used in this study are listed in Supplementary Data 2. *S. agalactiae* were cultured in Todd-Hewitt Broth (TH) at 37 °C with 5% CO_2_, *E. coli* were cultured in Lysogeny Broth (LB) at 37 °C, and *L. lactis* were cultured in M17 media supplemented with 0.5% (w/v) glucose and 0.5% (w/v) lactose (GM17) at 30 °C^41,46^. The medium was supplemented with antibiotics when required as follows; *S. agalactiae* selection with 5 µg/ml erythromycin; *E. coli* selection with 100 µg/ml ampicllin or 500 µg/ml erythromycin; *L. lactis* selection with 5 µg/ml erythromycin.

The original wild-type pOri23.*bac* vector for expression of β protein in *L. lactis* and *S. agalactiae* was previously reported^46^. The wild-type pOri23.*bac* vector was used as the template to construct (i) pOri23.*bac*∆^LILRB3^, a vector encoding β protein with amino acid substitutions in the B6C (K368 and R372A) and B75KN (L721A, D722A, F740A), and (ii) pOri23.*bac*, a vector encoding wildtype β protein that was cloned in the same manner as pOri23.*bac*∆^LILRB3^. To construct these vectors, the original wild-type pOri23.*bac* from Xu et al.^46^ was used as a template for inverse PCR in which the B6C-IgI3-B75KN domains of β protein were cut out. The forward primer (^5’^GATCCTCGAGACGCCAGAAACTCCAGAT^3’^) contained an *Xho*I restriction site, and the reverse primer (^5’^GATCCGCGGCCGCATCCAGACCAGCTTTAGTTG^3’^) contained a *Not*I restriction site. PCR amplification was performed using Phusion High-Fidelity Taq Polymerase (Thermo Fisher Scientific) and thermocycling as follows:- 1 cycle (98 °C for 2 minutes), 25 cycles (98 °C 15 s, 66 °C 30 s and 72 °C 5 min), and 1 cycle (72 °C 10 min). Amplicons were digested with *Dpn*I and then digested with *Not*I and *Xho*I. Next, synthetic DNA fragments encoding wildtype or mutated B6C-IgI3-B75KN domains [Supplementary Data 3] were subcloned into the *Not*I and *Xho*I sites of the purified amplicon by Gibson Assembly reaction following a standard protocol. Ligated vectors were transformed into *E. coli* DH5a and selected for through growth on LB agar plates containing 500 µg/mL erythromycin.

To produce β protein-expressing *L. lactis*, competent *L. lactis* were transformed with pOri23 vectors and selected for growth on GM17 agar plates supplemented with 5 μg/mL erythromycin^46^. To produce β protein-expressing *S. agalactiae*, competent *L. lactis* were transformed and selected for on GM17 agar plates supplemented with 5 μg/mL erythromycin.

### Cell lines and culture

U937, THP-1 and 2B4 NFAT-GFP T cell lines were cultured in RPMI 1640 + 10% FCS, penicillin-streptomycin at 37 °C with 5% CO_2_. The LILRB3-CD3ζ reporter 2B4 NFAT-GFP T (2B4T) cell line was previously reported^18^.

To construct a LILRB3-expressing U937 and THP-1 cell line, a DNA fragment containing the ectodomain of LILRB3 was amplified from a *LILRB3* cDNA vector (HG11978-M, BioConnect) using primers^5’^GAGCTAGCAGTATTAATTAACCACCATGACGCCCGCCCTCACAGCCCTG^3’^ and^5’^GTACCGGTTAGGATGCATGCCTAGTGGATGGCCAGAGTGGCGTAG^3’^. PCR amplification was performed using Phusion High-Fidelity Taq Polymerase (Thermo Fisher Scientific) and thermocycling as follows:- 1 cycle (98 °C for 2 min), 35 cycles (98 °C 15 s, 60 °C 30 s and 72 °C 30 s), and 1 cycle (72 °C 10 min). The purified amplicon was ligated into a dual promoter lentiviral vector (BIC-PGK-Zeo-T2a-mAmetrine); RP172 derived from no.2025.pCCLsin.PPT.pA.CTE.4⋅-scrT.eGFP.mCMV.hPGK.NG-FR.pre^18^, via Gibson reaction using a parental vector digested with *Pac*I and *Sph*I. Lentiviral particles were created in HEK293T cells and transfected into U937 cells^18^. Media was supplemented with 400 μg/mL zeocin to select for transformed cells.

### Expression and purification of rLILR

rLILRs with C-terminal His-tag or C-terminal Fc-tag were expressed in Expi293F cells (Life Technologies) cultured in Expi293 Expression Medium (Life Technologies) and purified by affinity chromatography (ÄKTA Pure, GE Healthcare Life Sciences) using a Nickel column (GE Healthcare Life Sciences), as previously described^18^. Proteins were stored in 50 mM Tris 300 mM NaCl (pH 8) at −80 °C.

The expression of rLILRB3-His, rLILB2-His and rCEACAM1-Fc has been previously described^18,26,41^. rSiglec5-Fc was supplied by R&D Biosystems. To construct expression vectors for expression of rLILRB3-Fc and rLILRB2-Fc, the signal peptide and extracellular domains of LILRs were amplified from cDNA vectors and inserted into a pcDNA3.4 vector that had previously had DNA encoding the CH2 and CH3 domains of human IgG inserted. The forward primers contained a pcDNA3.4 backbone overhang, a Kozak sequence, a *Bam*HI restriction site, and a gene-specific region. The reverse primers contained a sequence from the 5’ end of human CH2, a *Nde*I restriction site, and a gene-specific region. Primers and template vectors are listed in Supplementary Data 4. PCR amplification was performed using Phusion High-Fidelity Taq Polymerase (Thermo Fisher Scientific) and thermocycling as follows:- 1 cycle (98 °C for 2 min), 35 cycles (98 °C 15 s, 62 °C 30 s and 72 °C 30 s), and 1 cycle (72 °C 10 min). Amplicons were inserted into a parental vector pcDNA3.4-CH23 digested with *Bam*HI and *Nde*I via Gibson reaction following standard protocol^18^. Ligated vectors were transformed into *E. coli* DH5a and selected for through growth on LB agar plates containing 100 µg/mL ampicillin.

To construct vectors for the expression of rLILRB3-His with domain deletions, inverse PCR was performed using pcDNA3.4-LILRB3-His as the template. Forward and reverse primers contained a *Not*I restriction site [Supplementary Data 4]. To construct vectors for the expression of rLILRB3-His with single amino acid substitutions, inverse PCR was performed using primers outlined in Supplementary Data 4, and as outlined in ref. ^84^. PCR amplification was performed using Phusion High-Fidelity Taq Polymerase (Thermo Fisher Scientific) and thermocycling as follows: 1 cycle (98 °C for 2 min), 25 cycles (98 °C 15 s, 62 °C 30 s and 72 °C 5 min), and 1 cycle (72 °C 10 min). Amplicons for domain deletions were digested with *Not*I at 37 °C for 1 h and then with *Dpn*I at 37 °C for 1 h. Ligation was then performed using T4 DNA ligase at room temperature for 30 min, before transformation into *E. coli* DH5a and selection for transformants by growth on LB agar plates containing 100 µg/mL ampicillin. To construct vectors for the expression of rLILRB3-D1-D2-D3-mut-D4-His, inverse PCR was performed to amplify pcDNA3.4-LILRB3-His to replace the original loop (L3) coding sequence of LMAGQIYDTV with the coding sequence of loop 1 (L1) of LVASGFYNKP. Forward and reverse primers are listed in Supplementary Data 4. PCR amplification was performed using Phusion High-Fidelity Taq Polymerase (Thermo Fisher Scientific) and thermocycling as follows: 1 cycle (98 °C for 2 min), 25 cycles (98 °C 15 s, gradient 58–68 °C 30 s, and 72 °C 5 min), and 1 cycle (72 °C 10 min). Amplicons were digested with *Nhe*I at 37 °C for 1 h and then with *Dpn*I at 37 °C for 1 h, then ligated to recircularise the plasmid using T4 DNA ligase at room temperature for 30 min. The ligation reaction was transformed into *E. coli* DH5a. Selection for transformants was performed by growth on LB agar plates containing 100 µg/mL ampicillin.

### Expression and purification of bacterial proteins

The surface proteins β and α were previously purified from GBS cultures^85^. Wildtype β protein domains (B6N, IgABR, B6C, IgI3 and B75KN) were expressed in *E. coli* and purified using HIStrap columns and affinity chromatography, as previously described^41^. To construct vectors for the expression of rB6C-His or rB75KN-His with single amino acid substitutions, inverse PCR was performed using the pRSETC-B6C-His and pRSETC-B75KN-His vectors as the templates, respectively, as described^84^. PCR amplification was performed using Phusion High-Fidelity Taq Polymerase (Thermo Fisher Scientific) and thermocycling as follows:- 1 cycle (98 °C for 2 min), 25 cycles (98 °C 15 s, 60 °C 30 s and 72 °C 5 min), and 1 cycle (72 °C 10 min). Amplicons were digested with *Dpn*I at 37 °C for 1 h and ligated together using T4 DNA ligase. Ligated vectors were transformed into *E. coli* DH5a and selected for through growth on LB agar plates containing 100 µg/mL ampicillin. rB6C-His or rB75KN-His variants were expressed in *E. coli* and purified using HIStrap columns and affinity chromatography, as previously described^41^.

### Binding of rLILR to bacteria

Flow cytometry-based assays were performed^26,41^. In brief, 6 × 10^6^ of mid-logarithmic phase bacteria were incubated with His-tagged or Fc-tagged rLILRB at 4 °C for 1 h. After washing in PBS + 0.1% bovine serum albumin (BSA), bacteria were incubated with FITC-conjugated anti-His (ThermoFisher) or FITC-conjugated anti-human-IgG (SouthernBiotech) at 4 °C for 1 h. After washing in PBS + 0.1% BSA and fixation in 1% paraformaldehyde (PFA), bacterial fluorescence was measured by flow cytometric analysis.

### Binding of rLILR to bacterial proteins

For ELISA-based assays, ninety-six-well flat-bottom plates were coated overnight with bacterial proteins (at 4 °C) or with bacteria (at room temperature, fixed in 20% isopropanol at an absorbance of OD_600_ = 1). After washing, wells were blocked with 5% BSA in PBS to block non-specific interactions for 1 h, and incubated with rLILRB3-Fc, rLILRB2-Fc (1 μg/mL) or rLILRB3-His, rPIR-B-His (10 μg/mL) at room temperature for 1 h. After washing with PBS + 0.05% Tween-20, the wells incubated with rLILRB3-His or rPIR-B-His were incubated with mouse anti-His mAb at room temperature for 1 h. After washing with PBS + 0.05% Tween-20, the wells were incubated with HRP-conjugated goat anti-human-IgG (Life Technologies) or HRP-conjugated anti-mouse-IgG at room temperature for 1 h. After washing with PBS + 0.05% Tween-20, colour development was performed using TMB substrate (Invitrogen) and neutralised using Stop Solution for TMB substrate (Invitrogen).

Flow cytometry-based assays were performed as previously described^26,41^. In brief, biotinylated proteins were attached to Streptavidin magnetic beads (ThermoFisher) or His-tagged proteins were attached to Dynabead His Pulldown (ThermoFisher) following the manufacturer’s protocol. 3 × 10^5^ DB were incubated with rLILR-His or rLILR-Fc at room temperature for 1 h, washed with PBS + 0.5% BSA, and probed with FITC-conjugated anti-His (ThermoFisher) or PE-conjugated anti-IgG-Fc (SouthernBiotech). After washing with PBS + 0.5% BSA, the fluorescence of DB was measured by flow cytometry analysis.

### Protein structure determination by X-ray crystallography and structural analysis

Purified seleno-L-methionine labelled B6C was exchanged into 10 mM Tris (pH 8.0), 50 mM NaCl, and concentrated to approximately 7.5 mg/mL, as determined by the OD_280_ reading and the theoretical extinction coefficient. Initial crystallisation conditions were identified using a high-throughput sitting drop approach. Thereafter, vapour-diffusion hanging drops were created by mixing 1 µL of the protein solution with 1 µL of a precipitant solution containing 0.1 M citric acid (pH 3.7), 35% (v/v) polyethylene glycol 200, and equilibrating over 500 µL of the precipitant solution. Rod-shaped crystals appeared within 24 h and reached their final size in approximately 2 days. Crystals for X-ray diffraction analysis were harvested directly from their mother liquor and flash-cooled in liquid N_2_.

Monochromatic X-ray diffraction data were collected at beamline 5.0.1 of the Advanced Light Source at Lawrence Berkeley National Laboratory. Reflections were indexed, integrated, and scaled using HKL-2000 software^86^. Bijvoet pairs were kept separate to allow for structure solution using the anomalous signal present in the reflections. Initial experimental phase information was obtained through the AutoSol program^87^ of the PHENIX software suite^88^. Two selenium sites were identified within the asymmetric unit of the P2_1_2_1_2 unit cell, followed by automated chain tracing using AutoBuild within PHENIX. A combination of manual model building in COOT^89^, along with reciprocal space positional and individual B-factor refinement, was used to obtain the final model in PHENIX. The final model consists of two copies of the B6C domain without chain breaks, along with 70 ordered solvent molecules. The final model and structure factors have been deposited in the PDB under accession code 9MN2. Additional information on X-ray diffraction data collection and structure refinement can be found in the accompanying Table 1.

### Small-angle X-ray scattering coupled to in-line size-exclusion chromatography (SEC-SAXS) data collection

All SAXS data were collected at the SIBYLS beamline (Lawrence Berkeley National Laboratory)^90,91^. Small-angle X-ray scattering (SAXS) data from various samples were collected by coupling the SAXS setup with a size exclusion column. The experiments were performed at a sample-to-detector distance of 2100 mm and a wavelength of 1.127 Å, yielding scattering vectors (*q*), ranging from 0.01 Å⁻¹ to 0.4 Å⁻¹, where the scattering vector is defined as *q* = 4*π*sin*θ*/*λ*, and 2*θ* is the scattering angle. Size-exclusion chromatography (SEC) was executed using an Agilent 1260 series HPLC with a Shodex analytical column^92^. Samples of protein complexes were prepared from the requisite monomers by incubating at the appropriate molar ratio. A 60 μl sample at a 5 mg/ml concentration was injected at a flow rate of 0.65 ml/min onto a Shodex KW-803 column at 4 °C. A total of 906 successive 2-second frames were collected. Data normalisation was carried out against the intensity of the transmitted beam, followed by radial averaging, and the solvent-blank scattering was subtracted.

### SAXS solution structure modelling

High-resolution crystal structures for B75KN-CTD (PDB:7S0R) and B6C (PDB: 9MN2) were used for structural modelling, whereas a cryo-EM model of LILRB3 was employed (PDB: 8GRX). Missing residues were added using the MODELLER feature of UCSF-ChimeraX^93,94^. Models for the B6C/LILRB3 and B75KN-CTD/LILRB3 complexes were calculated using FoXSDock^95^ to perform rigid-body docking. The model with the highest overall score of the 500 models generated was accepted as a starting point for further analysis. Subsequently, the theoretical SAXS profile for each sampled conformation was calculated, followed by the enumeration of the best-scoring models using the ensemble function implemented in MultiFoXS^96^. The calculated SAXS profiles for the B6C/LILRB3 and B75KN-CTD/LILRB3 models were generated and fit to the experimental data using FoXS. Ab initio density envelopes were calculated from solution scattering data for the B6C/LILRB3 and B75KN-CTD/LILRB3 complexes using DENSS^97^.

### Surface plasmon resonance analysis of β protein interactions with LILRB3

Direct binding of recombinant β protein fragments and mutants thereof to purified LILRB3 was evaluated by Surface Plasmon Resonance (SPR). All experiments were performed at 25 °C using a Biacore T-200 instrument (Cytiva Life Sciences) with a running buffer of HBS-T (20 mM HEPES (pH 7.4), 140 mM NaCl, and 0.005% (v/v) Tween-20) and a flow rate of 30 μL/min. For experiments with covalently attached ligands, the surface was prepared by immobilising rB6c and rB75KN on CMD200M chips (Xantec Bioanalytics GmbH; Dusseldorf, Germany) using standard amine coupling. rLILRB3 (1.95 nM–1 µM) or its truncated variants (19.5 nM–10 µM) were injected over the biosensor surface in reference subtraction mode using a two-fold series of increasing concentrations. For capture experiments, the experimental surface was prepared by capturing biotinylated-LILRB3 on an SAD200M chip (Xantec Bioanalytics GmbH; Düsseldorf, Germany), while the reference surface was created by capturing biotin alone. Recombinant B6C or B75KN-CTD proteins were injected over the biosensor surface in reference subtraction mode using a two-fold series of increasing concentrations ranging from 19.5 nM to 10 μM. In all experiments, each experimental cycle consisted of a 2-min association phase followed by a 3-minute dissociation phase. The biosensor surface was regenerated by two injections of 2 M NaCl for 30 s. Sensorgram data sets were analysed using Biacore T-200 Evaluation software v3.2 (Cytiva Life Sciences) with various mathematical models. Data were globally fitted to a simple affinity (dose/response) model to determine an apparent *K*_D_ value without considering kinetic parameters. Thereafter, sensorgram series were fitted to a 1:1 binding model to derive both *K*_D_, as well as association (*k*_a_) and dissociation (*k*_d_) rate constants where applicable.

### Competition binding assays by SPR

Additional SPR experiments were performed to evaluate binding of the recombinant protein rB6C–IgI3–B75KN–His to LILRB3. A CMD200M sensor chip (Xantec Bioanalytics, Düsseldorf, Germany) was prepared by standard amine coupling of NeutrAvidin (Thermo Fisher Scientific) to the chip surface. LILRB3 was biotinylated using EZ-Link NHS-PEG4-Biotin (Thermo Fisher Scientific); excess reagent was removed by desalting before capture. Biotinylated LILRB3 was then captured in the active flow cell to a final surface density of 1,074 response units (RU). The reference flow cell was prepared by capturing biotin (10 µM) alone onto the NeutrAvidin surface to block residual binding sites. For analyte-only injections, rB6C–IgI3–B75KN–His was used at a final concentration of 1 µM. Then, for competition assays, the analyte was premixed with rLILRB3 at a 1:1 or 1:2 molar ratio (analyte: competitor) for a short period prior to injection. The association period was monitored for 2 min, followed by 3 min of dissociation. Surfaces were regenerated with two pulses of 10 mM glycine-HCl, pH 2.2, containing 1.5 M NaCl. The outcome of the experiment was assessed by superimposing the sensorgrams from each injection cycle.

### Circular dichroism

Far-UV circular dichroism spectra were recorded at room temperature on either a Jasco J-815 CD spectrophotometer (B6C and B75KN-C variants) or a Chirascan V100 instrument (rLILRB3-His and full-length β variants)^98,99^. Samples were prepared in either ddH_2_O alone (B6C or B75KN-C variants), 10 mM sodium phosphate, 50 mM sodium fluoride, pH 7.4 (LILRB3-his variants) or standard PBS (β and β^ΔLILRB3^). Measurements were performed in a 1 mm pathlength quartz cuvette over a wavelength range of 190–260 nm. Five experimental scans were averaged for each protein, buffer baseline was subtracted, and a Savitzky–Golay smoothing algorithm was applied. Where indicated, the spectra were scaled to the ellipticity at 222 nm.

### Adhesion of bacteria to eukaryotic cells

U937 cell lines or primary human neutrophils were diluted to 2 × 10^6^ cells/ml and mixed with FITC-labelled *S. agalactiae* or *L. lactis* strains at an MOI of 10. After centrifugation at 100 x *g* for 2 min, the cultures were incubated for 30 min at 37 °C. After washing and fixation in 1% PFA, the fluorescence of cells was measured by flow cytometry.

### Induction of GFP Expression in LILRB3-CD3ζ reporter 2B4 NFAT-GFP T cells

Murine 2B4 NFAT-GFP T (2B4T) cell lines were previously described^18^. Cells were diluted to 2.5 × 10^5^ cells/ml. Two hundred microliters of cell suspension were seeded into wells of a 96-well tissue culture plate, centrifuged at 100 × *g* for 2 min, and cultured for 18 h at 37 °C with 5% CO_2_. The fluorescence of the cultured cells was measured by flow cytometry. The wells of the 96-well plate were previously coated overnight with 50 μl of anti-mouse-CD3 (5 μg/ml), purified β protein (5 μg/ml) or recombinant bacterial domains (5 μg/ml) diluted in PBS. Alternatively, the wells of the 96-well plate were previously coated overnight with 50 μl of *S. agalactiae* strains fixed in 20% isopropanol (absorbance at OD_600_ = 1).

### Immunoprecipitation and western blotting

3 × 10^6^ THP-1 cells that stably-express LILRB3 were lysed in ice-cold NP-40 lysis buffer (ThermoFisher) containing 1 mM AEBSF (ThermoFisher), 1 mM PMSF (ThermoFisher) and 10 mM leupeptin B (Sigma Aldrich). After centrifugation for 20 minutes at 15,000 × *g* and 4 °C, lysates were incubated with 2 μg mouse anti-LILRB3 (clone 222821; R&D Biosystems) and Dynabeads protein G (Invitrogen) overnight at 4 °C. Immunoprecipitates were washed three times with NP-40 lysis buffer, then separated by sodium dodecyl sulphate–polyacrylamide gel electrophoresis (SDS–PAGE) using 10% gels. After transfer of proteins by Western blot, nitrocellulose membranes were blocked in Tris-buffered saline with 0.1% Tween 20 (TBS-T) containing 5% bovine serum albumin (Sigma Aldrich) for 2 h at room temperature. LILRB3 was detected using anti-LILRB3 mAb (clone 222821), and tyrosine phosphorylated proteins were detected with monoclonal Phospho-Tyrosine Mouse mAb (4G10; Cell Signalling Technology). Primary antibodies were detected using HRP-conjugated goat anti-mouse IgG (ThermoFisher). Signals were detected using Pierce ECL Western Blotting Substrate solution. All washes of nitrocellulose membranes were performed with TBS-T. Blots are presented in Source Data File.

### ROS production by human neutrophils

The ROS assay was performed as described^19^, with modifications. White 96 well-plates were coated at 4 °C overnight with both 5 μg/ml of anti-FcγRIIa (clone IV.3, STEMCELL Technologies) or anti-FcαRI (clone MIP8a, Bio-Rad) or isotype IgG1 (clone 11711, R&D), and 10 μg/ml of β protein domains diluted in 100 μl of PBS. Peripheral blood from healthy donor volunteers was collected into tubes with sodium heparin. Granulocytes were purified by centrifugation on a Ficoll gradient, and resuspended in 20 mM HEPES, pH 7.4, 140 mM NaCl, 1 μM CaCl_2_, 5 mM glucose, and 0.1% BSA to 1 × 10^6^ cells/ml. Then, 50 μl of granulocyte suspension was transferred to coated wells of the white plate that contained 50 μl of 20 mM HEPES pH 7.4, 140 mM NaCl, 1 μM CaCl_2_, 5 mM glucose, 0.1% BSA and 10 μM Amplex Red (Thermo Fisher). Where needed, stimulation with 10 ng/ml phorbol 12-myristate 13-acetate in solution was used as a positive control. The production of ROS was measured as an increase in fluorescence signal (excitation at 544 nm, emission at 590 nm), measured at 2- or 3-min intervals for up to 120 min at 37 °C in a plate reader. The level of ROS production is plotted as Amplex Red fluorescence signal at the designated time point, with subtraction of the background fluorescence signal in unstimulated granulocytes.

### Phagocytic bacterial killing assays

*S. agalactiae* strains were grown to an OD_600_ of 0.4 as described above and resuspended in PBS with 0.1% BSA to 1 × 10^8^ CFU/mL. Where needed, bacterial strains were pre-opsonised with 10% heat-inactivated human serum for 30 min at 37 °C. Appropriate numbers of bacteria to reach the designated MOIs were then mixed with 1 × 10^6^ purified human neutrophils, in a final volume of 100 μl. One hour after incubation at 37 °C, samples were lysed in 0.3% saponin, serially diluted into sterile PBS and plated onto TH agar plates. After culturing at 37 °C for 16 h, the % survival was calculated as CFU after incubation with neutrophils relative to CFU of the inoculum at time zero.

### Bioinformatics

To assess the carriage of *bac* gene distribution in *S. agalactiae* populations, the presence and absence of *bac* in isolates belonging to the 10 major clonal complex (CC) lineages (CC1, CC10/12, CC17, CC19, CC23, CC26, CC283, CC327, CC452, CC459) was analysed using the PubMLST database^100^.

### Statistics and reproducibility

All quantitative data were analysed using GraphPad Prism 10.4.1 software. All data are represented as mean ± s.d. calculated using the GraphPad Prism 10.4.1 software, unless indicated otherwise. Statistical details of the experiments are provided in the respective figure legends and in each method section pertaining to the specific technique applied. No statistical method was used to predetermine sample size. No data were excluded from the analyses. The experiments were not randomised. The Investigators were not blinded to allocation during experiments and outcome assessment.

### Reporting summary

Further information on research design is available in the Nature Portfolio Reporting Summary linked to this article.

## Supplementary information

Supplementary Information

Description of Additional Supplementary File

Supplementary Data 1

Supplementary Data 2

Supplementary Data 3

Supplementary Data 4

Reporting summary

Transparent Peer Review File

## Source data

source data

## Data Availability

The crystal structure data generated in this study have been deposited in the Protein Data Bank (PDB) database under accession code 9MN2. The SAXS data generated in this study have been deposited in the SASBDB under accession codes SASDXH8, SASDVA4, SASDVB4, SASDXG8 and SASDXF8. Source data are provided with this paper.
