# Supplementary material for: Bacterial targeting of the neutrophil inhibitory receptor LILRB3 to evade antibody immunity
Source: Nat Commun. 2026 Jun 11;17:7463. doi: 10.1038/s41467-026-74098-6 (PMC13408158; doi:10.1038/s41467-026-74098-6)
Supplement: Supplementary file 1 — Supplementary Information [file 41467_2026_74098_MOESM1_ESM.pdf]

Supplementary Information

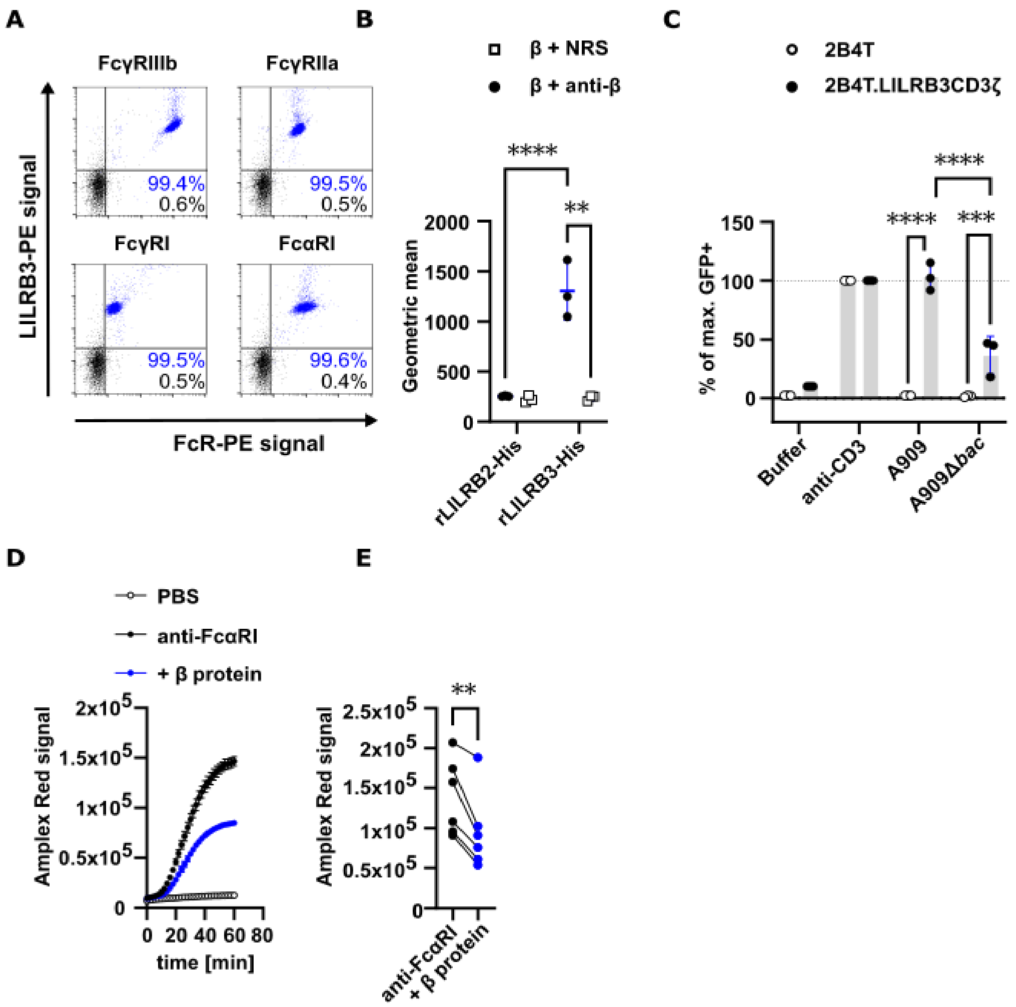

Supplementary Figure 1: β binds and cross-links the inhibitory LILRB3 receptor (A) Dual expression analysis of LILRB3 and FcγRI, FcγRIIa, FcγRIIIb and FcαRI on human neutrophils, measured using flow cytometry (representative of *n* = 3 independent experiments). Dual stains for anti-LILRB3 and anti-FcR are shown in blue, whilst dual stains for isotype controls are shown in black. **(B)** Binding of purified β protein to rLILRB3-coated or rLILRB2-coated dynabeads, quantified using flow cytometry. NRS = normal rabbit sera. Mean ± s.d. of *n* = 3 independent experiments, with two-way ANOVA where β+anti-β/rLILRB2 vs β+anti-β/rLILRB3 ****p<0.0001, β+anti-β/rLILRB3 vs β+NRS/rLILRB3 **p=0.0012. **(C)** Stimulation of GFP production in 2B4T reporter cells after incubation with *S. agalactiae* strains, quantified using flow cytometry. The percentage of GFP-positive cells was calculated and normalized against cells stimulated with anti-CD3. Mean ± s.d. of *n* = 3 independent experiments, with two-way ANOVA with Šídáks multiple

comparison test, where 2B4T/A909 vs 2B4T.LILRB3CD3ζ/A909 ****$p<0.0001$, 2B4T/A909Δ*bac* vs 2B4T.LILRB3CD3ζ/A909Δ*bac* ***$p=0.003$, and 2B4T.LILRB3CD3ζ/A909 vs 2B4T.LILRB3CD3ζ/A909Δ*bac* ****$p<0.0001$. **(D** and **E)** Modulation of FcαRI-mediated respiratory burst in human neutrophils by rβ protein, quantified using Amplex Red. In D, a representative Amplex Red Signal plot is shown using phosphate buffered saline (PBS) as control. In E, the consolidated data for $n = 6$ independent experiments are shown, where the Amplex Red signal at 60 minutes is shown after subtraction of signal from IgG1-stimulation. Mean ± s.d. is shown, with *t* test, where **$p=0.0036$.

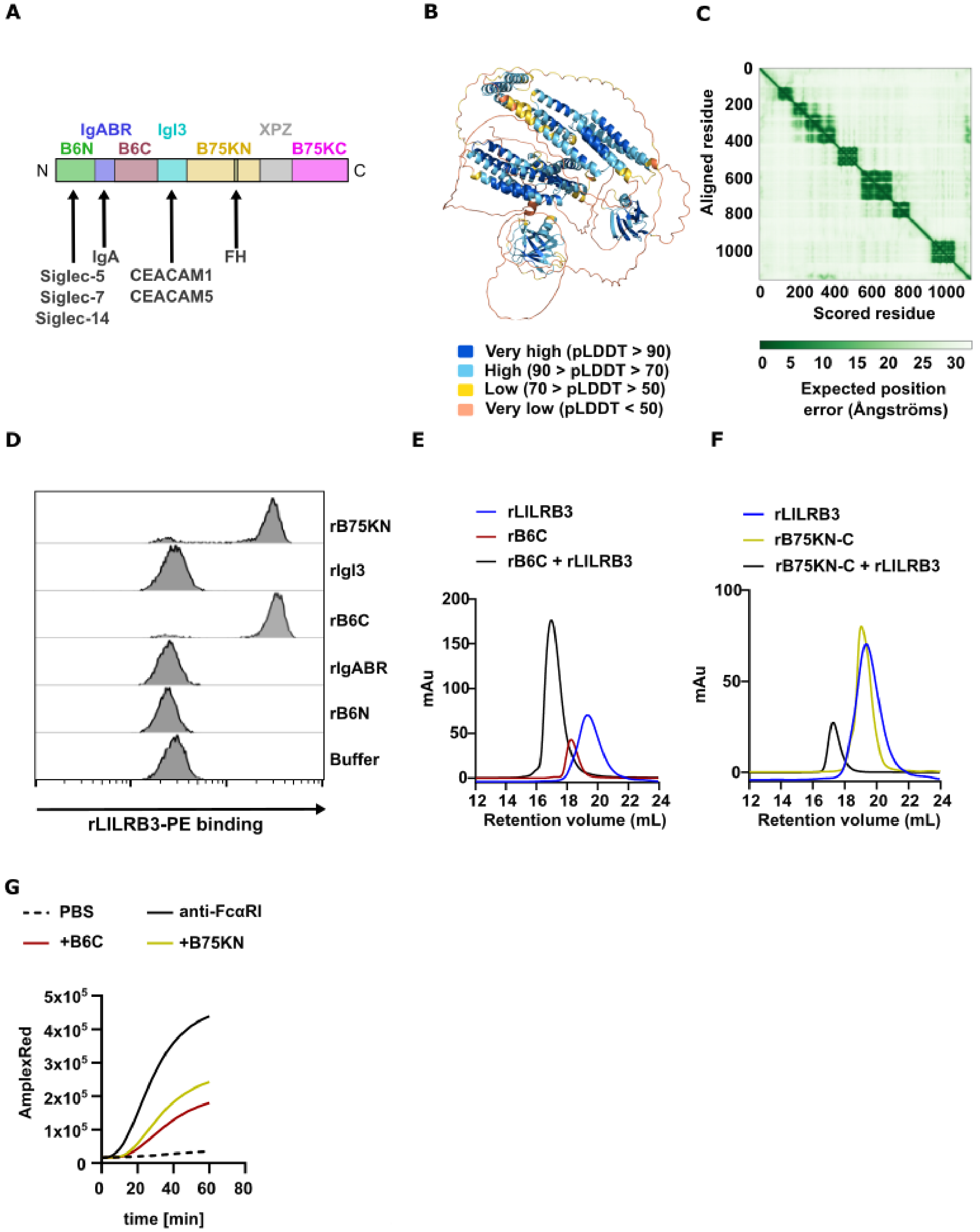
A
IgABR
IgI3
XPZ
B6N
B6C
B75KN
B75KC
N
C
IgA
FH
Siglec-5
Siglec-7
Siglec-14
CEACAM1
CEACAM5
B
Very high (pLDDT > 90)
High (90 > pLDDT > 70)
Low (70 > pLDDT > 50)
Very low (pLDDT < 50)
C
Aligned residue
Scored residue
Expected position error (Ångströms)
D
rB75KN
rIgI3
rB6C
rIgABR
rB6N
Buffer
rLILRB3-PE binding
E
rLILRB3
rB6C
rB6C + rLILRB3
mAu
Retention volume (mL)
F
rLILRB3
rB75KN-C
rB75KN-C + rLILRB3
mAu
Retention volume (mL)
G
PBS
anti-FcαRI
+B6C
+B75KN
AmplexRed
time [min]

Supplementary Figure 2: B6C and B75KN of β protein suppress ROS production by neutrophils. (A) Schematic of β protein showing location of domains, with known human ligand binding sites indicated. Human factor H (FH) binds to a QHLQKKN loop in B75KN domain. **(B)** AlphaFold prediction for a β protein (Accession: AF-P27951-F1), coloured by model confidence measured by predicted local distance difference test (pLDDT). **(C)** The expected positional error panel of the predicted β protein structure shows dark green patches for each of the structured domains, indicating that they are well defined. However, note that the green shading is weak between structured domains, indicating that the inter-domain configuration is not well defined. **(D)** Representative flow plots showing binding of rLILRB3 and rLILRB2 to rB6N-, rIgABR-, rB6C-, rIgI3- and rB75KN-coated Dynabeads. **(E)** Size exclusion chromatography analysis of rLILRB3, rB6C and rLILRB3/rB6C complex. **(F)** Size exclusion chromatography analysis of rLILRB3, rB75KN and rLILRB3/rB75KN-C complex. **(G)** Modulation of FcαRI-mediated respiratory burst in human neutrophils by rB6C and rB75KN, quantified using Amplex Red. A representative Amplex Red Signal plot for $n = 3$ independent experiments is shown.

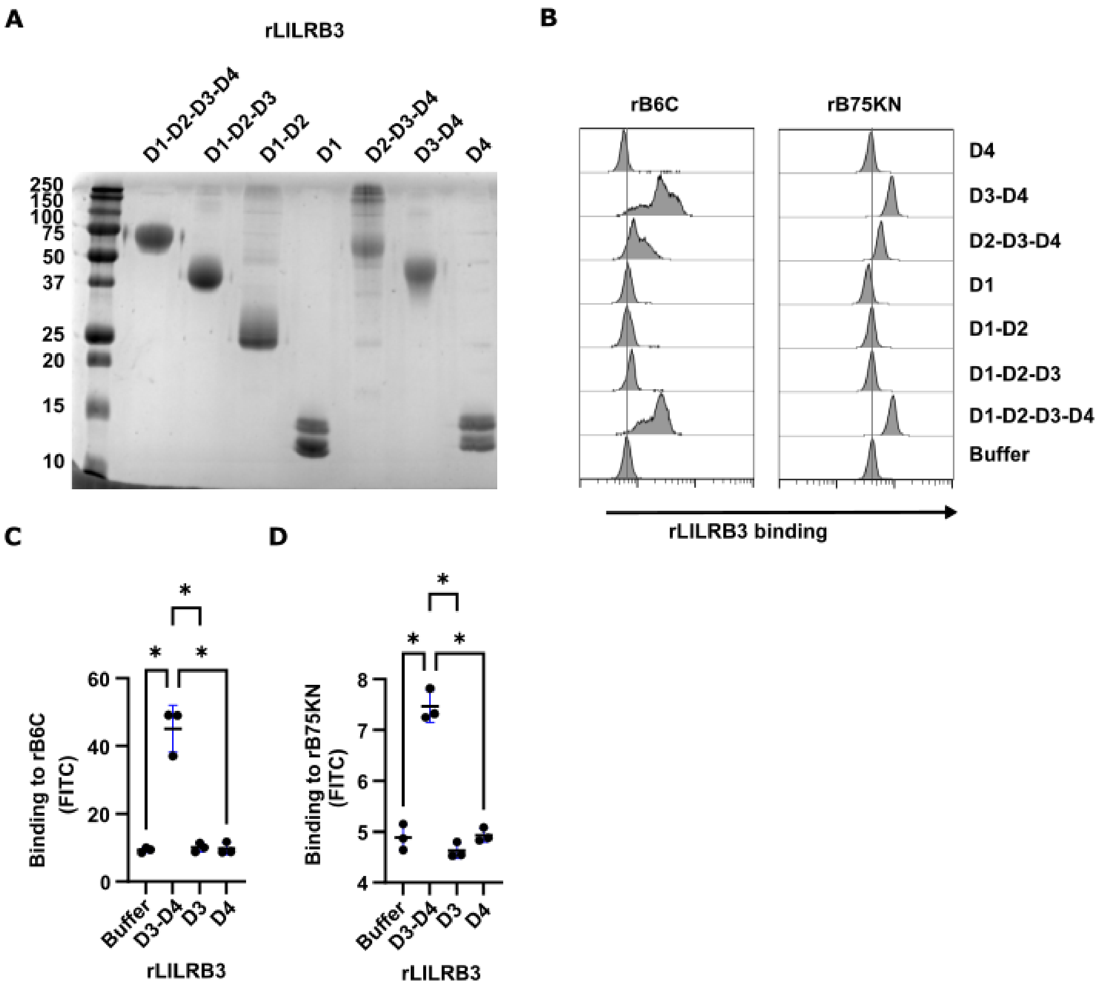

Supplementary Figure 3: LILRB3 D3-D4 binds to B6C and B75KN of β protein. (A) SDS-PAGE showing purified rLILRB3-His variants. **(B)** Representative flow cytometry plots showing binding of rLILRB3-His variants to rB6C-coated and rB75KN-coated Dynabeads. **(C)** Binding of rLILRB3-His constructs to rB6C-coated Dynabeads, quantified using flow cytometry and using FITC-conjugated anti-6xHis. Mean ± s.d. of $n = 3$ independent experiments, with one-way ANOVA, where buffer vs D3-D4 *p=0.0318, D3-D4 vs D3 *p=0.0236, and D3-D4 vs D4 *p=0.0251. **(D)** Binding of rLILRB3-His constructs to rB75KN-coated Dynabeads, quantified using flow cytometry and using FITC-conjugated anti-6xHis. Mean ± s.d. of $n = 3$ independent experiments, with one-way ANOVA, where buffer vs D3-D4 *p=0.0370, D3-D4 vs D3 *p=0.0223, and D3-D4 vs D4 *p=0.0184. In all, each rLILRB3-His construct has varying amounts of Ig domains (D) as annotated.

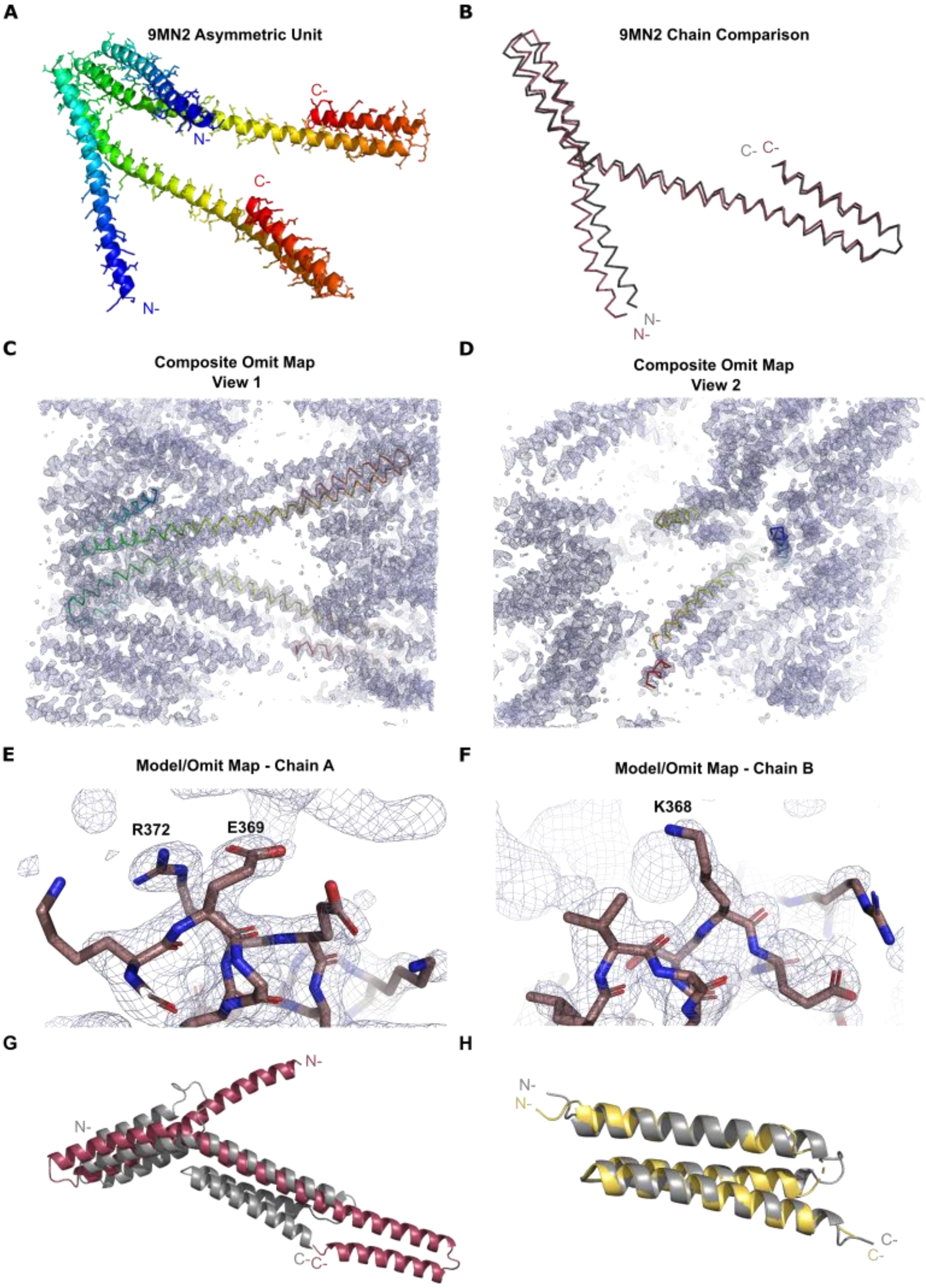
A
9MN2 Asymmetric Unit
C-
N-
C-
N-
B
9MN2 Chain Comparison
C- C-
N-
N-
C
Composite Omit Map
View 1
D
Composite Omit Map
View 2
E
Model/Omit Map - Chain A
R372
E369
F
Model/Omit Map - Chain B
K368
G
N-
N-
C- C-
H
N-
N-
C-
C-

Supplementary Figure 4: Crystal structure of the B6C domain of β protein. (A) Ribbon diagram illustrating both polypeptides within the asymmetric unit of the B6C crystal. Each polypeptide is colored with its N-terminus in blue and its C-terminus in red. Sidechains are illustrated in stick convention. **(B)** Superpositioning of polypeptide chains A (raspberry) and B (grey) within the asymmetric unit of the B6C crystal. Only Cα positions are shown for the sake of clarity. **(C)** Composite omit map for the final 9MN2 structure calculated at 2.05 Å limiting resolution, drawn as a grey mesh (contoured at 1.2σ). The B6C polypeptides are shown in Cα wire convention with their N-termini in blue and their C-termini in red. Note that Chain B appears at the top and Chain A appears at the bottom in this orientation. **(D)** Identical to panel C, except that structure is rotated approximately 90° clockwise in the plane of the viewing page. **(E)** Representative model to composite omit map correlation for Chain A in the vicinity of functionally important residues E369 and R372. The protein is colored with carbon atoms in raspberry, while the composite omit map (contoured at 1.2σ) is drawn as a grey mesh. **(F)** Identical to panel E, except that Chain B is represented in the vicinity of the functionally important residue K368. **(G)** Alignment of the B6C structure (PDB: 9MN2, red/raspberry) with the AlphaFold predicted structure of the B6C domain (Accession: AF-P27951-F1-model_v4_1, grey). **(H)** Alignment of the B75KN-C structure (PDB: 7S0R, yellow) with the AlphaFold predicted structure of the B75KN-C domain (Accession: AF-P27951-F1-model_v4_1, grey).

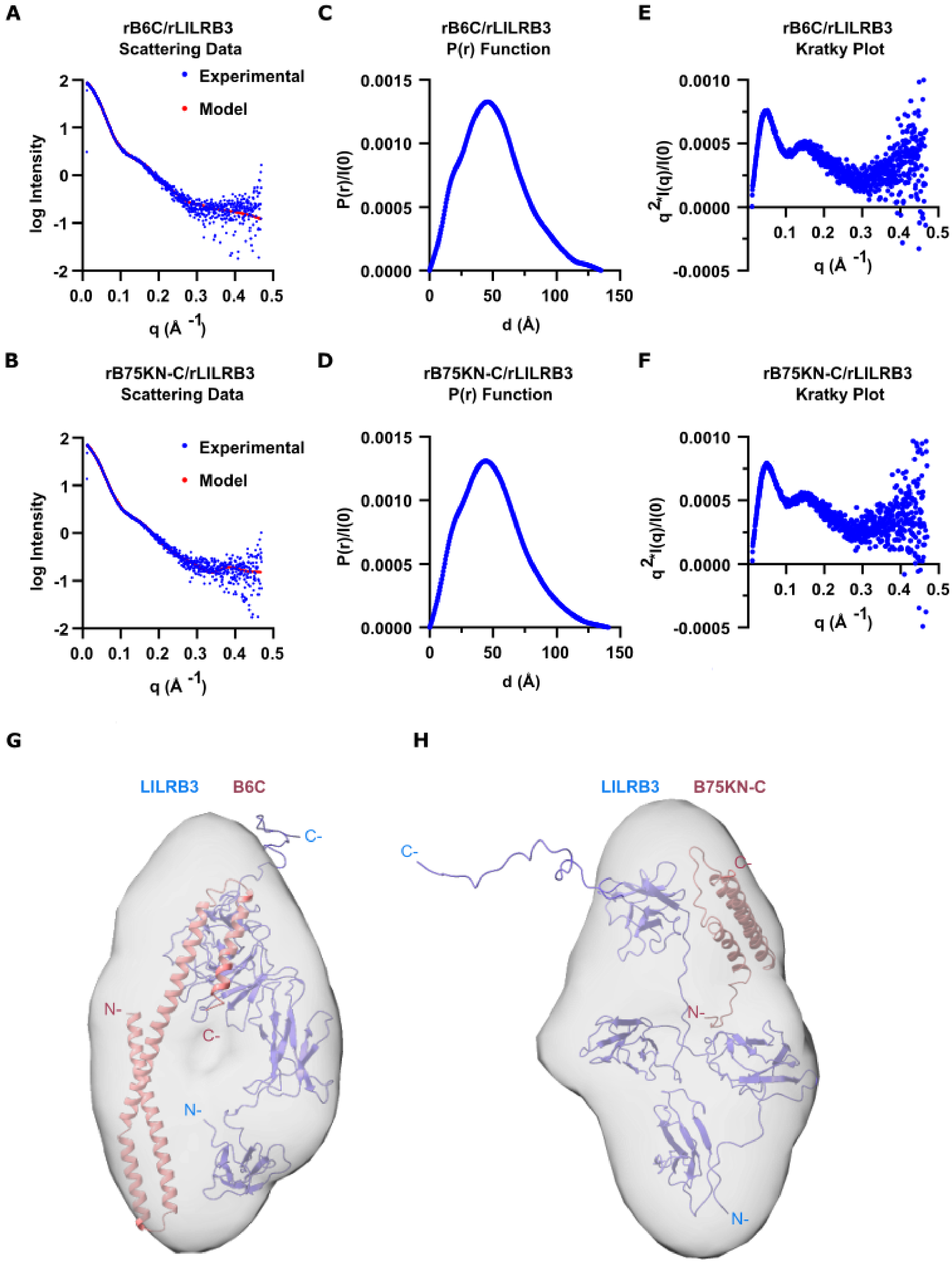

A
rB6C/rLILRB3
Scattering Data
Experimental
Model
log Intensity
q (Å -1)
C
rB6C/rLILRB3
P(r) Function
P(r)/I(0)
d (Å)
E
rB6C/rLILRB3
Kratky Plot
q2*I(q)/I(0)
q (Å -1)
B
rB75KN-C/rLILRB3
Scattering Data
Experimental
Model
log Intensity
q (Å -1)
D
rB75KN-C/rLILRB3
P(r) Function
P(r)/I(0)
d (Å)
F
rB75KN-C/rLILRB3
Kratky Plot
q2*I(q)/I(0)
q (Å -1)
G
LILRB3
B6C
C-
N-
C-
N-
H
LILRB3
B75KN-C
C-
C-
N-
N-

Supplementary Figure 5: SAXS analysis of the B6C/LILRB3 and B75KN-C/LILRB3 complexes. (A) SEC-SAXS profile of B6C/LILRB3 (blue circles) along with the fit (χ^2=0.92) for a model of the structure (red line). **(B)** SEC-SAXS profile of B75KN-C/LILRB3 (blue circles) along with the fit (χ^2=1.02) for a model of the structure (red line). **(C)** Pair-distribution (P(r)) function obtained from SAXS data collected on the B6C/LILRB3 complex. The maximal particle dimension (D_{max}) estimate is 135 Å. **(D)** Pair-distribution (P(r)) function obtained from SAXS data collected on the B75KN-C/LILRB3 complex. The maximal particle dimension (D_{max}) estimate is 141 Å. **(E)** Kratky plot for the SAXS data collected on the B6C/LILRB3 complex. **(F)** Kratky plot for the SAXS data collected on the B75KN-C/LILRB3 complex. **(G)** *Ab initio* density envelope for B6C/LILRB3, along with the best fit model for the complex. B6C is shown in red and LILRB3 is shown in blue. **(H)** *Ab initio* density envelope for B75KN-C/LILRB3, along with the best fit model for the complex. B75KN-C is shown in red and LILRB3 is shown in blue.

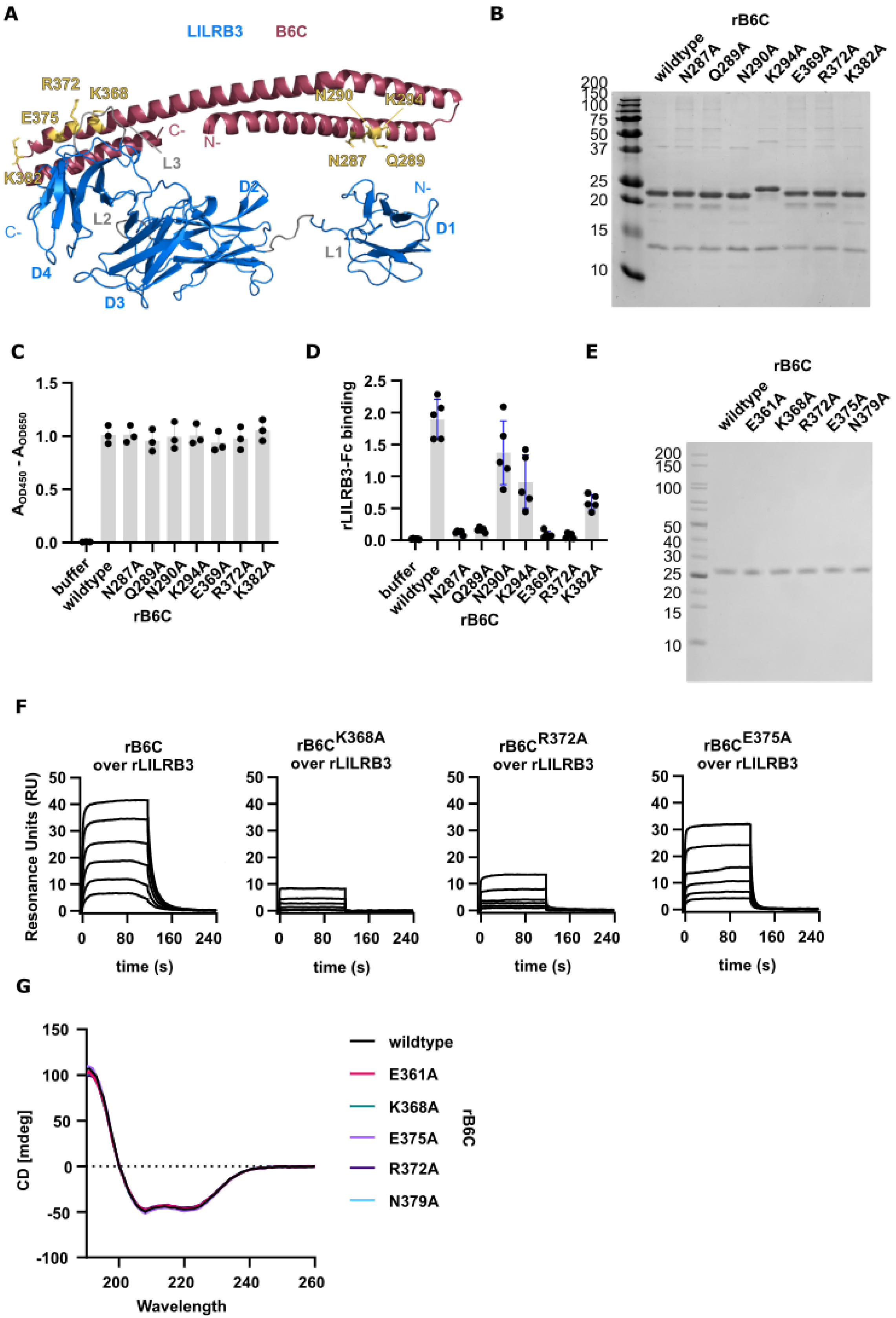

A
LILRB3 B6C
R372 K368 E375 K382 N290 K294 N287 Q289
C- N- L3 L2 L1 D1 D2 D3 D4 N- C-
B
rB6C
wildtype N287A Q289A N290A K294A E369A R372A K382A
200 150 100 75 50 37 25 20 15 10
C
A_{OD450} - A_{OD650}
1.5 1.0 0.5 0.0
buffer wildtype N287A Q289A N290A K294A E369A R372A K382A
rB6C
D
rLILRB3-Fc binding
2.5 2.0 1.5 1.0 0.5 0.0
buffer wildtype N287A Q289A N290A K294A E369A R372A K382A
rB6C
E
rB6C
wildtype E361A K368A R372A E375A N379A
200 150 100 50 40 30 25 20 15 10
F
rB6C over rLILRB3
rB6C^{K368A} over rLILRB3
rB6C^{R372A} over rLILRB3
rB6C^{E375A} over rLILRB3
Resonance Units (RU)
50 40 30 20 10 0
0 80 160 240
time (s)
G
CD [mdeg]
150 100 50 0 -50 -100
200 220 240 260
Wavelength
wildtype
E361A
K368A
E375A
R372A
N379A
rB6C

Supplementary Figure 6: Analysis of the B6C interface. (A) A close-up view of the B6C (raspberry) binding interface of LILRB3 (blue, grey). Residues selected to test for their importance in the binding of B6C to LILRB3, and for which alanine substitution proteins were successfully expressed and purified, are highlighted in gold. **(B)** SDS-PAGE showing purified rB6C-His wildtype and mutated variants. **(C)** Binding of rB6C-His wildtype and mutated variants to a 96-well ELISA microtitre plate, quantified by ELISA and using HRP-conjugated anti-6xHis. Mean ± s.d. of *n* = 3 independent experiments. **(D)** Binding of rLILRB3-Fc to coated-rB6C variants, quantified by ELISA and using HRP-conjugated anti-human-IgG. Mean ± s.d. of *n* = 5 independent experiments. **(E)** SDS-PAGE showing purified rB6C-His wildtype and mutated variants utilised for SPR analysis. **(F)** Reference-corrected SPR series for binding of rB6C variants to immobilised rLILRB3-biotin, with the experimental data shown as black traces. **(G)** Circular dichroism spectroscopy analysis of rB6C-His wildtype and mutated variants used in SPR analysis.

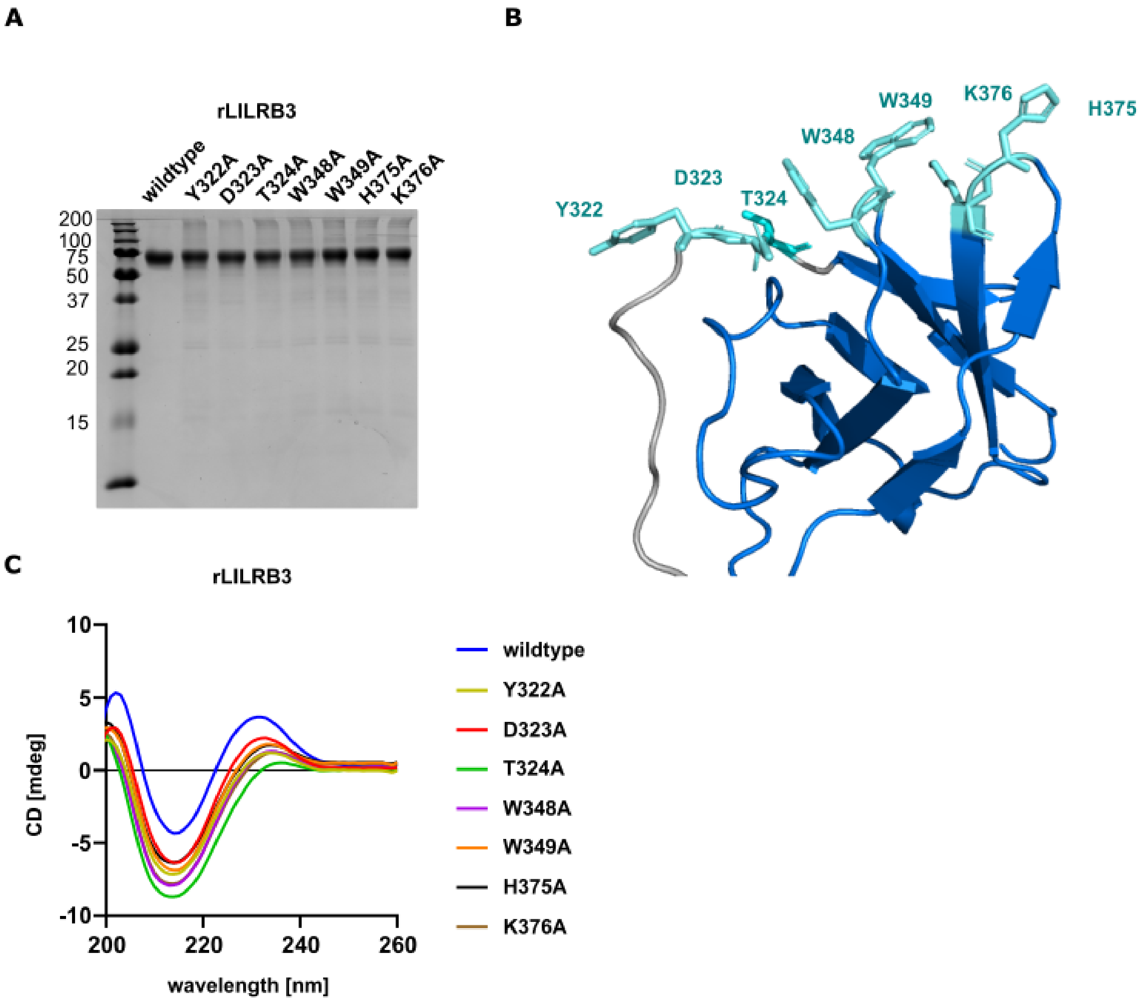

Supplementary Figure 7: Analysis of the LILRB3 interface. (A) SDS-PAGE showing purified rLILRB3 wildtype and mutated variants. **(B)** A close-up view of Ig domain 4 of LILRB3 obtained from the corresponding AlphaFold model. Residues selected to be tested for their importance in the binding of LILRB3 to B6C/B75KN, and for which alanine substitution proteins were successfully expressed and purified are highlighted (cyan). Residues are located in loops between β-strands. **(C)** Circular dichroism spectroscopy analysis of rLILRB3 wildtype and mutated variants, where each line represents a mean from n = 5 measurements.

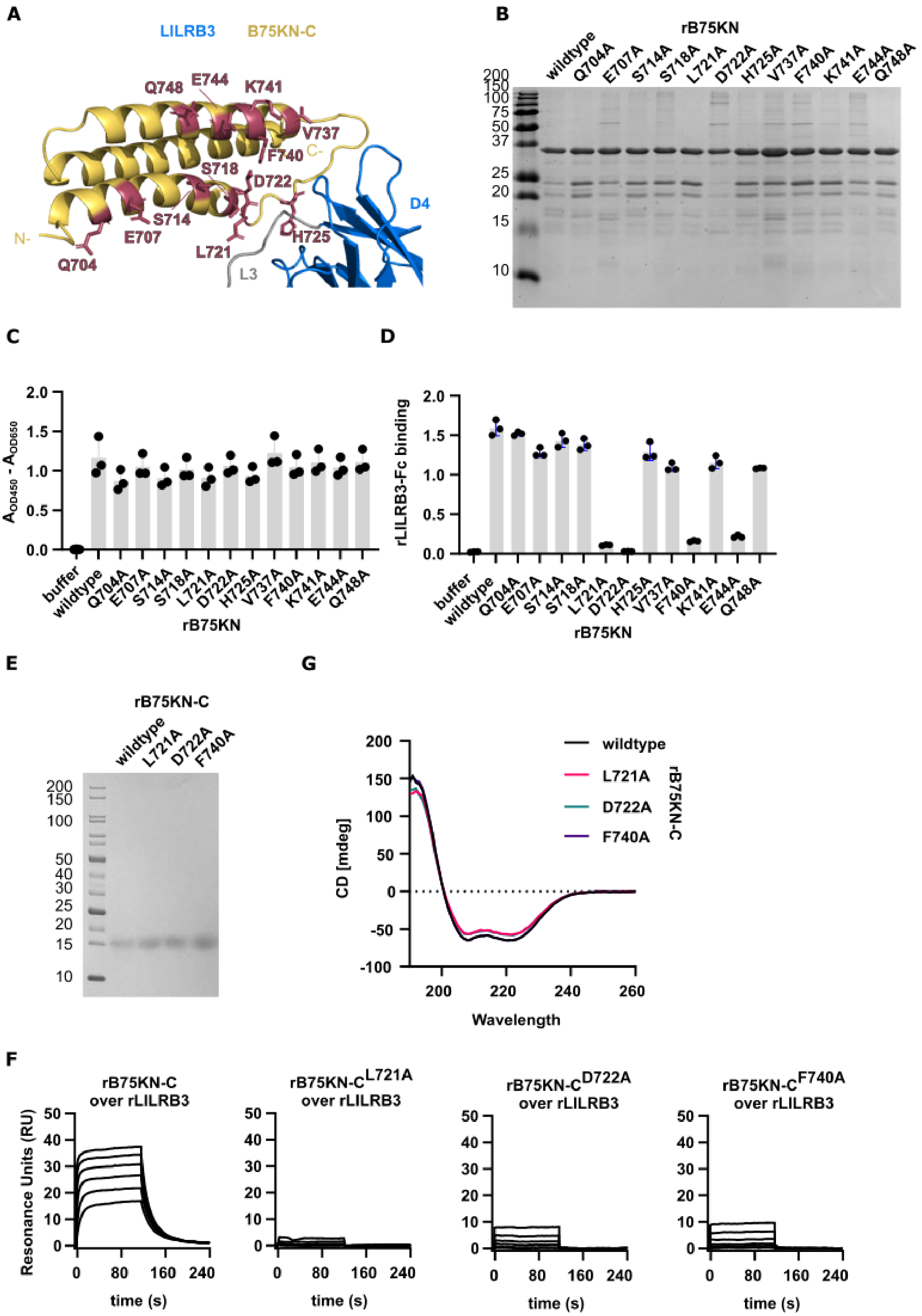

A
LILRB3
B75KN-C
Q748
E744
K741
V737
F740
C-
S718
D722
D4
N-
S714
E707
H725
L721
Q704
L3
B
rB75KN
wildtype Q704A E707A S714A S718A L721A D722A H725A V737A F740A K741A E744A Q748A
200 150 100 75 50 37 25 20 15 10
C
buffer wildtype Q704A E707A S714A S718A L721A D722A H725A V737A F740A K741A E744A Q748A
rB75KN
D
rLILRB3-Fc binding
buffer wildtype Q704A E707A S714A S718A L721A D722A H725A V737A F740A K741A E744A Q748A
rB75KN
E
rB75KN-C
wildtype L721A D722A F740A
200 150 100 50 40 30 25 20 15 10
G
CD [mdeg]
Wavelength
wildtype
L721A
D722A
F740A
rB75KN-C
F
rB75KN-C over rLILRB3
rB75KN-C^L721A over rLILRB3
rB75KN-C^D722A over rLILRB3
rB75KN-C^F740A over rLILRB3
Resonance Units (RU)
time (s)

Supplementary Figure 8: Analysis of B75KN interface. (A) A close-up view of the B75KN (yellow) binding interface of LILRB3 (blue, grey). Residues selected to test their importance in the binding of B75KN to LILRB3, and for which alanine substitution proteins were successfully expressed and purified, are highlighted in raspberry. **(B)** SDS-PAGE showing purified rB75KN-His wildtype and mutated variants. **(C)** Binding of rB75KN-His wildtype and mutated variants to a 96-well ELISA microtitre plate, quantified by ELISA and using HRP-conjugated anti-6xHis. Mean ± s.d. of n = 3 independent experiments. **(D)** Binding of rLILRB3-Fc to coated-rB75KN variants, quantified by ELISA and using HRP-conjugated anti-human-IgG. Mean ± s.d. of n = 3 independent experiments. **(E)** SDS-PAGE showing purified rB75KN-C-His wildtype and mutated variants utilised for SPR analysis. **(F)** Reference-corrected SPR series for binding of rB75KN-C variants to immobilised rLILRB3-biotin, with experimental data shown as black traces. **(G)** Circular dichroism spectroscopy analysis of rB75KN-C-His wildtype and mutated variants used in SPR analysis.

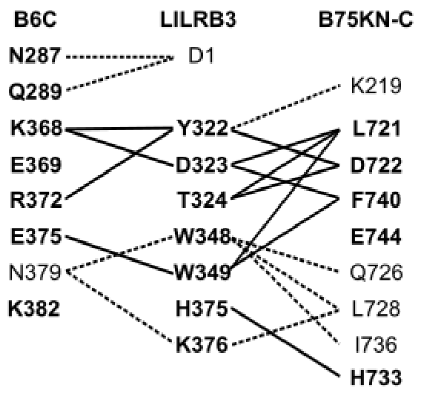

Supplementary Figure 9: Map of predicted interacting residues in B6C/LILRB3 and B75KN/LILRB3 interactions. Residues that when mutated to alanine had significant reduction in binding are in bold. Complete lines represent predicted interacting residues when both residues when mutated had significant reduction in binding. Dashed lines represent predicted interacting residues when only one residue was studied in mutagenesis assays. D1 indicates Ig-like domain 1 in LILRB3.

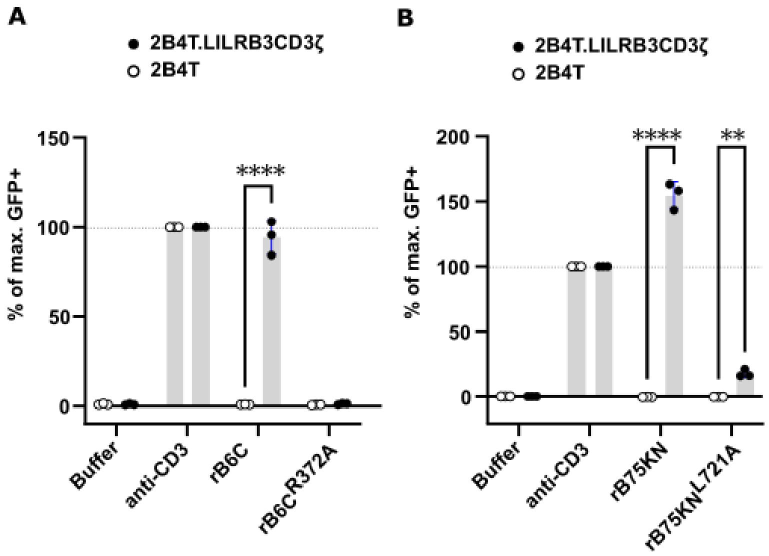

Supplementary Figure 10: Mutation of B6C and B75KN interface residues significantly reduce LILRB3 cross-linking. (A) Stimulation of GFP production in 2B4T reporter cells by rB6C variants, quantified using flow cytometry. The percentage of GFP-positive cells was calculated and normalised against cells stimulated with anti-CD3. Mean ± s.d. of $n = 3$ independent experiments, with two-way ANOVA where 2B4T/rB6C vs 2B4T.LILRB3CD3ζ/rB6C ****$p<0.0001$. **(B)** Stimulation of GFP production in 2B4T reporter cells by rB75KN variants, quantified using flow cytometry. The percentage of GFP-positive cells was calculated and normalised against cells stimulated with anti-CD3. Mean ± s.d. of $n = 3$ independent experiments, with two-way ANOVA where 2B4T/rB75KN vs 2B4T.LILRB3CD3ζ/rB75KN ****$p<0.0001$ and 2B4T/rB75KN^{L721A} vs 2B4T.LILRB3CD3ζ/rB75KN^{L721A} **$p=0.0016$.

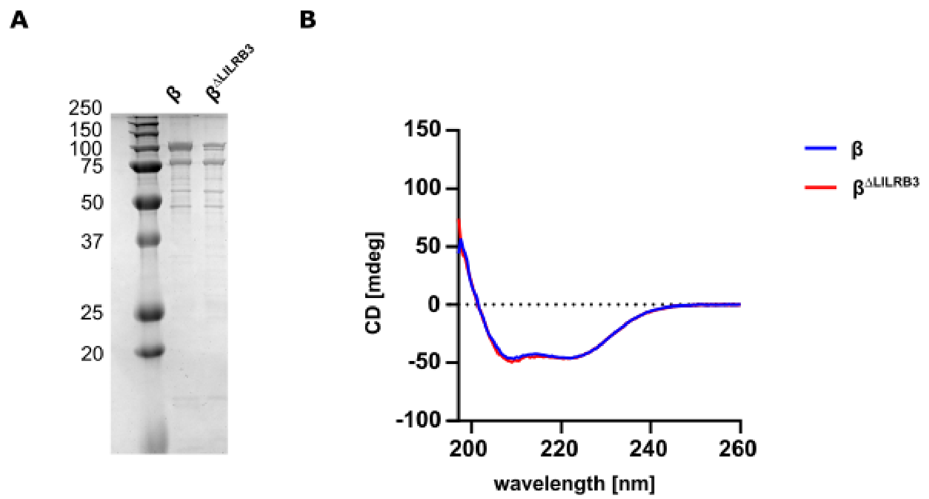

Supplementary Figure 11. Generation of wildtype and mutated rβ protein. (A) SDS-PAGE showing purified rβ and $r\beta^{\Delta LILRB3}$ variants. **(B)** Circular dichroism spectroscopy analysis of rβ and $r\beta^{\Delta LILRB3}$ variants. Data are shown scaled to the ellipticity at 222 nm to compare secondary structural integrity between the wildtype and mutated proteins.

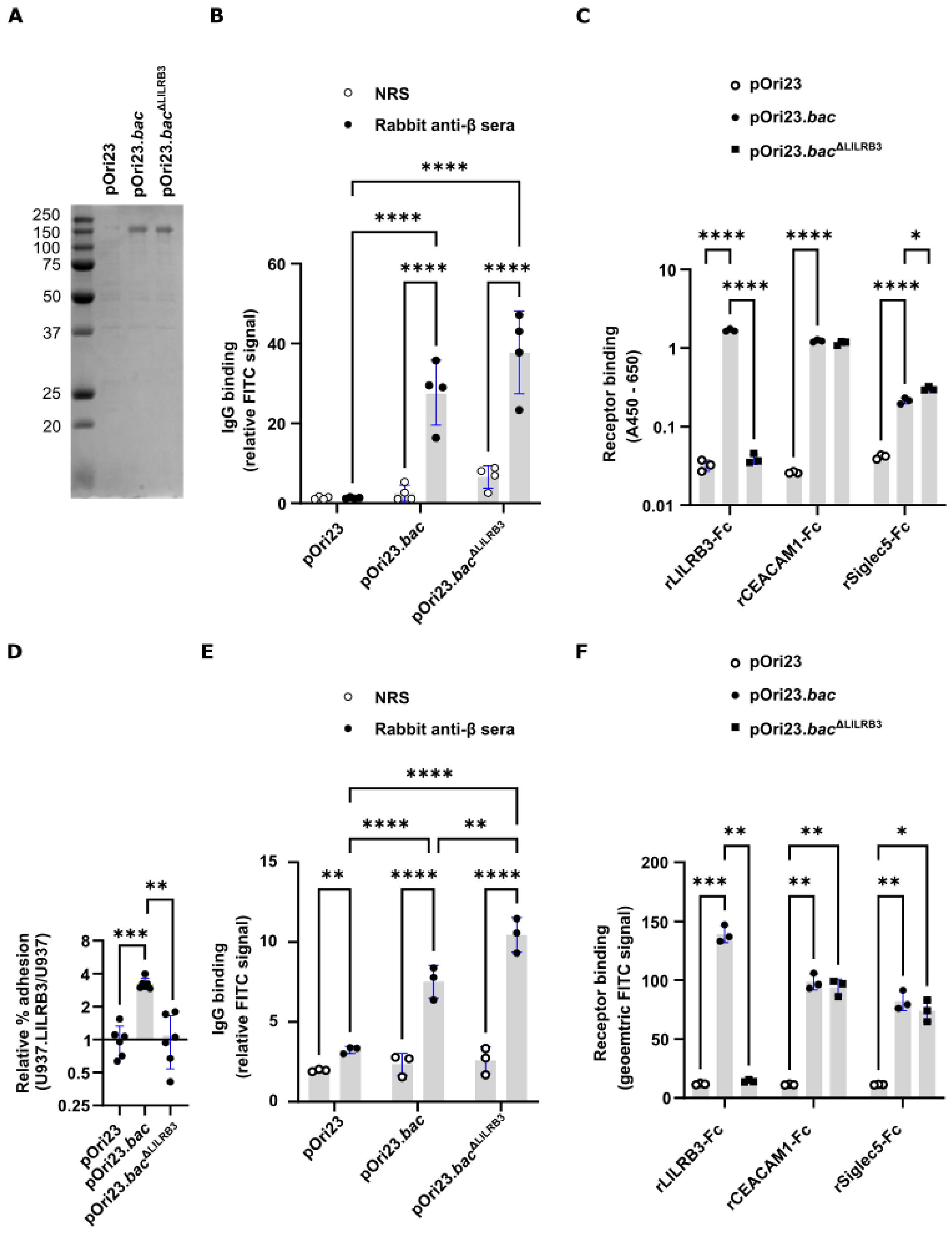

Supplementary Figure 12: Generation of bacteria expressing wildtype and mutated β protein. (A) SDS-PAGE analysis of total lysates from *L. lactis* strains, showing expression of β proteins from pOri23 vectors. **(B)** Surface expression of β protein by *L. lactis* strains measured

using rabbit anti-β sera, quantified using flow cytometry. Preimmune rabbit sera was used as a control. Mean ± s.d. of $n = 4$ independent experiments, with two-way ANOVA where anti-β/pOri23 vs anti-β/pOri23.*bac* ****$p<0.0001$, NRS/pOri23.*bac* vs anti-β/pOri23.*bac* ****$p<0.0001$, NRS/pOri23.$bac^{\Delta LILRB3}$ vs anti-β/pOri23.$bac^{\Delta LILRB3}$ ****$p<0.0001$. **(C)** Binding of rLILRB3-Fc, rCEACAM1-Fc and rSiglec5-Fc to *L. lactis* strains, quantified by ELISA assay. The mean ± s.d. of $n = 3$ independent experiments, with two-way RM ANOVA, where pOri23/rLILRB3 vs pOri23.*bac*/rLILRB3 ****$p<0.0001$, pOri23.*bac*/rLILRB3 vs pOri23.$bac^{\Delta LILRB3}$/rLILRB3 ****$p<0.0001$, where pOri23/rCEACAM1 vs pOri23.*bac*/rCEACAM1 ****$p<0.0001$, pOri23/rSiglec5 vs pOri23.*bac*/rSiglec5 ****$p<0.0001$, pOri23.*bac*/rSiglec5 vs pOri23.$bac^{\Delta LILRB3}$/rSiglec5 *$p=0.05$. **(D)** Binding of FITC-labelled *L. lactis* strains to LILRB3-expressing or control U937 transfectants, quantified using flow cytometry. Mean ± s.d. of $n = 6$ independent experiments, with one-way RM ANOVA, where pOri23 vs pOri23.*bac* ***$p=0.003$, and pOri23.*bac* vs pOri23.$bac^{\Delta LILRB3}$ **$p=0.0035$. **(E)** Surface expression of β protein by *S. agalactiae* strains measured using rabbit anti-β sera, quantified using flow cytometry. Preimmune rabbit sera was used as a control. Mean ± s.d. of $n = 3$ independent experiments, with two-way ANOVA where NRS/pOri23 vs anti-β/pOri23 **$p=0.0036$, NRS/pOri23.*bac* vs anti-β/pOri23.*bac* ****$p<0.0001$, NRS/pOri23.$bac^{\Delta LILRB3}$ vs anti-β/pOri23.$bac^{\Delta LILRB3}$ ****$p<0.0001$, anti-β/pOri23 vs anti-β/pOri23.*bac* ****$p<0.0001$, anti-β/pOri23 vs anti-β/pOri23.$bac^{\Delta LILRB3}$ ****$p<0.0001$, and anti-β/pOri23.*bac* vs anti-β/pOri23.$bac^{\Delta LILRB3}$ **$p=0.0013$. **(F)** Binding of rLILRB3-Fc, rCEACAM1-Fc and rSiglec5-Fc to *S. agalactiae* A909Δ*bac* strains, quantified by flow cytometry assay. The mean ± s.d. of $n = 3$ independent experiments, with two-way RM ANOVA, where pOri23/rLILRB3 vs pOri23.*bac*/rLILRB3 ***$p=0.0006$, pOri23.*bac*/rLILRB3 vs pOri23.$bac^{\Delta LILRB3}$/rLILRB3 **$p=0.0017$, pOri23/rCEACAM1 vs pOri23.*bac*/rCEACAM1 **$p=0.0032$, pOri23/rCEACAM1 vs pOri23.$bac^{\Delta LILRB3}$/rCEACAM1 **$p=0.0035$, pOri23/rSiglec5 vs pOri23.*bac*/rSiglec5 **$p=0.0078$, and pOri23/rSiglec5 vs pOri23.$bac^{\Delta LILRB3}$/rSiglec5 *$p=0.012$.

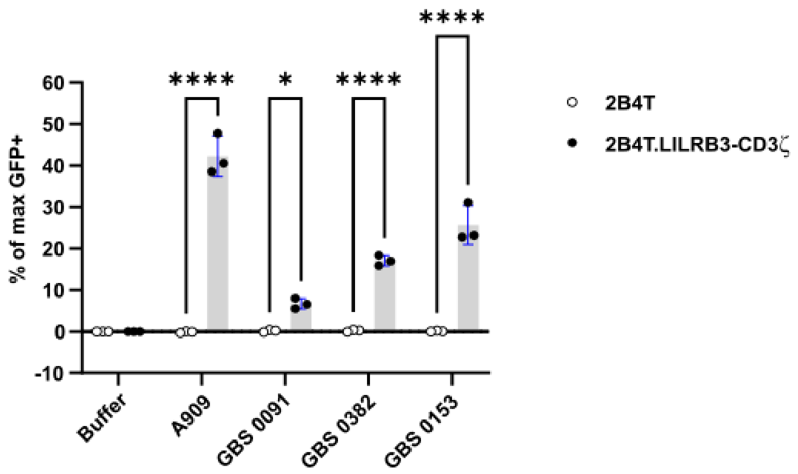

Supplementary Figure 13: Clinical *S. agalactiae* isolates trigger LILRB3 reporter cells. Stimulation of GFP production in 2B4T reporter cells after incubation with clinical *S. agalactiae* isolates, quantified using flow cytometry. The percentage of GFP-positive cells was calculated and normalised against cells stimulated with anti-CD3. Mean ± s.d. of *n* = 3 independent experiments, with two-way ANOVA with Šídáks multiple comparison test, where 2B4T/A909 vs 2B4T.LILRB3-CD3ζ/A909 ****$p<0.0001$, 2B4T/GBS0091 vs 2B4T.LILRB3-CD3ζ/GBS0091 *$p<0.0279$, 2B4T/GBS0382 vs 2B4T.LILRB3-CD3ζ/GBS0382 ****$p<0.0001$, 2B4T/GBS0153 vs 2B4T.LILRB3-CD3ζ/GBS0153 ****$p<0.0001$.

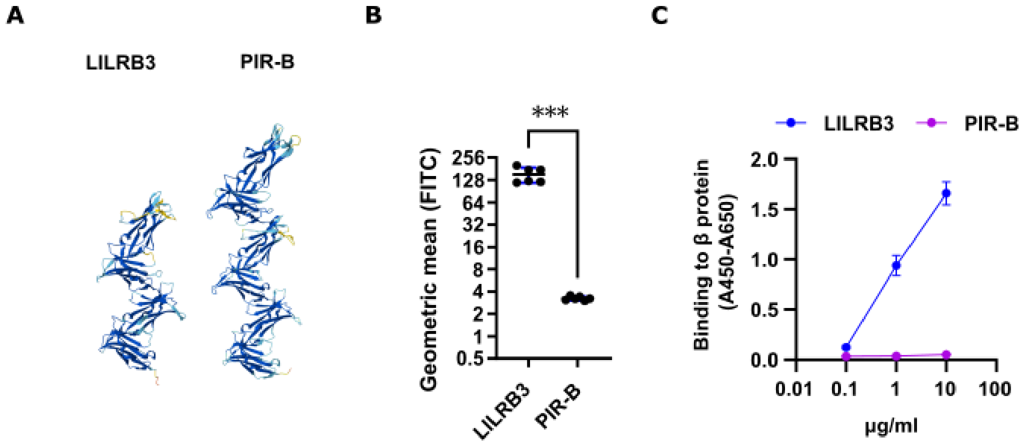

Supplementary Figure 14: *S. agalactiae* does not interact with mouse PIR-B. (A) AlphaFold prediction for human LILRB3 (Accession: AF-O75022-F1-v4) and mouse PIR-B (Accession: AF-P97484-F1-v4). The prediction is coloured by model confidence band, where blue = very high confidence (pLDDT > 90), cyan = confident (90 > pLDDT > 70), yellow = low confidence (70 > pLDDT > 50) and very low = orange (pLDDT < 50). **(B)** Binding of rLILRB3-His or rPIR-B-His (10 μg/ml) to *S. agalactiae* A909, quantified using flow cytometry. Data is displayed as relative to rLILRB3- or rPIR-B binding compared to secondary antibody control stains. Mean ± s.d. of $n = 6$ independent experiments, with student *t*-test, where ***p=0.0002. **(C)** Binding of rLILRB3-His or rPIR-B-His to purified β protein, quantified using ELISA. Mean ± s.d. of $n = 3$ independent experiments.

Supplementary Table 1: Interaction Parameters for β protein Domains and LILRB3[#]

Analyte	Ligand	ka ($10^5 M^{-1}s^{-1}$)	kd ($10^{-2}s^{-1}$)	KD (nM)	Rmax (RU)
LILRB3	B6C	3.1±0.1	12.4±1.1	400.0±1.5	755.6±5.2
LILRB3	B75KN	3.8±0.1	5.0±0.1	131.0±0.8	755.9±0.8
LILRB2	B6C	-	-	-	-
LILRB2	B75KN	-	-	-	-

[#]For the experiments presented in Fig. 3. Ligand immobilisation levels for B6C and B75KN were 590.5 and 951.6 RU, respectively.

Supplementary Table 2: Interaction Parameters for β protein Domains and LILRB3[$]

Analyte	Ligand	ka ($10^5 M^{-1}s^{-1}$)	kd ($10^{-2}s^{-1}$)	KD (nM)	Rmax (RU)
LILRB3	B6C	3.2±0.2	9.1±0.1	286.6±10.9	78.9±3.7
LILRB3	B75KN-C	4.8±0.4	8.7±1.8	181.7±22.3	60.4±0.1
LILRB3-D3D4	B6C	2.5±0.7	12.1±1.0	500.2±109.7	75.0±5.9
LILRB3-D3D4	B75KN-C	3.4±1.6	10.1±2.8	307.4±64.3	53.0±2.9

[$]For the experiments presented in Fig. 4. Ligand immobilisation levels for B6C and B75KN-C were 190 and 154 RU, respectively.

Supplementary Table 3: Interaction Parameters for β protein Domains and LILRB3*

Analyte	Ligand	KD (nM)	Rmax (RU)
B6C	LILRB3-biotin	223.1±14.1	53.9±1.9
$B6C^{K368A}$	LILRB3-biotin	4170.7±275.4	39.2±1.6
$B6C^{R372A}$	LILRB3-biotin	3331.7±293.3	42.3±1.7
$B6C^{E375A}$	LILRB3-biotin	551.8±49.2	48.8±2.8
B75KN-C	LILRB3-biotin	94.6±7.2	29.9±0.9
$B75KN\text{-}C^{L721A}$	LILRB3-biotin	9244.7±301.6	26.1±1.3
$B75KN\text{-}C^{D722A}$	LILRB3-biotin	3364.3±175.8	33.5±1.4
$B75KN\text{-}C^{F740A}$	LILRB3-biotin	2829.3±130.4	32.9±1.4

*For the experiments presented in Supplementary Fig. 6. Ligand capture levels for LILRB3-biotin were approximately 900 RU across all experimental flow cells.
