# Supplementary material for: Bacterial targeting of the neutrophil inhibitory receptor LILRB3 to evade antibody immunity
Source: Nat Commun. 2026 Jun 11;17:7463. doi: 10.1038/s41467-026-74098-6 (PMC13408158; doi:10.1038/s41467-026-74098-6)
Supplement: Supplementary file 2 — Description of Additional Supplementary File [file 41467_2026_74098_MOESM2_ESM.pdf]

Description of Additional Supplementary Files

Supplementary Data 1: SAXS Samples, Data Collection, Analysis, and Evaluation.

Supplementary Data 2: Bacterial strains used in this study

Supplementary Data 3: Sequence of synthetic DNA used in this study

Supplementary Data 4: cDNA vectors and amplification primers
