# Supplementary material for: Bacterial targeting of the neutrophil inhibitory receptor LILRB3 to evade antibody immunity
Source: Nat Commun. 2026 Jun 11;17:7463. doi: 10.1038/s41467-026-74098-6 (PMC13408158; doi:10.1038/s41467-026-74098-6)
Supplement: Supplementary file 7 — Reporting summary [file 41467_2026_74098_MOESM7_ESM.pdf]

nature portfolio

Corresponding author(s): Alex McCarthy
Last updated by author(s): 2025-22-10

Reporting Summary

Nature Portfolio wishes to improve the reproducibility of the work that we publish. This form provides structure for consistency and transparency in reporting. For further information on Nature Portfolio policies, see our Editorial Policies and the Editorial Policy Checklist.

Statistics

For all statistical analyses, confirm that the following items are present in the figure legend, table legend, main text, or Methods section.

n/a	Confirmed	
☐	☒	The exact sample size (*n*) for each experimental group/condition, given as a discrete number and unit of measurement
☐	☒	A statement on whether measurements were taken from distinct samples or whether the same sample was measured repeatedly
☐	☒	The statistical test(s) used AND whether they are one- or two-sided<br>*Only common tests should be described solely by name; describe more complex techniques in the Methods section.*
☒	☐	A description of all covariates tested
☐	☒	A description of any assumptions or corrections, such as tests of normality and adjustment for multiple comparisons
☐	☒	A full description of the statistical parameters including central tendency (e.g. means) or other basic estimates (e.g. regression coefficient) AND variation (e.g. standard deviation) or associated estimates of uncertainty (e.g. confidence intervals)
☒	☐	For null hypothesis testing, the test statistic (e.g. *F*, *t*, *r*) with confidence intervals, effect sizes, degrees of freedom and *P* value noted<br>*Give P values as exact values whenever suitable.*
☒	☐	For Bayesian analysis, information on the choice of priors and Markov chain Monte Carlo settings
☒	☐	For hierarchical and complex designs, identification of the appropriate level for tests and full reporting of outcomes
☒	☐	Estimates of effect sizes (e.g. Cohen's *d*, Pearson's *r*), indicating how they were calculated

Our web collection on statistics for biologists contains articles on many of the points above.

Software and code

Policy information about availability of computer code

Data collection	No software was used
Data analysis	Analysis of Streptococcus agalactiae genomes for carriage of bac gene was performed using the PubMLST dataset and analysis software. Flow cytometry data was analysed in FlowJo v10.8. Data was analysed in GraphPad Prism 8.4.3.<br><br>Crystallography data was processed and indexed and scaled using HKL-2000 software. Phase information was obtained through the AutoSol program of the PHENIX software suite. Automated chain tracing was performed with AutoBuild within PHENIX software suite. Manual modelling was performed in COOT. SAXS data processing and analysis was performed using MultiFoXS and DENSS.

For manuscripts utilizing custom algorithms or software that are central to the research but not yet described in published literature, software must be made available to editors and reviewers. We strongly encourage code deposition in a community repository (e.g. GitHub). See the Nature Portfolio guidelines for submitting code & software for further information.

Data

Policy information about availability of data

All manuscripts must include a data availability statement. This statement should provide the following information, where applicable:

- Accession codes, unique identifiers, or web links for publicly available datasets
- A description of any restrictions on data availability
- For clinical datasets or third party data, please ensure that the statement adheres to our policy

All data supporting the findings of this study are available within the paper or from the corresponding author upon request. Structure of B6C (PDB: 9MN2) is deposited in Protein Data base (PDB). No original code is used. Sensorgram data sets from SPR were analyzed using Biacore T-200 Evaluation software v3.2

Research involving human participants, their data, or biological material

Policy information about studies with human participants or human data. See also policy information about sex, gender (identity/presentation), and sexual orientation and race, ethnicity and racism.

Reporting on sex and gender	Human blood was obtained from healthy donors for studies using purified neutrophils and human whole blood. Data reported related to both sexes and all genders. Neither sex or gender were considered during design. All samples were collected after receiving signed informed consent from all participants.
Reporting on race, ethnicity, or other socially relevant groupings	Socially constructed or socially relevant categorization variables were not used in the manuscript.
Population characteristics	All individuals were healthy. No other data was collected.
Recruitment	Donation of blood was voluntary and participants were volunteers from the Institute directly involved in the experiment. Recruitment was via word of mouth or institute emailing. Signed consent was collected. No bias on gender.
Ethics oversight	Human blood was obtained from healthy donors, approved by the Regional Ethics Committee and Imperial College Healthcare NHS Trust Tissue Bank (Regional Ethics Committee approval no. 17/WA/0161, Imperial College Healthcare Tissue Bank Human Tissue Authority license no. 12275, and Imperial College Research Ethics Committee no. 19IC5166).

Note that full information on the approval of the study protocol must also be provided in the manuscript.

Field-specific reporting

Please select the one below that is the best fit for your research. If you are not sure, read the appropriate sections before making your selection.

☒ Life sciences ☐ Behavioural & social sciences ☐ Ecological, evolutionary & environmental sciences

For a reference copy of the document with all sections, see nature.com/documents/nr-reporting-summary-flat.pdf

Life sciences study design

All studies must disclose on these points even when the disclosure is negative.

Sample size	Sample sizes were not predetermined based on statistical methods, but were chosen according to the standards in the field - at least 3 independent biological replicates. This typically generated sufficient data for statistical analysis.
Data exclusions	No data were excluded from analysis
Replication	Numbers of experimental replicates are stated in each figure legend. Reported results were consistently replicated across multiple experiments with all replicates generating similar results.
Randomization	No randomization was necessary as experiments were performed with appropriate controls. Randomization is not generally used in this field
Blinding	Investigators were not blinded. Blinding during analysis was not necessary because the results are quantitative and did not require subjective judgment or interpretation. Blinding is not typically used in the field.

Reporting for specific materials, systems and methods

We require information from authors about some types of materials, experimental systems and methods used in many studies. Here, indicate whether each material, system or method listed is relevant to your study. If you are not sure if a list item applies to your research, read the appropriate section before selecting a response.

Materials & experimental systems

n/a	Involved in the study	
☐	☒	Antibodies
☐	☒	Eukaryotic cell lines
☒	☐	Palaeontology and archaeology
☒	☐	Animals and other organisms
☒	☐	Clinical data
☒	☐	Dual use research of concern
☒	☐	Plants

Methods

n/a	Involved in the study	
☒	☐	ChIP-seq
☐	☒	Flow cytometry
☒	☐	MRI-based neuroimaging

Antibodies

Antibodies used	anti-human LILRB3, R&D Biosystems, catalog #MAB1806, clone 222821<br>Isotype control mouse IgG2a from Merck Millipore #PP102<br>Alexa Fluor® 647 AffiniPure® F(ab')$_2$ Fragment Goat Anti-Mouse IgG (H+L) from Jackson ImmunoResearch, catalog #115-606-146, polyclonal<br>PE-conjugated anti-human FcγRIIIb from BD Biosciences Catalog #555407, Clone 3G8<br>PE-conjugated anti-human FcγRIIa from ThermoFisher Scientific Catalog #CD3204, Clone AT10<br>PE-congugated anti-human FcγRI from Biolegend, Catalog #305006, Clone 10.1<br>PE-conjugated anti-human FcαRI from BioRad Catalog #MCA1824PE, clone MIP8a<br>FITC-conjugated anti-His from ThermoFisher Catalog #MA1-81891 clone AD1.1.10<br>FITC-conjugated anti-human-IgG from SouthernBiotech Catalog #2040-02, polyclonal<br>HRP-conjugated goat anti-human-IgG from ThermoFisher Catalog # A18805<br>HRP-conjugated goat anti-mouse-IgG from ThermoFisher Catalog # 31430<br>anti-FcγRIIa from STEMCELL Technologies Catalog #60012 clone IV.3<br>anti-FcαRI from Bio-Rad Catalog #MCA1824 clone MIP8a<br>mouse isotype IgG1 R&D biosystems, catalog #MAB002 ,clone 11711<br>HRP-conjugated anti-6xHis from BioLegend Catalog #652504
Validation	Validation statements on manufacturers websites.

Eukaryotic cell lines

Policy information about cell lines and Sex and Gender in Research

Cell line source(s)	U937 cells are a human monocytic cell line, previously published in PMID: 31915259. Original source of parental cell line = Jos van Strijp, UMC Utrecht.<br>THP-1 cells are a human monocytic cell line. Original source of parental cell line = Linde Meyaard, UMC Utrecht.<br>2B4 NFAT-GFP T are a mouse T cell line, previously published in PMID: 31915259. Original source of parental cell line = Linde Meyaard, UMC Utrecht.<br>EXPI293f cells received from Thermo Fisher Scientific.
Authentication	No authentication.
Mycoplasma contamination	U937, 2B4T, THP-1 and EXPI293f cells were tested for mycoplasma contamination.
Commonly misidentified lines<br>(See ICLAC register)	No commonly misidentified lines were used in this study

Plants

Seed stocks	N/A
Novel plant genotypes	N/A
Authentication	N/A

Flow Cytometry

Plots

Confirm that:

☒ The axis labels state the marker and fluorochrome used (e.g. CD4-FITC).

☒ The axis scales are clearly visible. Include numbers along axes only for bottom left plot of group (a 'group' is an analysis of identical markers).

☒ All plots are contour plots with outliers or pseudocolor plots.

☒ A numerical value for number of cells or percentage (with statistics) is provided.

Methodology

Sample preparation	Bacterial cells prepared by culture in appropriate media. U937, THP-1 and 2B4T cells cultured as detailed in methods.<br>Neutrophils purified as detailed in methods.
Instrument	Data collected on BD FACSCalibur™ Flow Cytometer - BD Biosciences, or Amnis CellStream® Benchtop Flow Cytometer from Luminex
Software	Data collected BD FACStation™ software or CellStream™ Acquisition and Analysis Software<br>Data analysed FlowJo v10.8.0.
Cell population abundance	Cells were not sorted.
Gating strategy	Bacteria populations gated on FSC/SSC.<br>Neutrophils gated on FSC/SSC.<br>U937, THP-1, 2B4T cells gated on FSC/SSC.

☒ Tick this box to confirm that a figure exemplifying the gating strategy is provided in the Supplementary Information.
