# Supplementary material for: Bacterial targeting of the neutrophil inhibitory receptor LILRB3 to evade antibody immunity
Source: Nat Commun. 2026 Jun 11;17:7463. doi: 10.1038/s41467-026-74098-6 (PMC13408158; doi:10.1038/s41467-026-74098-6)
Supplement: Supplementary file 8 — Transparent Peer Review File [file 41467_2026_74098_MOESM8_ESM.pdf]

nature portfolio

Peer Review File

Bacterial targeting of the neutrophil inhibitory receptor LILRB3 to evade antibody immunity

Corresponding Author: Dr Alex McCarthy

This file contains all reviewer reports in order by version, followed by all author rebuttals in order by version.

Version 0:

Reviewer comments:

Reviewer #1

(Remarks to the Author)
Rumpret et al. report that interaction between the surface-localized β protein of S. agalactiae (Group B Streptococcus) and human LILRB3, an inhibitory immune receptor expressed by neutrophils, antagonizes Fc receptor-mediated antibacterial responses. This is an important result based on an extensive and varied set of data. The paper is generally strong although there are some weaknesses.

The authors demonstrate that β protein and LILRB3 interact directly, and identify the particular β protein domains (B6C and B75K) and LILRB3 regions (D3-loop-D4) responsible for the interaction. They further show that the interaction activates signaling downstream of LILRB3 in a T cell reporter system, and decreases the oxidative burst triggered by engagement of FcRIIa in human neutrophils.

The most compelling evidence that this interaction is biologically significant comes from a neutrophil killing assay (Fig. 7C). The authors show that loss of β protein results in a decrease in survival of GBS from slightly less than 100% to about 75%, which is not a major effect but a statistically significant one nevertheless. This loss in GBS survival can be complemented by exogenous expression of β protein from a plasmid, but importantly, not by a β protein mutant (βΔLILRB3) containing five Ala-substitutions that eliminate binding to LILRB3. However, a major issue with this experiment is that the authors do not validate βΔLILRB3 for surface expression and specific loss of function GBS. This sort of validation is indeed carried out but in L. lactis (Supplementary Figure 8). The authors need to carry out these validation experiments in the relevant biological context, i.e., in GBS. There are similar issues with the rigor of the data in the some of the biochemical experiments when it comes to validating negative results (see below).

The authors also show a correlation between β protein expression and GBS lineages that cause invasive disease in older adults. They infer from this that “targeting inhibitory receptors most likely provides an immune evasion mechanism when antibody responses are waning, such as in immunocompromised or elderly individuals.” Why would this be? It seems more logical that targeting inhibitory receptors would be need when antibody responses are strong. While the correlation exists, the implications require further consideration.

1. The abstract (and other sections) state “β and LILRB3 interactions suppressed the Fc receptor-mediated antibacterial responses such as respiratory burst and phagocytosis …” However, there are no experiments examining phagocytosis per se.
2. The authors determine binding affinities for rLILRB3 for β-rB6C and β-rB75KN in Figure 3. It seems from Figure 6C that the binding affinity of these individual β protein domains is much lower than that of a fragment that contains both these domains (B6C-IgI3-B75KN). Is this true, and if so, why? The authors should comment on this.
3. Why are data reported as geometric mean in Figure 2A but % FITC+ in 2B?
4. In Figures 4B and 4C, the y-axes are designated “Binding to” rB6C and rB75KN, respectively. What is specifically being reported? Why are the values so different between 4B (spanning 0–30) and 4C (spanning 20-100)? The same question applies to Figures 5B and 5C, and Supplementary Figures 3C and 3D.
5. LILRB3 D2-D3-D4 appears to be a weaker binder than D1-D2-D3-D4 and D3-D4 (in Figure 4). Do the authors have an explanation for this?
6. A 2Fo-Fc map is phase biased and thus not meaningful as an indicator of correctness (Supplementary Figure 4A). The authors should instead show an 2Fo-Fc omit map or composite omit map of a representative portion of the electron density

(and indicate which regions are being omitted and what sigma level is being depicted).
7. L. 259, “We then generated ab initio density envelopes, which matched well with the computational models obtained for each complex [Supplementary Fig. 5G-H].” This reviewer disagrees with this qualitative judgment. The envelopes have a great deal of empty space, not occupied by the model. These envelopes seem to be quite poor matches.
8. The authors identify a number of amino acids in β protein and LILRB3 as interactors based on structural models. They should specify for each amino acid which partner amino acids are predicted to be contacted.
9. Based on negative results from binding experiments in Figure 5, the authors infer that a set of amino acids in β protein contact LILRB3 and vice versa. This is unjustified. The authors do not establish that the negative results are not due to nonspecific loss in structure or stability, as compared to a specific effect on binding. This could be assessed, for example, through comparison of circular dichroism spectra to establish structural integrity and denaturation profiles (increasing temperature or denaturant concentration, as monitored by circular dichroism) for stability. The same issue applies to the multiple mutation βΔLILRB3 construct (in Figure 6).
10. A general problem exists in ELISAs that have different components affixed to wells. For example, the data in Figures 5B and 5E can be explained by N287A, Q289A, etc. not adhering to wells as well as wild-type protein. The authors need to control for this.
11. The data purported to show that a single β protein molecule can bind two molecules of LILRB3 (and thus cause clustering for signaling) are not convincing. The result that a 2:1 stoichiometry inhibits better than 1:1 may simply reflect greater fractional occupancy at higher stoichiometry. The SAXS data shown in Supplementary Table 3 are more convincing. However, an even more convincing experiment for the authors’ point about signaling would be the following. If they were able to show that mutations in B6C knock out binding specifically and others in B75KN do likewise (i.e., no effect on structure or stability), then intact β protein containing only the B6C mutation or only the B75KN mutation should still bind LILRB3 (1:1 stoichiometry) but no longer be able to signal (lacking 2:1 stoichiometry).
12. SDS-PAGE is shown to establish the purity of LILRB3 constructs in Supp. Fig. 3A, but equivalent data are not provided for β protein variants. They should be.

Minor Comments
1. In Figure 2G and other figures with Amplex Red data, it is difficult to discern individual curves, as the data points are large and obscure each other.
2. The designation “B6C-IgI3-B75KN” appears without an introduction. Readers who are not intimately familiar with β protein domains will be confused by this, including this reviewer. This term should be explained.
3. In descriptions of ELISAs in the main text or figure legends, it would be useful to designate which component is affixed and how the soluble component is detected.
4. In figures showing protein structures, it would be useful to label the N- and C-termini (by that designation or by numbers).
5. There are various typos throughout. For example, L. 130 “signifcantly". Careful inspection of the Word spell checker is warranted.
6. L. 178, “Similar inhibitory effects were identified when we stimulated FcαRI (CD89) on neutrophils [Supplementary Fig. 2A and 2B].” The authors presumably mean Supplementary Figures 1D and 1E.
7. In Supplementary Figure 4B, the authors should be explicit regarding which color corresponds to which model.
8. In Figures 5C and 5F, it is not clear which proteins are affixed to ELISA wells.
9. The source of human serum is not indicated.
10. L. 332, “Indeed, both coated and soluble rβ, but not coated and soluble rβΔLILRB3, stimulated GFP expression in LILRB3-CD3ζ reporter cells but not control cells [Fig. 6D].” This reviewer assumes coated means on dynabeads. Is that correct? It should be explicitly stated.
11. In Figure 7C, it appears that wild-type A909 is not killed by human neutrophils within the timeframe of the experiment (about 100% survival). Is this expected? The Materials and Methods states: “At designated time points after incubation at 37°C, samples were lysed in 0.3% saponin, serially diluted into sterile PBS and plated onto TH agar plates. After culturing at 37°C for 1 hour, the % survival was calculated as CFU after incubation with neutrophils relative to CFU of the inoculum.” What were these designated time points? Is this a 1 hour experiment? Is that what “culturing” refers to?
12. The Materials and Methods section appears to be lacking a description of the ELISA experiment in Supplementary Figure 9C in its use of whole bacteria.
13. Figure 9 has details in the legend (e.g., Syk, SHP-1, etc.) that are not depicted in the figure.
14. L. 503, “Since other S. aureus …” What does “other” mean in this context?
15. In Table 1, the extents of the highest resolution shell should be stated. Statistics for the highest resolution shell are missing in the refinement section. How many reflections were in the work and free sets?
16. L. 745, “Friedel pairs” should be “Bijvoet pairs.”
17. The authors should double check their references and bibliography. For example, L. 159 references (Zhao et al., 2020b), which shows up twice in the numbered bibliography (ref. 18 and 83).
18. The 2B4 T cell reporter system appears to be murine rather than human in origin, although the chain of references to ferret this out is long. In any case, it is worthwhile clarifying this.

Reviewer #2

(Remarks to the Author)
This is an interesting and important paper elucidating the function of inhibitory LILRB3 on bacterial infection. Because function of LILRB3 on bacterial infection was not known, their findings that LILRB3 binds to β protein of S. agalactiae is quite important. They validated the interaction between LILRB3 and β protein not only by using recombinant protein but also by structual analysis of LILRB3. Furthermore, the predicted binding sites were confirmed by point mutations. In addition, they

have demonstrated that LILRB3 signaling downregulates the killing activity of neutrophils against β protein-expressing bacteria. Their studies are well organaized and the results are clear. The followings are point that may improve the paper.

1. The authors demonstrated that some of clinical isolate of S. agalactiae express β protein and bind to LILRB3. These findings are important to understand the pathophysiological function of LILRB3 and β protein interaction. Do these isolates expressing β protein also stimulate LILRB3-reporter cells?

2. In the discussion, the authors described that binding of LILRB3 to E. coli and S. aureus has been already publised in reference 39. It looks inconsistent to the authors' discription that this is the first report showing the interaction of ILRB3 and bacteria. However, reference 39 is the studies of mouse PIR-B not human LILRB3. Although PIR-B has homology to human LILRB3, the amino acid homology is not so high. Therefore, they shoud describe precisely the differene between PIR-B and LILRB3 as well as difference from this paper.

3. In the last part, the authors conclude that inhibitory receptors are also involved in dampening innate PRR-dependent immune responses to bacteria. However, the authors did not analyzed PRR-dependent reponse of neutrophils and it is unclear whether inhibitory receptors are involved in PRR-dependent immune response. These discription shold be revised.

4. Although the authors suggest that LILRB3 is important to immune response to variety of bacteria. If so, various types of bacteria also should use LILRB3 for immune evasion. Why only S. agalactiae use LILRB3 for immune evasion?

5. For the analysis of interaction between LILRB3 and β protein on bacteria, some experiments were done by flow cytometry but other were done with ELISA. What is the difference?

6. There are some LILRB3CD3ζ. It should be LILRB3-CD3ζ.

Reviewer #3

(Remarks to the Author)
This paper by Rumpret et al., reveals a previously unrecognized immune evasion strategy where the Streptococcus agalatiae (GBS) surface β protein directly targets a the neutrophil inhibitory receptor LILRB3 to suppress antibody-mediated immunity.

The study is a tour de force in molecular microbiology, neutrophil functional assays, protein chemistry and structural biology.

Antibody-mediated immunity is crucial for defending against bacterial pathogens, primarily through Fc receptor (FcR)-mediated phagocytosis and bacterial killing by neutrophils. Inhibitory immune receptors, such as LILRB3, regulate immune cell activation, but it has been unclear whether bacteria directly target these receptors to evade antibody-driven responses.

By using both loss of function and gain of function experiments, based on bac knockouts lacking β protein expression in GBS, or heterologous expression of β protein in Lactococcus lactis, it is very clear that β protein is required and sufficient to interact with purified LILRB3. Furthermore, it is shown GBS expressing β protein binds to cells expressing LILRB3 and can crosslink the receptor leading to inhibitory signals and reduced Fc-receptor mediated oxidative burst.

Further mapping of the interaction indicate that two distinct domains of β are responsible for high affinity binding to. LILRB3. Furthermore, these two domains are sufficient to block FcR-mediated ROS production in neutrophils. Additional mapping on the host side revealed that Ig domains D3 to D4 in LILRB3 are bound by the two β domains.

Further structural analysis eluciated which amino acids in the two β domains that are involved in binding of LILRB3 but not of some of the other know ligands for β.

Analysis of clinical GBS isolates revealed that β expressing strains are associated with lineages that cause disease in non-pregnant adults. Furthermore, β can alone crosslink LILRB3 leading to inhibitory signals, and a bac knockout GBS transcomplemented with bac encoding β only interacting with LILRB3 still impairs killing by neutrophils indicating that interaction with LILRB3 is a major contributor to this type of immune evasion.

Broader implications of the study is that bacterial pathogens can directly target human inhibitory receptors to subvert antibody-dependent phagocyte responses. Understanding these interactions is critical for designing effective vaccines and antibody-based therapies, especially for vulnerable populations.

The study is extremely well conceived and distinctly presented both in text and figures. I'm hard pressed to find any major flaws, but the anti-phosphorylation Western blots are of lower quality than the rest of the data. Not a major problem, since the trends seen in these fits the other functional data, but it would be nice to see more convincing Westerns or other phosphorylation assays.

Version 1:

Reviewer comments:

Reviewer #1

(Remarks to the Author)
The revision by Rumpret et al. has suitably addressed almost all of my prior concerns. Most importantly, the authors have carried out controls for expression and function of wild-type and ΔLILRB3 β protein as expressed by S. agalactiae (Figs. S12E and F). The figures show comparable expression, but curiously the authors did not carry out statistical comparison between the two (i.e., pOri23.bac vs. pOri23bac(ΔLILRB3); they should. They also find that ΔLILRB3 β protein maintains CECAM- and Siglec5-binding, suggesting that functional effects on LILR3 binding are not due to nonspecific structural alteration or loss of stability.

The major point that the authors seem to have ignored in the paper and rebuttal is evaluation of the stability of mutant proteins. The authors present circular dichroism (CD) data at a single temperature, but the suggestion to carry out a temperature or denaturant melt, as monitored by CD, to assay stability was not carried out or commented on. A Materials and Methods section for the CD experiments is lacking. There is no indication of what temperature these experiments were done and how many independent times they were done. The overall concern regarding stability would be abated if in vitro binding experiments using the mutant proteins were carried out at the same temperature as the CD analysis.

Nevertheless, the CD spectra of wild-type and mutant LILRB3 are clearly different (Fig. 7B), most tellingly having different zero-crossings. This indicates that the secondary structure differs between wild-type and mutants, and the secondary structure also appears to differ among mutants. Therefore, the statement that wild-type and mutant LILRB3 “displayed similar folding structure” (L. 281) is unjustified. Similarly, rβ and rβ(ΔLILRB3) appear to differ in structure (Fig. S11B) and perhaps wild-type and L721A (Fig. S8G), but it is difficult to gauge given the lack of details and statistics. In the latter two cases, similar zero-crossings occur for wild-type and mutant, and so differences in spectra may be due to errors in protein concentration determination rather than bona fide structural alteration.

A smaller point is that Figure S4 appears to show density only around atoms. This reviewer does not suspect that the structure is incorrect, but for rigor, entire density maps should be shown, and for clarity, around some ‘representative’ region of the map and protein rather than the entire protein. It is impossible to evaluate the quality of the map in the current figures. The designation “1.2s” should be replaced by “1.2σ.” The issue of phase bias is well encapsulated by Kathryn Cowtan’s famous duck and cat examples of Fourier Transforms (http://www.ysbl.york.ac.uk/~cowtan/fourier/magic.html). This point is also in Berhard Rupp’s Biomolecular Crystallography textbook, in which entirely random amplitudes but with ‘correct’ phases are shown to yield an interpretable map, but not vice versa. This reviewer has no objection to showing both 2Fo-Fc and omit 2Fo-Fc maps, but it is the omit map that is most useful.

The crystallographic table is still missing some information. For example, there is no indication what the extents of the high-resolution bin are. How many reflections are in the test vs. work set? Are the atoms only protein atoms, or do they include waters, etc.? For the Ramachandran Favored/Allowed, use a slash rather than parentheses in reporting (i.e. 100 / 0).

Reviewer #2

(Remarks to the Author)
The authors have properly addressed all the points I raised. I recommend the paper for publication.

Version 2:

Reviewer comments:

Reviewer #1

(Remarks to the Author)
Unfortunately, problems of (1) methodology and (2) interpretation remain in the CD data.

1. Methodology. Normalizing data against each other is inappropriate when one is trying to determine if there are bona fide differences among protein samples. The normalization should instead be against protein concentration – i.e., each sample normalized by its concentration. The authors report the unnormalized unit mdeg rather than the standard protein concentration-normalized unit (mdeg•cm^2•dmol^-1).

As a result, the authors still have not demonstrated that the CD profiles in Figs. 11B (and S8G) are similar between wild-type and mutant proteins. If they are not, then the point below applies to this/these case/s as well.

2. Interpretation. While it is true that the data shown in Supp. Fig. 7B provide evidence of structured proteins, the spectra are different. The more negative signal at about 215-218 nm suggests greater β-strand content in the mutants compared to wild-type. The use of the term “minor” to describe these differences is qualitative, and if the authors would like to use this term, they should deconvolve the spectra to quantify the percentage of α-helix, β-strand, and random coil, so that a reader can evaluate whether the changes are minor, moderate, or major.

Nevertheless, the authors should indicate that the CD data do not provide validation of the structural results as the spectra

differ. The effect on binding could be due to the loss of a direct contact as observed in the crystal structure, or an alteration in the structure (increased β-sheet) that results in an indirect effect (by changing the conformation of other amino acids that form direct contacts). There is simply no way of knowing without further analysis whether the loss of binding is a direct or indirect effect.

Open Access This Peer Review File is licensed under a Creative Commons Attribution 4.0 International License, which permits use, sharing, adaptation, distribution and reproduction in any medium or format, as long as you give appropriate credit to the original author(s) and the source, provide a link to the Creative Commons license, and indicate if changes were made.
In cases where reviewers are anonymous, credit should be given to 'Anonymous Referee' and the source.
The images or other third party material in this Peer Review File are included in the article's Creative Commons license, unless indicated otherwise in a credit line to the material. If material is not included in the article's Creative Commons license and your intended use is not permitted by statutory regulation or exceeds the permitted use, you will need to obtain permission directly from the copyright holder.
To view a copy of this license, visit https://creativecommons.org/licenses/by/4.0/

We thank the reviewers for their positive words about the paper and are highly appreciative of their suggestions for improvements to the manuscript. Here, we submit a manuscript with revisions that have addressed all the reviewers' comments. We further detail this justification below.

REVIEWER COMMENTS

Reviewer #1 (Remarks to the Author):

Rumpret et al. report that interaction between the surface-localized β protein of S. agalactiae (Group B Streptococcus) and human LILRB3, an inhibitory immune receptor expressed by neutrophils, antagonizes Fc receptor-mediated antibacterial responses. This is an important result based on an extensive and varied set of data. The paper is generally strong although there are some weaknesses.

The authors demonstrate that β protein and LILRB3 interact directly, and identify the particular β protein domains (B6C and B75K) and LILRB3 regions (D3-loop-D4) responsible for the interaction. They further show that the interaction activates signaling downstream of LILRB3 in a T cell reporter system, and decreases the oxidative burst triggered by engagement of FcRIIa in human neutrophils.

The most compelling evidence that this interaction is biologically significant comes from a neutrophil killing assay (Fig. 7C). The authors show that loss of β protein results in a decrease in survival of GBS from slightly less than 100% to about 75%, which is not a major effect but a statistically significant one nevertheless. This loss in GBS survival can be complemented by exogenous expression of β protein from a plasmid, but importantly, not by a β protein mutant (βΔLILRB3) containing five Ala-substitutions that eliminate binding to LILRB3. However, a major issue with this experiment is that the authors do not validate βΔLILRB3 for surface expression and specific loss of function GBS. This sort of validation is indeed carried out but in L. lactis (Supplementary Figure 8). The authors need to carry out these validation experiments in the relevant biological context, i.e., in GBS.

<u>Response:</u> We agree with the reviewer that a more robust validation of the GBS strains would strengthen the work. Therefore, we have quantified β protein expression (Figure A) and interaction with immune receptors (Figure B) for the complemented GBS strains utilised in Fig 7. The data appear in Supplementary Fig. 8, and we have updated the results as follows "Given this, we complemented the *S. agalactiae* Δ*bac* strain with pOri23.*bac* or pOri23.*bac*$^{\Delta LILRB3}$ vectors and confirmed they have comparable levels of surface β protein expression **[Supplementary Fig. 8E]** and differ in LILRB3 interaction **[Supplementary Fig. 8F]**." at lines 376 to 379.

There are similar issues with the rigor of the data in the some of the biochemical experiments when it comes to validating negative results (see below).

Response: We thank the reviewer for providing these comments and have addressed them one by one below.

The authors also show a correlation between β protein expression and GBS lineages that cause invasive disease in older adults. They infer from this that "targeting inhibitory receptors most likely provides an immune evasion mechanism when antibody responses are waning, such as in immunocompromised or elderly individuals." Why would this be? It seems more logical that targeting inhibitory receptors would be need when antibody responses are strong. While the correlation exists, the implications require further consideration.

Response: We thank the reviewer for raising this point, and we agree that further clarification is required in our logic. Antibody responses are on a functional spectrum. We postulate that when an antibody response is strong (such as in immunocompetent adults), it is so efficient at inducing FcR-mediated killing of *S. agalactiae* that immune evasion mechanisms (such as LILRB3-targeting) cannot overcome the response. Indeed, literature indicates that high antibody titres and responses are protective against *S. agalactiae* diseases. In contrast, when an antibody titre and response are absent (such as in certain newborns), then immune evasion mechanisms that suppress FcR-mediated killing of *S. agalactiae* are largely redundant because there is no antibody response to overcome. We therefore postulate that there is a critical window, where antibody titres and responses are sufficiently low, that they can be effectively overcome by LILRB3-mediated inhibition. This could arise through an age-related decline in antibody responses and/or through synergism with other antibody immune evasion mechanisms.

We have added clarification in the results at lines 415 to 419
"In these hosts, antibody titres and responses may fall within a critical window where they can be overcome by *S. agalactiae,* triggering LILRB3-mediated inhibition; conversely, robust antibody titres and responses in immunocompetent individuals may simply be too efficient for LILRB3-targeting to overcome IgG- and Fc-receptor dependent responses of neutrophils."

We have added clarification in the discussion at lines 580 to 586
"We therefore postulate a model where there is a 'window of opportunity' for *S. agalactiae* to overcome the antibody response. In healthy adults, the antibody response will be too efficient to be overcome by LILRB3-mediated evasion, whereas in neonates that lack an antibody response, this immune evasion mechanism is redundant. However, in hosts with low antibody titres or waning antibody responses, LILRB3-targeting is efficient at overcoming the antibody response and promoting *S. agalactiae* survival."

1. The abstract (and other sections) state "β and LILRB3 interactions suppressed the Fc receptor-mediated antibacterial responses such as respiratory burst and phagocytosis …" However, there are no experiments examining phagocytosis per se.

Response: Killing of *S. agalactiae* in neutrophil assays is typically initiated by phagocytosis. However, we agree with the reviewer that our study does not contain phagocytosis experiments *per se*. We have therefore modified the wording "phagocytosis" in the manuscript.

2. The authors determine binding affinities for rLILRB3 for β-rB6C and β-rB75KN in Figure 3. It seems from Figure 6C that the binding affinity of these individual β protein domains is much lower than that of a fragment that contains both these domains (B6C-IgI3-B75KN). Is this true, and if so, why? The authors should comment on this.

Response: The Reviewer correctly pointed out that the apparent affinity of B6C-IgI3-B75KN is greater than that of either B6C or B75KN alone. Whereas the individual affinities of B6C and B75KN for LILRB3 are comparable to one another (please see Supplemental Tables 1-2), incorporating both of these LILRB3 binding domains into a single, bivalent protein has the effect of enhancing the overall affinity by virtue of decreasing the apparent dissociation rate. This is readily visible in the sensorgrams presented in Fig. 6C, as the Reviewer noted. However, it is also important to mention that the sensorgram amplitudes from injections of B6C-IgI3-B75KN cannot be directly compared to those of either B6C or B75KN-C, due to the much larger molecular weight of B6C-IgI3-B75KN (~71 kDa) versus B6C or B75KN-C (~10-15 kDa). This is because the observed signals in SPR are proportional to the molecular weight of the analyte (i.e., injected) molecule.

3. Why are data reported as geometric mean in Figure 2A but % FITC+ in 2B?

Response: We appreciate the reviewer for bringing this to our attention. We have replaced Fig 2A to show % FITC+ cells to ensure consistency across the figures.

4. In Figures 4B and 4C, the y-axes are designated "Binding to" rB6C and rB75KN, respectively. What is specifically being reported? Why are the values so different between 4B (spanning 0–30) and 4C (spanning 20-100)? The same question applies to Figures 5B and 5C, and Supplementary Figures 3C and 3D.

Response: The experiments in Fig 4B and Fig 4C show the binding of rLILRB3-His molecules (full or truncated ectodomains) to magnetic beads coated with biotinylated-rB6C or biotinylated-rB75KN, respectively. These interactions are detected using FITC-conjugated anti-6xHis mAb and flow cytometry analysis. The differences between 4B and 4C likely reflect (1) differences in coupling efficiency of biotinylated-rB6C vs biotinylated-

rB75KN to streptavidin-coated magnetic beads, and/or (2) differences in affinity of the interactions, as evidenced in the paper, and/or (3) accessibility of the His-tag when rLILRB3 is bound to rB6C vs rB75KN in this assay setup. Similar reasoning for experiments in Supplementary Fig 3C and 3D.

The experiments in Fig 5B and 5E show binding of wildtype rLILRB3-Fc to magnetic beads coated with wildtype and mutated B6C molecules (5B) or wildtype and mutated B75KN molecules (5E). These interactions are detected using HRP-conjugated anti-human-IgG mAb and ELISA. The differences between 4B and 4C likely reflect (1) differences in coupling efficiency of biotinylated-rB6C vs biotinylated-rB75KN to streptavidin-coated magnetic beads, and/or (2) differences in affinity of the interactions, as evidenced in the paper.

The experiments in Fig 5C and 5F show binding of wild-type or mutant rLILRB3-His to magnetic beads coated with wild-type B6C (5C) or wild-type B75KN molecules (5F). These interactions are detected using FITC-conjugated anti-6xHis mAb and flow cytometry analysis. The differences between 5C and 5F likely reflect (1) differences in coupling efficiency of biotinylated-rB6C vs biotinylated-rB75KN to streptavidin-coated magnetic beads, and/or (2) differences in affinity of the interactions, as evidenced in the paper.

The differences in scale across assays above will be reflected by the format of the experiment (flow cytometry vs. ELISA), the detection reagent (different mAbs), affinities of interactions and the efficiency in coating proteins on plates or beads. We note that we do not compare results across different graphs because of this variation.

5. LILRB3 D2-D3-D4 appears to be a weaker binder than D1-D2-D3-D4 and D3-D4 (in Figure 4). Do the authors have an explanation for this?

Response: We found this result surprising. We postulate that D2, when present without D1, sterically hinders the D3-D4 from binding B6C or B75KN.

6. A 2Fo-Fc map is phase biased and thus not meaningful as an indicator of correctness (Supplementary Figure 4A). The authors should instead show an 2Fo-Fc omit map or composite omit map of a representative portion of the electron density (and indicate which regions are being omitted and what sigma level is being depicted).

Response: While we appreciate the Reviewer’s perspective, we disagree with the sentiment that the 2Fo-Fc map is not a useful indicator of structural correctness. However, to address the Reviewer’s point directly, we calculated a 2Fo-Fc composite omit map for chain A of the B6C polypeptide and now present that, along with our initial representation of the 2Fo-Fc map. In the interest of completeness, we have also generated images containing both the 2Fo-Fc map and 2Fo-Fc composite omit maps for Chain B as well. These changes are now presented as an updated version of the Supplementary Figure 4.

7. L. 259, “We then generated ab initio density envelopes, which matched well with the computational models obtained for each complex [Supplementary Fig. 5G-H].” This reviewer disagrees with this qualitative judgment. The envelopes have a great deal of empty space, not occupied by the model. These envelopes seem to be quite poor matches.

Response: We understand the Reviewer’s concern with our original statement. Since SAXS is an inherently low-resolution technique, the envelopes obtained through analysis of SAXS data cannot be expected to show the level of detail and model-to-map correlation typically associated with crystal structures. Still, the average shapes of the molecular models should be consistent with the reconstructed envelopes. We think this statement accurately describes the data we present in our manuscript. So, to rectify the Reviewer’s concern, we have replaced the phrasing “which matched well with the computational model” to “which were consistent with the overall shapes of the computational models obtained for each complex” at lines 258 to 259.

8. The authors identify several amino acids in β protein and LILRB3 as interactors based on structural models. They should specify for each amino acid which partner amino acids are predicted to be contacted.

Response: This is an excellent suggestion. We have included a schematic map of β protein and LILRB3 residues that are predicted to contact each other, in Supplementary Fig 9. The results are updated at lines 311 to 319.

9. Based on negative results from binding experiments in Figure 5, the authors infer that a set of amino acids in β protein contact LILRB3 and vice versa. This is unjustified. The authors do not establish that the negative results are not due to nonspecific loss in structure or stability, as compared to a specific effect on binding. This could be assessed, for example, through comparison of circular dichroism spectra to establish structural integrity and denaturation profiles (increasing temperature or denaturant concentration, as monitored by circular dichroism) for stability. The same issue applies to the multiple mutation βΔLILRB3 construct (in Figure 6).

Response: We have tested whether the alanine mutations that reduce interactions in ELISA and SPR lead to drastic misfolding of proteins by CD analysis, as suggested by the Reviewer. This data is presented in Supplementary Figures 6G, 7B, 8G and 11B. This data shows that the mutated proteins display a similar CD spectrum to wild-type proteins. As there are no drastic changes, this confirms that the mutated proteins are not unfolded or misfolded. The minor variations in the CD spectrum will reflect minor differences in protein concentrations. Therefore, these data, along with other control data described below, confirm the role of these residues at the binding interfaces.

10. A general problem exists in ELISAs that have different components affixed to wells. For example, the data in Figures 5B and 5E can be explained by N287A, Q289A, etc. not adhering to wells as well as the wild-type protein. The authors need to control for this.

Response: To control for the different adherence of wild-type and mutated proteins to wells, we have performed an ELISA in which we measure the adherence of proteins to the plate using HRP-conjugated anti-6xHis antibody. This data shows that each mutated protein binds equally well to the 96-well ELISA plate. We have additionally provided SDS-PAGE analysis for all protein preparations. This is presented in Supplementary Fig. 6B, 6C and 6E (for rB6C), Supplementary Fig. 7A (for LILRB3), Supplementary Fig. 8B, 8C and 8E (for rB75KN) and Supplementary Fig. 11 (for rβ). Text in the results is updated at lines 268 to 273 and lines 297 to 300.

11. The data purported to show that a single β protein molecule can bind two molecules of LILRB3 (and thus cause clustering for signaling) are not convincing. The result that a 2:1 stoichiometry inhibits better than 1:1 may simply reflect greater fractional occupancy at higher stoichiometry. The SAXS data shown in Supplementary Table 3 are more convincing. However, an even more convincing experiment for the authors' point about signaling would be the following. If they were able to show that mutations in B6C knock out binding specifically and others in B75KN do likewise (i.e., no effect on structure or stability), then intact β protein containing only the B6C mutation or only the B75KN mutation should still bind LILRB3 (1:1 stoichiometry) but no longer be able to signal (lacking 2:1 stoichiometry).

Response: We agree with the Reviewer that it would be optimal to test this idea through the use of mutagenesis experiments. However, we would ask the Reviewer and the Editor to consider the compilation of the data we provide, addressing the stoichiometry of this interaction.

1. As the Reviewer notes, comparative SAXS analysis between the B6C-IgI3-B75KN fragment alone as well as with two equivalents of LILRB3 shows apparent molecular weight values well-matched to their predicted values if both LILRB3-binding sites within the B6C-IgI3-B75KN protein were occupied. This can be interpreted as confirmation of the stoichiometry and model we propose, since the LILRB3 protein on its own behaves as a monomer (Supplementary Table 3).
2. As the Reviewer also points out, the loss of B6C-IgI3-B75KN's ability to bind surface-captured LILRB3 in the presence of increasing levels of soluble LILRB3 can be interpreted as increased occupancy of the individual LILRB3-binding sites present in this β protein fragment. Indeed, that is why we explored this experimental setup during this study. While our SPR data do not show complete loss of binding for the 1:2 β:LILRB3 sample, we think its important to remember that all complexes except those of the highest affinities (e.g. sub-nanomolar K_D values) will dissociate in SPR experiments whether they're preformed or not: as the K_D values for the LILRB3-binding to the two sites identified within β lie the low 10^{-7} M range, some dissociation will necessarily occur during this experiment.

Thus, the residual binding observed in the 1:2 can be explained by a population of the binding sites becoming available to bind the surface-captured LILRB3 molecules. Ultimately, we hoped that crystallographic analysis of this B6C-IgI3-B75KN/$(LILRB3)_2$ complex would have provided incontrovertible proof of the nature of this bridging interaction. But in the absence of that, we think that the combination of the SAXS data and SPR experiment, along with the mutagenesis data provided elsewhere in our manuscript, makes it far more likely than not that the proposed β:LILRB3 stoichiometry is indeed 1:2.

12. SDS-PAGE is shown to establish the purity of LILRB3 constructs in Supp. Fig. 3A, but equivalent data are not provided for β protein variants. They should be.

Response: We have now included an SDS-PAGE image of all the rB75KN, rB6C and rLILRB3, and rβ mutants generated in this study. This data is presented in Supplementary Fig 6, 7, 8 and 11.

Minor Comments

1. In Figure 2G and other figures with Amplex Red data, it is difficult to discern individual curves, as the data points are large and obscure each other.

Response: We have updated Fig 2G, 3F, 5G, 5I, and Supp Fig 2G with line graphs so that each condition is more clearly visible.

2. The designation "B6C-IgI3-B75KN" appears without an introduction. Readers who are not intimately familiar with β protein domains will be confused by this, including this reviewer. This term should be explained.

Response: We have added "(a protein containing the B6C domain and B75KN domain and the IgI3 domain sandwiched between as in the native protein, see **Supplementary Fig. 2A**)" to lines 332 to 334 for improved clarity.

3. In descriptions of ELISAs in the main text or figure legends, it would be useful to designate which component is affixed and how the soluble component is detected.

Response: We have added "coated-" to the legends of Fig 1G, 5C, 5F to designate which component is affixed. We have added how each soluble component was detected in legends in Fig 1, 3, 4, 5, Supp Fig 3, 6.

4. In figures showing protein structures, it would be useful to label the N- and C-termini (by that designation or by numbers).

Response: We have updates Fig 3, 5, Supp Fig 4, 5, 6, 8 with annotation of N and C terminus.

5. There are various typos throughout. For example, L. 130 “signifcantly". Careful inspection of the Word spell checker is warranted.

Response: We apologise for this error and have updated. We have checked the spelling throughout the document.

6. L. 178, “Similar inhibitory effects were identified when we stimulated FcαRI (CD89) on neutrophils [Supplementary Fig. 2A and 2B].” The authors presumably mean Supplementary Figures 1D and 1E.

Response: We apologise for this error and have updated.

7. In Supplementary Figure 4B, the authors should be explicit regarding which color corresponds to which model.

Response: We have updated the Figure legend.

8. In Figures 5C and 5F, it is not clear which proteins are affixed to ELISA wells.

Response: We have added “coated-” to the Figure legends to provide clarity.

9. The source of human serum is not indicated.

Response: We have added this information into materials and methods, “Pooled human serum (from a minimum of 4 donors) was prepared from blood collected from healthy human donors using BD Vacutainer Tube Sst Advance Gold Blood Collection Tubes, following manufacturers' protocols.” at lines 620 to 622.

10. L. 332, “Indeed, both coated and soluble rβ, but not coated and soluble rβΔLILRB3, stimulated GFP expression in LILRB3-CD3ζ reporter cells but not control cells [Fig. 6D].” This reviewer assumes coated means on dynabeads. Is that correct? It should be explicitly stated.

Response: In these experiments, we compared GFP-expression of LILRB3-CD3ζ reporter cells or controls after stimulation with soluble rβ, soluble rβΔLILRB3, or surface coated-rβ or surface-coated rβΔLILRB3. For surface-coated, this is on the surface of cell-culture plates, not on dynabeads. We have corrected the text by explicitly stating “surface-coated (on the microtiter well)” at line 352.

11. In Figure 7C, it appears that wild-type A909 is not killed by human neutrophils within the timeframe of the experiment (about 100% survival). Is this expected? The Materials and Methods states: “At designated time points after incubation at 37°C, samples were lysed in 0.3% saponin, serially diluted into sterile PBS and plated onto TH agar plates. After culturing at 37°C for 1 hour, the % survival was calculated as CFU after incubation

with neutrophils relative to CFU of the inoculum." What were these designated time points? Is this a 1 hour experiment? Is that what "culturing" refers to?

Response: We have now corrected the method to read "One hour after incubation at 37°C, samples were lysed in 0.3% saponin, serially diluted into sterile PBS and plated onto TH agar plates. After culturing at 37°C for 16 hours, the % survival was calculated as CFU after incubation with neutrophils relative to CFU of the inoculum at time zero." at lines 924.

12. The Materials and Methods section appears to be lacking a description of the ELISA experiment in Supplementary Figure 9C in its use of whole bacteria.

Response: We have added the word "purified" to clarify that it is a protein and not whole bacteria in the ELISA-assays in the Supp Figure, as requested. We have also updated the method paragraph at lines 769 to 771.

13. Figure 9 has details in the legend (e.g., Syk, SHP-1, etc.) that are not depicted in the figure.

Response: We agree this is an important point. Therefore, we have updated the Fig 9 to show the signalling processes in further depth. Additionally, we have been more cautious in the Figure legend since this study has not measured Syk, SHP-1, etc. to read "This likely leads to, though requires experimental validation, the recruitment of protein tyrosine-phosphatases (such as SHP-1, SHP-2 and/or SHIP) that can counteract phosphorylation of Syk and downstream signalling molecules. " lines 1394 to 1397.

14. L. 503, "Since other S. aureus …" What does "other" mean in this context?
Response: We thank the reviewer for noticing this error. We note that a previous study identified that *S. aureus* and *E. coli* displayed enhanced adhesion to cells overexpressing LILRB3[37], though the bacterial ligands of LILRB3 and the functional consequences of these interactions remain to be defined. " Lines 535 to 538.

15. In Table 1, the extents of the highest resolution shell should be stated. Statistics for the highest resolution shell are missing in the refinement section. How many reflections were in the work and free sets?

Response: We apologise for this omission in the original version of Table 1. To correct this, we have updated our table to include information pertaining to the highest resolution shell for both the experimental data and crystallographic refinement, the total number of reflections used in refinement, as well as information about to the corresponding test set.

16. L. 745, "Friedel pairs" should be "Bijvoet pairs."
Response: We have corrected as requested.

17. The authors should double check their references and bibliography. For example, L. 159 references (Zhao et al., 2020b), which shows up twice in the numbered bibliography (ref. 18 and 83).

Response: We have double-checked the references and have removed all duplications. The following references were merged:

22 and 37 (Alvarez, 2008)
16 and 25 (Daeron, 2008)
18 and 83 (Zhao, 2020)

18. The 2B4 T cell reporter system appears to be murine rather than human in origin, although the chain of references to ferret this out is long. In any case, it is worthwhile clarifying this.

Response: We have added "Murine" in the M&M at line 876 and the results at line 158.

Reviewer #2 (Remarks to the Author):

This is an interesting and important paper elucidating the function of inhibitory LILRB3 on bacterial infection. Because function of LILRB3 on bacterial infection was not known, their findings that LILRB3 binds to β protein of S. agalactiae is quite important. They validated the interaction between LILRB3 and β protein not only by using recombinant protein but also by structual analysis of LILRB3. Furthermore, the predicted binding sites were confirmed by point mutations. In addition, they have demonstrated that LILRB3 signaling downregulates the killing activity of neutrophils against β protein-expressing bacteria. Their studies are well organaized and the results are clear. The followings are point that may improve the paper.

Response: We thank the reviewer for providing the comments, and we have addressed them one by one below.

1. The authors demonstrated that some of clinical isolate of S. agalactiae express β protein and bind to LILRB3. These findings are important to understand the pathophysiological function of LILRB3 and β protein interaction. Do these isolates expressing β protein also stimulate LILRB3-reporter cells?

Response: We agree this is an important consideration. Therefore, we have tested whether 3 different clinical isolates that express β protein also stimulate LILRB3-CD3ζ reporter cells. This data is now included in Supplementary Fig. 13. We have included a description of this in the results "We confirmed that β protein expression in clinical isolates is a

functional ligand of LILRB3 using the LILRB3-CD3ζreporter cells **[Supplementary Fig. 10]**." at lines 443 to 445.

2. In the discussion, the authors described that binding of LILRB3 to E. coli and S. aureus has been already publised in reference 39. It looks inconsistent to the authors' discription that this is the first report showing the interaction of ILRB3 and bacteria. However, reference 39 is the studies of mouse PIR-B not human LILRB3. Although PIR-B has homology to human LILRB3, the amino acid homology is not so high. Therefore, they shoud describe precisely the differene between PIR-B and LILRB3 as well as difference from this paper.

Response: We thank the reviewer for identifying that this point requires further clarity.

We note that in our submission, we did not claim that this study is the first report showing the interaction of LILRB3 and bacteria. Instead, we claimed in the Discussion that "By presenting experimental evidence that *S. agalactiae* targets the inhibitory LILRB3 receptor to suppress antibody- and FcR-dependent responses of human neutrophils, we highlight for the first time that bacterial pathogens target inhibitory receptors to evade phagocyte-mediated adaptive antibacterial immunity."

However, we agree with the reviewer that we can improve the communication of the difference in the study of Nakayama et al. in Reference 39 and our study.

Nakayama *et al.* showed 1) that *S. aureus* interacts with mouse PIR-B and showed that these interactions modified the cytokine production in murine bone marrow-derived macrophages, and 2) that *S. aureus* and *E. coli* interact with cells overexpressing LILRB3 (ILT5) and LILRB1 (ILT2) in Figure 9. However, this paper does not robustly define these interactions beyond binding of bacteria to cell lines, nor does it investigate the mechanisms that *S. aureus* or *E. coli* utilise to interact with LILRB3 or LILRB1, nor does it investigate any functional consequences of these interactions. In contrast, our study is the first to 1) robustly identify a pathogenic bacterium that interacts with LILRB3, 2) determine the bacterial ligand responsible for this interaction, and 3) investigate the functional consequences of these interactions.

We have clarified the differences in our findings "We note that a previous study identified that *S. aureus* and *E. coli* displayed enhanced adhesion to cells over-expressing LILRB3[39], though the bacterial ligands of LILRB3 and the functional consequences of these interactions remain to be defined. Nonetheless, our first definition of the first bacterial ligand of LILRB3 and function studies, combined with reports of other bacteria-LILRB3 interactions, suggests that multiple bacterial species have evolved distinct ligands to target LILRB3 on neutrophils." at lines 535 to 541

We have clarified that the PIR-B ectodomain contains 6 Ig-like domains, whilst the LILRB3 ectodomain contains 4 Ig-like domains in the results "Of note, human LILRB3 and murine

PIR-B do not share a high degree of structural conservation **[Supplementary Fig. 9A]**, represented by the fact that the ectodomain of murine PIR-B possesses 6 Ig-like domains compared to 4 Ig-like domains of LILRB3." at lines 451 to 454

3. In the last part, the authors conclude that inhibitory receptors are also involved in dampening innate PRR-dependent immune responses to bacteria. However, the authors did not analyzed PRR-dependent reponse of neutrophils and it is unclear whether inhibitory receptors are involved in PRR-dependent immune response. These discription shold be revised.

Response: We thank the reviewer for this comment. We agree that additional clarity is required and that our study focuses specifically on antibody-dependent responses and does not include direct measurements of PRR-dependent signalling. We intended to place our findings in the context of the field where it is established that bacterial interactions with inhibitory receptors dampen PRR-dependent sensing and cellular responses, as evidenced for *S. aureus* and the mouse PIR-B receptor [PMID: 17371981] and Gram-negative bacterial pathogens and the human CEACAM1 receptor [PMID:18836450].

As suggested, we have revised and improved clarity in the sentence the reviewer is referring to: "Inhibitory receptor targeting by bacteria has been shown to dampen innate PRR-dependent immune responses, whilst here we show these interactions can dampen adaptive antibody-dependent immune responses." at lines 602 to 604.

4. Although the authors suggest that LILRB3 is important to immune response to variety of bacteria. If so, various types of bacteria also should use LILRB3 for immune evasion. Why only S. agalactiae use LILRB3 for immune evasion?

Response: As we have discussed in lines 535 to 541, other bacterial pathogens (*S. aureus* and *E. coli*) have been demonstrated to have enhanced adhesion to cells over-expressing LILRB3. While the ligands for LILRB3 for these bacteria have not been identified, and functional studies of the consequences of these interactions have not been performed, we speculate that these human bacterial pathogens (and potentially others) utilise LILRB3 for immune evasion similarly to *S. agalactiae*.

5. For the analysis of the interaction between LILRB3 and β protein on bacteria, some experiments were done by flow cytometry but other were done with ELISA. What is the difference?

Response: We thank the reviewer for this observation. Both assays measure the binding of rLILRB3 to bacteria. The biggest differences in these assays are the detection method of fluorescence (flow cytometry) versus chemiluminescence (ELISA). Additionally, flow cytometry allows us to observe binding at the individual bacterium level within a population, while the chemiluminescent ELISA provides a bulk-population measurement.

We note that both approaches in our laboratory independently demonstrate the same conclusion: that rLILRB3 binds specifically to the *S. agalactiae* surface via β protein.

6. There are some LILRB3CD3ζ. It should be LILRB3-CD3ζ.

Response: We agree. We have modified all to LILRB3-CD3ζ.

Reviewer #3 (Remarks to the Author):

This paper by Rumpret et al., reveals a previously unrecognized immune evasion strategy where the Streptococcus agalatiae (GBS) surface β protein directly targets a the neutrophil inhibitory receptor LILRB3 to suppress antibody-mediated immunity.

The study is a tour de force in molecular microbiology, neutrophil functional assays, protein chemistry and structural biology.

Antibody-mediated immunity is crucial for defending against bacterial pathogens, primarily through Fc receptor (FcR)-mediated phagocytosis and bacterial killing by neutrophils. Inhibitory immune receptors, such as LILRB3, regulate immune cell activation, but it has been unclear whether bacteria directly target these receptors to evade antibody-driven responses.

By using both loss of function and gain of function experiments, based on bac knockouts lacking β protein expression in GBS, or heterologous expression of β protein in Lactococcus lactis, it is very clear that β protein is required and sufficient to interact with purified LILRB3. Furthermore, it is shown GBS expressing β protein binds to cells expressing LILRB3 and can crosslink the receptor leading to inhibitory signals and reduced Fc-receptor mediated oxidative burst.

Further mapping of the interaction indicate that two distinct domains of β are responsible for high affinity binding to. LILRB3. Furthermore, these two domains are sufficient to block FcR-mediated ROS production in neutrophils. Additional mapping on the host side revealed that Ig domains D3 to D4 in LILRB3 are bound by the two β domains.

Further structural analysis eluciated which amino acids in the two β domains that are involved in binding of LILRB3 but not of some of the other know ligands for β.

Analysis of clinical GBS isolates revealed that β expressing strains are associated with lineages that cause disease in non-pregnant adults. Furthermore, β can alone crosslink LILRB3 leading to inhibitory signals, and a bac knockout GBS transcomplemented with bac encoding β only interacting with LILRB3 still impairs killing by neutrophils indicating that interaction with LILRB3 is a major contributor to this type of immune evasion.

Broader implications of the study is that bacterial pathogens can directly target human inhibitory receptors to subvert antibody-dependent phagocyte responses. Understanding these interactions is critical for designing effective vaccines and antibody-based therapies, especially for vulnerable populations.

The study is extremely well conceived and distinctly presented both in text and figures. I'm hard pressed to find any major flaws, but the anti-phosphorylation Western blots are of lower quality than the rest of the data. Not a major problem, since the trends seen in these fits the other functional data, but it would be nice to see more convincing Westerns or other phosphorylation assays.

Response: We sincerely thank the reviewer for their highly encouraging and supportive assessment of our manuscript. Regarding the anti-phosphorylation Western blots, we appreciate the reviewer's attention to detail. We note that phosphorylation events are rapid and transient in nature, and that detection is challenged by the high abundance of intracellular proteases.

As the reviewer noted that the current blots are "not a major problem" because they align with our robust functional data, we have

- provided the original uncropped blots in the Source Data, providing further confidence
- modified our sentences in the Results section "Collectively, this indicates that the pathogen-derived β protein targets LILRB3 by two different domains (B6C and B75KN) and induces LILRB3 phosphorylation. This biochemical signalling translates into a functional phenotype as IgG/FcγR-dependent and IgA/FcαRI-dependent cellular immune responses are suppressed." at lines 213 to 315 to further emphasise that the biochemical signalling translates directly into the phenotype
- added a comment in the Discussion to acknowledge that while the current data are consistent, future studies utilising a temporal analysis of LILRB3 phosphorylation and signalling events in primary cells will be valuable "The phosphorylation of ITIMs and the recruitment of SHP-1 or SHP-2 to ITIMs in inhibitory receptors are often rapid and transient in nature. Whilst our current is consistent with β protein inducing LILRB3 ITIM phosphorylation and subsequent signalling events to inhibit the FcγR-dependent activation and effector responses of neutrophils, future studies utilising a temporal analysis of LILRB3 phosphorylation and signalling events in primary neutrophils will be valuable to fully elucidate the kinetic window of inhibition induced by bacterial pathogens." at lines 487 to 493. We believe that these clarifications, combined with the strong correlation between the signalling and functional data, sufficiently support the conclusions of the manuscript.

We thank the reviewers for their review and appreciate their suggestions for improvements. Here, we submit a revised manuscript that addresses all reviewers' comments. We further detail this justification below.

REVIEWER COMMENTS

The revision by Rumpret et al. has suitably addressed almost all of my prior concerns. Most importantly, the authors have carried out controls for expression and function of wild-type and ΔLILRB3 β protein as expressed by S. agalactiae (Figs. S12E and F). The figures show comparable expression, but curiously the authors did not carry out statistical comparison between the two (i.e., pOri23.bac vs. pOri23bac(ΔLILRB3); they should. They also find that ΔLILRB3 β protein maintains CECAM- and Siglec5-binding, suggesting that functional effects on LILR3 binding are not due to nonspecific structural alteration or loss of stability.

Response: We thank the reviewer for their comments. Fig S12E has been updated to now include the statistical analysis requested. This shows that there is a significant difference in the binding of anti-β IgG to GBS carrying pOri23.*bac* vs. pOri23.$bac^{\Delta LILRB3}$. We note that despite the finding that pOri23.$bac^{\Delta LILRB3}$ strain has a higher β protein expression (or higher accessibility of anti-β IgG), it has reduced blood survival compared to pOri23.*bac* strain. We have commented on this in the manuscript at lines 386-389.

"The Δ*bac* strain carrying pOri23.$bac^{\Delta LILRB3}$ had a higher expression level of surface β protein **[Supplementary Fig. 12E]**. However, there was no significant difference in their interaction with CEACAM1 or Siglec5 **[Supplementary Fig. 12F]**. In contrast, they differed in LILRB3 interaction."

The major point that the authors seem to have ignored in the paper and rebuttal is evaluation of the stability of mutant proteins. The authors present circular dichroism (CD) data at a single temperature, but the suggestion to carry out a temperature or denaturant melt, as monitored by CD, to assay stability was not carried out or commented on. A Materials and Methods section for the CD experiments is lacking. There is no indication of what temperature these experiments were done and how many independent times they were done. The overall concern regarding stability would be abated if in vitro binding experiments using the mutant proteins were carried out at the same temperature as the CD analysis. Nevertheless, the CD spectra of wild-type and mutant LILRB3 are clearly different (Fig. 7B), most tellingly having different zero-crossings. This indicates that the secondary structure differs between wild-type and mutants, and the secondary structure also appears to differ among mutants. Therefore, the statement that wild-type and mutant LILRB3 "displayed similar folding structure" (L. 281) is unjustified. Similarly, rβ and rβ(ΔLILRB3) appear to differ in structure (Fig. S11B) and perhaps wild-type and L721A (Fig. S8G), but it is difficult to gauge given the lack of details and statistics. In the latter two cases, similar zero-crossings occur for wild-type

and mutant, and so differences in spectra may be due to errors in protein concentration determination rather than bona fide structural alteration.

Response:

We sincerely apologise for the oversight in omitting the CD Materials and Methods from our previous revision. We have now fully updated the manuscript to include these details at Lines 878 to 887.

“**Circular Dichroism**

Far-UV circular dichroism spectra were recorded at room temperature on either a Jasco J-815 CD spectrophotometer (B6C and B75KN-C variants) or a Chirascan V100 instrument (rLILRB3-His and full-length β variants) 99,100. Samples were prepared in either ddH2O alone (B6C or B75KN-C variants), 10 mM sodium phosphate, 50 mM sodium fluoride, pH 7.4 (LILRB3-his variants) or standard PBS (β and $β^{ΔLILRB3}$). Measurements were performed in a 1 mm pathlength quartz cuvette over a wavelength range of 190 to 260 nm. Five experimental scans were averaged for each protein, buffer baseline was subtracted, and a Savitzky–Golay smoothing algorithm was applied. Where indicated, the spectra were scaled to the ellipticity at 222 nm.”

Regarding interpretation of CD spectra:-

Consistency Between CD and functional assays: Per guidance provided in the previous round of peer review, the Reviewer stated that concerns regarding stability are abated if binding experiments and CD analysis are performed at the same temperature. We can confirm that all *in vitro* binding assays and the CD spectra were conducted at room temperature, 22 °C to 25 °C. Therefore, our CD spectra directly represent the structural state of the proteins during the interaction studies. We have updated the text at lines 358 to 360

“The wildtype and mutated proteins had almost superimposable circular dichroism spectra, indicating $rβ^{ΔLILRB3}$ is folding into a native-like rβ conformation **[Supplementary Fig. 11B]**.”

CD spectral differences due to minor changes in protein concentration: The reviewer correctly notes that differences in CD spectra may be due to errors in protein concentration determination rather than bona fide structural alterations. To assess this, we have normalised the CD spectrum questioned by the reviewer by scaling the data to the mean residue ellipticity at 222 nm. By anchoring the spectra at this characteristic wavelength, we can better compare the overall spectral profile and zero-crossing points. As shown in the updated Supplementary Figure 11B, the scaled curves for rβ and $rβ^{ΔLILRB3}$ are nearly superimposable. Therefore, we conclude that our collective data suggest that the β-protein mutant is folding into a native-like conformation at the temperatures used for functional studies.

CD spectral differences due to minor structural variations: We have updated **Supp. Fig 7C,** which shows the average of five measurements for the LILRB3 protein CD spectra. Whilst scaling the data to the mean residue ellipticity at 222 nm indicated comparable CD spectra for β and $\beta^{\Delta LILRB3}$, this did not indicate superimposable spectra for LILRB3 point mutants. However, from these spectra we note 1) the lack of the single spectra minima at 200 nM indicates that the proteins are not completely unfolded, 2) spectral minima at ~215 nm are indicative of β-sheet secondary structures, 3) the single spectral maxima at ~230 nm are indicative of certain β-sheet containing proteins. This suggests differences in CD spectra reflect bona fide local structural variations. In support, 1) the mutations in LILRB3 are located within loops between β-sheets or within disordered linker regions between the Ig domains **[Supp. Fig 7B]**. Mutations in flexible regions (as opposed to β-sheets of the Ig domains) would not be expected to compromise global folding of Ig domains. 2) all rLILRB3 were successfully expressed and secreted from EXPI293F cells, indicating they were not unfolded or otherwise aggregated following synthesis and post-translational processing. 3) Importantly, not all LILRB3 mutants show loss-of-function despite differences in CD spectra of wildtype and mutant LILRB3. For example, $LILRB3^{H375A}$ has no significant change in rB6C binding **[Fig. 5C]**.

Collectively, we conclude that although the observed CD spectral shifts most likely represent minor local perturbations in the loop regions, they are not consistent with a global loss of tertiary structure that would compromise the interpretation of the functional studies involving these proteins. Consequently, we have updated the text at lines 281 to 290

"The mutation of single residues in the loop regions of the LILRB3 Ig-domains **[Supplementary Fig. 7B]** to alanine did not lead to global unfolding of the LILRB3 protein as assessed using circular dichroism **[Supplementary Fig. 7C]**; even though minor variations in the circular dichroism spectra were noted, these likely arose from local structural variations within the mutated loop regions of LILRB3 molecules. Mutation of LILRB3 at residues Y322, W348, W349 or K376 significantly reduced the interaction with B6C **[Fig. 5C]**, whilst mutation of residues D323 or T324 reduced binding to rB6C, consistent with our structural information obtained earlier. Of note, the mutation at the residue H375 in LILRB3, which had a minor variation in the circular dichroism spectra compared to LILRB3, did not have any impact on interaction with B6C **[Fig. 5C]**."

A smaller point is that Figure S4 appears to show density only around atoms. This reviewer does not suspect that the structure is incorrect, but for rigor, entire density maps should be shown, and for clarity, around some 'representative' region of the map and protein rather than the entire protein. It is impossible to evaluate the quality of the map in the current figures. The designation "1.2s" should be replaced by "1.2σ." The issue of phase bias is well encapsulated by Kathryn Cowtan's famous duck and cat examples of Fourier Transforms (http://www.ysbl.york.ac.uk/~cowtan/fourier/magic.html). This point is also in Berhard Rupp's Biomolecular Crystallography textbook, in which entirely random amplitudes but

with ‘correct’ phases are shown to yield an interpretable map, but not vice versa. This reviewer has no objection to showing both 2Fo-Fc and omit 2Fo-Fc maps, but it is the omit map that is most useful.

Response: The Reviewer’s point is taken. Therefore, we have modified Figure S4 according to these suggestions. The new layout includes the same images in Panels A and B as before, but now also includes (i) two orthogonal representations of the full extent of the composite omit map as new Panels C and D, and (ii) representative regions of the model/omit map correlation for chains A and B, respectively, as panels E and F. Images G and H, which show comparison of experimentally-determined structures to AlphaFold models, remain from the previous version. Lastly, we thank the reviewer for their comment, noting the missing Greek letter in our legend. We have corrected this typographical error to show 1.2σ, as requested.

The crystallographic table is still missing some information. For example, there is no indication what the extents of the high-resolution bin are. How many reflections are in the test vs. work set? Are the atoms only protein atoms, or do they include waters, etc.? For the Ramachandran Favored/Allowed, use a slash rather than parentheses in reporting (i.e. 100 / 0).

Response: We have updated the table to include information on the i) extents of the highest-resolution bin, ii) the number of reflections in the test set, expressed both numerically and as a percentage of the total number of reflections, and iii) the total number of atoms and the number of ordered solvent molecules in the PDB file. We have also corrected the formatting of the information for the Ramachandran plot as requested.

We thank the reviewers for the rigorous standard they have held our manuscript to, and we agree that incorporating these biophysical reporting standards and interpretative caveats has ultimately strengthened the paper. We have addressed both remaining points below and updated the manuscript and figures accordingly. Here, we submit a revised manuscript that addresses all comments.

Reviewer #1 (Remarks to the Author):

Unfortunately, problems of (1) methodology and (2) interpretation remain in the CD data.

1. Methodology. Normalizing data against each other is inappropriate when one is trying to determine if there are bona fide differences among protein samples. The normalization should instead be against protein concentration – i.e., each sample normalized by its concentration. The authors report the unnormalized unit mdeg rather than the standard protein concentration-normalized unit (mdeg•cm^2•dmol^-1).

As a result, the authors still have not demonstrated that the CD profiles in Figs. 11B (and S8G) are similar between wild-type and mutant proteins. If they are not, then the point below applies to this/these case/s as well.

2. Interpretation. While it is true that the data shown in Supp. Fig. 7B provide evidence of structured proteins, the spectra are different. The more negative signal at about 215-218 nm suggests greater β-strand content in the mutants compared to wild-type. The use of the term “minor” to describe these differences is qualitative, and if the authors would like to use this term, they should deconvolve the spectra to quantify the percentage of α-helix, β-strand, and random coil, so that a reader can evaluate whether the changes are minor, moderate, or major.

Nevertheless, the authors should indicate that the CD data do not provide validation of the structural results as the spectra differ. The effect on binding could be due to the loss of a direct contact as observed in the crystal structure, or an alteration in the structure (increased β-sheet) that results in an indirect effect (by changing the conformation of other amino acids that form direct contacts). There is simply no way of knowing without further analysis whether the loss of binding is a direct or indirect effect.

Response: We thank the reviewer for their rigorous assessment. In addition to presenting the standard protein concentration-normalised unit (mdeg•cm^2•dmol^-1), a standard approach is to analyse and present CD [mdeg]. Please see papers such as 1) Conners et al. “The Moraxella adhesin UspA1 binds to its human CEACAM1 receptor by a deformable trimeric coiled-coil” The EMBO Journal. 2008. 27(12):1779–1789, 2) Glossop et al. “Retro-inversion imparts antimycobacterial specificity to host defense peptides” Nature Communications 2025 Dec 7;17(1):469, 3) Wang et al. 2024 “CD5L associates with IgM via the J chain” Nature Communications 15(1):8397.

Our rationale for scaling the CD to 222 nm was to account for potential minor discrepancies in protein concentration determination. This is a recognised approach to determine if differences in raw data are due to global unfolding or concentration differences. Please See: Miles et al. "Tools and methods for circular dichroism spectroscopy of proteins: a tutorial review" Chem Soc Rev. 2021 Aug 7;50(15):8400-8413 (with 380 citations).

We agree with the reviewer that the deeper negative signal around 215–218 nm in Supp. Fig. 7C. indicates an altered secondary structure. Additionally, we agree that our previous use of the term "minor" to describe these differences was inappropriate, and we have removed this qualitative description. Because the secondary structures clearly differ, these CD data do not definitively validate the structural results. We additionally reiterate that not all LILRB3 mutants show loss-of-function despite differences in CD spectra of wild-type and mutant LILRB3. For example, LILRB3^{H375A} has no significant change in rB6C binding, but has a CD spectrum that is equivalent to LILRB3 mutants that do have a loss of rB6C binding. Taken together, we agree that there is no way to definitively rule out that the loss of binding observed in mutants is an indirect effect of this structural alteration (i.e., increased β-sheet content altering the conformation of other amino acids that form direct contacts) rather than solely the loss of a direct contact point.

We have removed the qualitative word minor in lines 283 to 289 to now read

"The mutation of single residues in the loop regions of the LILRB3 Ig-domains **[Supplementary Fig. 7B]** to alanine did not lead to global unfolding of the LILRB3 protein as assessed using circular dichroism **[Supplementary Fig. 7C]**. However, the spectra of mutant LILRB3 had a more negative signal observed at ~215-218 nm, suggesting there could be an increase in β-strand content in the mutants. Mutation of LILRB3 at residues Y322, W348, W349 or K376 significantly reduced the interaction with B6C **[Fig. 5C]**, whilst mutation of residues D323 or T324 reduced binding to rB6C. Of note, the mutation at the residue H375 in LILRB3, which had variation in the circular dichroism spectra compared to LILRB3, did not have any impact on interaction with B6C **[Fig. 5C]**."

We have explicitly added this important caveat to the Discussion to ensure accurate interpretation of the data at lines 515 to 524

"Our analyses reveal that B6C and B75KN domains of the β protein bind LILRB3 by targeting the D3-L3-D4 region of the receptor. While our structural analysis and targeted mutagenesis identified specific amino acids critical for this interaction, we acknowledge that circular dichroism analysis of our LILRB3 mutants revealed alterations in secondary structure, notably an increase in β-strand content. Consequently, we cannot definitively rule out that the loss of interaction is strictly due to the removal of direct contact residues, as there could be an indirect effect of local structural shifts that alter interface

conformations. Future high-resolution structural studies will be highly valuable to definitively map interfaces of these complexes.”
